# Microalgae as a potential raw material for plant‐based seafood alternatives: A comprehensive review

**DOI:** 10.1002/fsn3.4313

**Published:** 2024-09-23

**Authors:** Shahida Anusha Siddiqui, İlknur Ucak, Maliha Afreen, Abhilash Sasidharan, Bello Mohammed Yunusa, Shuva Bhowmik, Ravi Pandiselvam, Tigran Garrievich Ambartsumov, Mohd Asif Shah

**Affiliations:** ^1^ Independent Researcher Germany; ^2^ Department of Animal Production and Technologies, Faculty of Agricultural Sciences and Technologies Nigde Omer Halisdemir University Nigde Turkey; ^3^ Department of Fish Processing Technology Kerala University of Fisheries and Ocean Studies Panangad India; ^4^ Department of Food Science and Technology Federal University Wukari Wukari Nigeria; ^5^ Centre for Bioengineering and Nanomedicine, Faculty of Dentistry, Division of Health Sciences University of Otago Dunedin New Zealand; ^6^ Department of Food Science University of Otago Dunedin New Zealand; ^7^ Department of Fisheries and Marine Science Noakhali Science and Technology University Noakhali Bangladesh; ^8^ Physiology, Biochemistry and Post‐Harvest Technology Division ICAR‐Central Plantation Crops Research Institute Kasaragod Kerala India; ^9^ Faculty of Technology and Innovation Management ITMO University Saint‐Petersburg Russia; ^10^ Department of Economics Kabridahar University Kabridahar Somali Ethiopia; ^11^ Division of Research and Development Lovely Professional University Phagwara Punjab India; ^12^ Centre of Research Impact and Outcome, Chitkara University Institute of Engineering and Technology Chitkara University Rajpura Punjab India

**Keywords:** consumer preference, microalgae, pigments, seafood alternatives, sensory attributes, texture profile

## Abstract

Microalgae presents an inducing potential as a primary raw material in crafting plant‐based seafood alternatives, revolutionizing the landscape of sustainable food production. These microscopic organisms display a rich nutritional profile, presenting an array of nutrients such as essential amino acids, polyunsaturated fatty acids, vitamins, and minerals comparable to those found in seafood. Their versatile nature allows for the replication of seafood flavors and textures, addressing the sensory aspects crucial to consumer acceptance of substitutes. Furthermore, microalgae cultivation requires minimal land and resources, making it an environmentally friendly and scalable option for meeting the increasing demand for sustainable protein sources. The biochemical diversity within microalgae species provides a wide spectrum of options for developing various seafood substitutes. Moreover, advancements in biotechnology and processing techniques continue to enhance the feasibility and palatability of these alternatives. Modern technologies, such as 3D printing, provide convenient and efficient technological options to reproduce the identical texture properties of seafood. As society gravitates toward eco‐conscious food choices, the exploration of microalgae as a core ingredient in plant‐based seafood alternatives aligns with the quest for ethical, environmentally sustainable, and nutritious food sources. This expanding field holds immense potential for reshaping the future of food by offering appealing, cruelty‐free alternatives while reducing dependence on traditional, unsustainable modes of seafood production.

## Graphical abstract

Microalgae presents an inducing potential as a primary raw material in crafting plant‐based seafood alternatives, revolutionizing the landscape of sustainable food production. These microscopic organisms display a rich nutritional profile, presenting an array of nutrients such as essential amino acids, Poly Unsaturated Fatty Acids vitamins, and minerals comparable to those found in seafood. Modern technologies such as 3D printing provides convenient and efficient technological options to reproduce the identical texture properties of seafood. As society gravitates towards eco‐conscious food choices, the exploration of microalgae as a core ingredient in plant‐based seafood alternatives aligns with the quest for ethical, environmentally sustainable, and nutritious food sources.

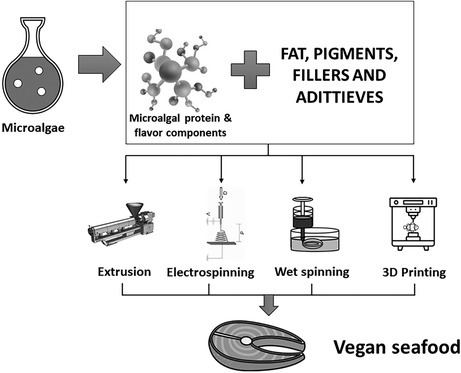

## 1 INTRODUCTION

It is estimated that the worldwide population will increase to 9.7 billion by 2050, and food demand could upsurge by 60% to 100% (Yarnold et al., 2019). Due to the rise in population, other environmental problems could arise, including global warming leading to climate change, emergent pests and diseases, the plateau of animals and crops productivity, and socio‐economic disasters severely influencing the water and land resources for agriculture, ultimately becoming a cause of food scarcity. So, to meet the increasing demand for food security, sustainable agriculture resources are required (Calicioglu et al., 2019). In the years 2020 to 2025, the global market for animal feed is expected to reach 110.29 billion USD, growing at a combined 4% per year (Nagarajan et al., 2021). As a result, there is a need for a sustainable resource for aquaculture and animal feed production that does not put a strain on the existing agricultural food stock. Meat provided by aquaculture and livestock is an important source of protein, supporting 43% of the whole required protein (Yarnold et al., 2019). Algae can be used as a suitable alternative and sustainable source of protein in these circumstances. The sustainability of food is in endangered position due to growing requirements for food, water, and energy. In the meantime, environments and civilizations are susceptible to climate change. Therefore, due to the scarcity of natural resources, effective planning is mandatory for their proficient use, specifically for the conservation of biodiversity (Brasil et al., 2017; Pascoal et al., 2021). In the 20th century, the growth and development of microalgae were enhanced by using this biomass in the diet of human beings (Klamczynska & Mooney, 2017). Microalgae are known as a third‐generation feedstock for the production of biofuel, because of their high productivity, high efficiency of photosynthesis, and greater adaptability to the environment (Levasseur et al., 2020). Human concern for microalgae has reformed constantly, from its usage in conventional medicine, food complements, oils for biofuel production, and the rich value of bioactive compounds as natural substitutes for artificial ones in the marketplace. Microalgal elements such as fatty acids, peptides, and pigments have antioxidant, anti‐cancer, anti‐inflammatory, and skin lightning effects; they also act as a shield against ultraviolet light and have definite wound‐curing properties (Yarkent et al., 2020). The crucial elements of microalgae that are specially used in feed are high protein, carotenoids (chlorophyll, astaxanthin, lutein, and β‐carotene), beneficial pigments, polyunsaturated fatty acids, vitamins, minerals, nutraceuticals, and valued pharmaceutical compounds (Yaakob et al., 2014). However, traditional agriculture has already been changing into present‐day agro‐food systems with production sustainability because it is expected that a growing global population will change the climate from 50% to 90% in 2050 (De Mendonca et al., 2021; Springmann et al., 2018). Therefore, the product must be properly designed by considering its environmental impact, which reflects three major points, including greenhouse gas emanations, proficient use of land, and consumption of water (Singh & Dhar, 2019). According to these parameters, production of microalgae developed to a level that exceeded biomass production and substantial production of by‐products as compared to plant biomass (Geada et al., 2021; Lafarga et al., 2021). Additionally, in aquaculture, fish oil production is proposed to provide omega‐3 food complements. The production of oil from microalgae is sustainable (Laamanen et al., 2021) with less environmental effects as compared to linseed and canola oil (Bartek et al., 2021; Laamanen et al., 2021).

The “European Food Safety Authority” assures that there are no concerns regarding the harmfulness of this unique food, which is considered safe for use in dietary supplements, excluding lactating and pregnant women (Kersting et al., 2021). In Brazil, Chlorella and Spirulina are commercially utilized as food supplements, including dietary supplements for expectant mothers, probiotics, bioactive compounds, and functional supplements for athletes (Brasil et al., 2017). The United States “Food and Drug Administration” has approved several species of microalgae as “Generally Recognized as Safe” foods, including Arthrospira platensis, Arthrospira maxima, Chlorella protothecoides, Chlamydomonas reinhardtii, Haematococcus pluvialis, Dunaliella bardawil, Ulkenia sp., and Schizochytrium sp. (Caporgno et al., 2019; Fu et al., 2021; García et al., 2017). Microalgae produce various primary and secondary metabolites in response to their external environment, playing a significant role in nutrition, health, and industrial applications (De Morais et al., 2015). Microalgae and seaweed have been part of the human diet for centuries, and their use as food additives has increased globally due to their rich nutritional content, including protein, oligosaccharides, carbohydrates, lipids, dietary fibers, vitamins, and minerals. These components offer several beneficial functional effects on human health (Caporgno & Mathys, 2018; Fleurence, 2021; Matos, 2017; Tiwari & Troy, 2015; Torres‐Tiji et al., 2020).

Production of microalgae is mostly focused on East Asia and Southeast Asia. Microalgae and seaweed are broadly consumed as food in East Asia. In other areas of the globe, microalgae are commonly used in the diet of conventional populations as a food additive due to their nutritional properties, less effect on the environment, and as a substitute for animal diet for the conservation of animal life (Boukid & Castellari, 2021; Geada et al., 2021). Microalgae are an encouraging source of proteins, carbohydrates, and omega‐3 fatty acids (Canelli et al., 2020; Niccolai et al., 2019). Microalgae proteins can be applied to yield meat analogs that are suggested to improve the supplication for meat ingestion (Fu et al., 2021). However, innovative plant‐made food elements are required as substitutes for animal protein to come across the consumer point of view, comprising an investigation of configuration properties and techniques of practical standard (Boukid et al., 2022).

## 2 CULTIVATION OF MICROALGAE AND ITS FEATURES

Microalgae farming, comprising photosynthetic cyanobacteria and eukaryotic organisms, offers great value in biomass, although the current variety of microalgae permits the production of a diverse range of goods, including antioxidants, polyunsaturated fatty acids, and pigments, with a focus on biofuel fabrication (Matos, 2019). Microalgae are an important prime production on Earth, mostly occurring in marine systems or freshwater environments, while some are even dispersed in the soil (Schenk, 2021).

Production of microalgae comprises three‐stage procedures, including upstream (cultivation and biomass production), midstream (harvesting of microalgae, drying biomass, cell rupture, and extraction), and downstream (purification of the product from biomass) (Benedetti et al., 2018; Daneshvar et al., 2021; Grossmann et al., 2020; Jareonsin & Pumas, 2021). Production of microalgae is alienated into the cultivation stage and biomass development, then harvesting and processing stages, and then the final product is obtained. Microalgae can grow in different conditions, including photoautotrophic, photoheterotrophic, heterotrophic, and mixotrophic, based on light utilization, organic carbon, and inorganic carbon (Daneshvar et al., 2021). In the photoautotrophic condition, the microalgae use light as an energy source and inorganic carbon to yield organic matter through photosynthesis (Subhash et al., 2017; Verma & Srivastava, 2018). Heterotrophic microalgae can be cultivated in dark conditions by using organic carbon to produce greater cell densities, a higher growth value, and a higher productivity of biomass (Siddiqui et al., 2023). Microalgae that grow in a mixotrophic condition have both photoautotrophic and heterotrophic characteristics. By using light, carbon dioxide, and organic carbon‐taking advantage of both photoautotrophic and heterotrophic conditions, they produce higher amount of lipid and biomass and less photoinhibition (Ananthi et al., 2021; Cheirsilp & Torpee, 2012).

For an efficient production of microalgae biomass, many risky features must be concerned, including growth media, strain selection, cultivation system, and related biotic and abiotic elements, such as temperature, dark–light cycle, wavelength, and intensity of light. Based on data collected from bioreactors, volume and configuration of the cultivation system, environmental variables, and different types of wastewater generated have been collected (Daneshvar et al., 2021). A minor difference in a few of these environmental dynamics can produce immense modifications in the quality and yield of algae biomass, specifically in terms of food end products (Feng et al., 2014; Guccione et al., 2014).

### 2.1 Microalgae cultivation systems

The profitable cultivation of microalgae uses a distinctive type of bioreactor, which gives ideal growth conditions for high yield output. Nowadays, there are two different types of microalgae growing methods in use, including “open raceway ponds” and “closed photobioreactors” based on requirements. Treatment of wastewater has normally been done in open raceway ponds, while therapeutic and value‐added bioactive products have been formed in closed photobioreactors (Callegari et al., 2020).

#### 2.1.1 Open raceway pond

The open raceway pond has been used for the last 40 years for a higher yield of profitable microalgae by means of an open pond reactor system. The most common species of microalgae that are cultivated in an open raceway pond are Spirulina, Chlorella, Haematococcus, and Dunaliella, by culturing them in pure water or wastewater, with carbon dioxide capture technique used in more or less all power plants (Shekh et al., 2022). An open raceway pond could be constructed by plastic or concrete and positioned on the ground otherwise excavated into the earth and walled to retain fluids out of the mud range. An open raceway pond could be formed with a single passage or multipassages, and the depth of the cultivation medium is normally between 15 and 50 cm adjusting baffle and paddlewheel, that controls fluid flow, keeps algal cells moving around in the growth medium, and prevents sedimentation. Adequate movement of algae cells gets carbon dioxide and sunlight from the surrounding environment. In this open raceway pond, the productivity of biomass ranged from 60 to 100 mg L^−1^ day^−1^ (Kassim, 2015).

#### 2.1.2 Closed photobioreactors

The closed photobioreactor cultivation systems are better than the open‐pond reactor cultivation systems. It needs nutrients such as nitrogen, phosphorus, silicon, and iron for its growth medium (Callegari et al., 2020). A description of two types of photobioreactors is given.

##### Flat‐panel photobioreactor

Flat‐panel photo bioreactor is a quadrilateral‐shaped box that is used for the cultivation of algae. It could be castoff outdoors or indoors, based on light availability. Glass, plastic bags, and polycarbonate are used to make flat‐panel photobioreactors that are semi‐transparent or transparent. A flat‐panel photobioreactor has gained more attention for generating photosynthetic microorganisms owing to its large enlightenment surface area (Suparmaniam et al., 2019). A flat‐panel photobioreactor has been made up of transparent materials, so it can use maximum energy from solar light. Consequently, a light pathway that is amply short produces a great light surface with actual distribution of light, diffusion, and consumption (Behera et al., 2019). It has been recommended that flat‐panel photobioreactor attain great photosynthetic efficacy, so these are appropriate for sweeping cultivation of algae (Sarker, 2021).

##### Tubular photobioreactor

Tubular photobioreactors have the maximum surface–volume ratio, which is beneficial for the light exposure of microalgae. Tubular photobioreactors are constructed by using tubes of polyethylene or glass materials in an utmost distinctive shape of a twisting loop organized in a single design (Hijazi et al., 2020). An additional unique feature of tube reactors is their circular mixing efficacy. Except the tubes, a gas conversation system helps in the consumption of fresh growing media, the supply of carbon dioxide, and the cycling of cooling water. Tubular photobioreactors are unique from other photobioreactor systems by the diameter and length of the tubes, their recirculation nature, flow speed, and systematic receiving of light. In tubular photobioreactors, airlift and air‐pump devices are mostly used to ventilate and blend the cultures. Tubular photobioreactors are perfect for outdoor high‐quantity algal growth due to their broad illuminated surface area (Solimeno et al., 2017).

### 2.2 Harvesting and processing of microalgae

The proficient assembly of algal biomasses is a competent task for the algal‐based biofuel‐producing procedure (Milledge & Heaven, 2013). Harvesting of biomass is a critical step, as the separation of the slurry from mass media facilitates advanced downstream processing by extracting preferred products at a cost of 20%–30% of the total production cost. The harvesting procedure is chosen based on the shape, size, and flexibility of the entity, such as the essential quantity and quality of the output (Muylaert et al., 2017). Traditional physical methods and some chemical methods are used for the harvesting of microalgae. Centrifugation, sedimentation, filtration, and flotation are examples of physical procedures, whereas flocculation is an example of a chemical‐based process (Vasistha et al., 2021). The techniques have been reviewed in the following section.

#### 2.2.1 Physical methods

The commonly used physical method for harvesting microalgae is centrifugation; however, the output is contingent on cell size, speed, and time of the process. High sleek pressures are used during the process of centrifugation, which breaks the algal cells and discharges algae oil into the medium. There are many different types of industrial centrifugation methods that exist. A round bowl centrifuge method is applied for deferrals with a small solid pallet, while a vessel centrifuge method is applied for deferrals with a big solid pallet (Muylaert et al., 2017). Many centrifuges have been scrutinized for the separation of microalgae, including disk pile centrifuges, nozzle type centrifuges, tubular centrifuges, vessels, pierced and non‐pierced bag centrifuges, and hydrocyclones (Pahl et al., 2013). Disc pile centrifuge methods are only used in industrially valued product reclamation from algae, but they consume high energy as compared to the products they produce (Milledge & Heaven, 2013). Vessel centrifuge methods are highly effective but exhaust a high amount of energy, while hydrocyclone centrifuges require less attention but could not be used on a broad scale because of their great consumption of energy and costs.

##### Filtration

Other methods used for harvesting algal biomass are diverse types of filtrations, involving microfiltration, ultrafiltration, and vibrant membrane filtration (Nurra et al., 2014). Filtration involves a pressure droplet all through the method to be sustained, so that the medium fluid passes through specific filters like screens, membranes, and microfilters to hold the biomass of microalgae. Additionally, by applying many kinds of forces like pressure, gravity, and vacuum, the required pressure droplet might be attained (Barros et al., 2015). Diverse membrane procedures could be used reliant on the path of the feedstuff flow, the pore size of the membrane, and which forces castoff in the filtering procedure (Singh & Patidar, 2018). For the collection of large microalgae cells like filamentous microalgae, the microfiltration method is used. Microfiltration separates microalgae cells from cultivation medium at an operative pressure of 1–2 bar, but in ultrafiltration, the membrane could hold biological molecules such as carbohydrates and proteins at an operative pressure of 5 bar (Fasaei et al., 2018).

##### Flotation

Flotation is a procedure for efficient and concentrated recovery of microalgae (Laamanen et al., 2016). This procedure needs less space, has a rapid operational time, is suitable for handling large amounts of cultivated media, and requires less cost and energy. This method used collector chemicals, which could create more hydrophobic elements to enable the combination of air bubbles and particles to develop its efficacy. These collector chemicals are known as anionic, non‐ionic, cationic, thio‐compounds and surfactants or flocculants (Singh & Patidar, 2018).

#### 2.2.2 Chemical methods

Chemical methods that are used for the harvesting of microalgae are known as flocculation processes, based on surface charges and electrostatic connections. Flocculation is a combining method used in the treatment process to eliminate colloidal elements from wastewater. The flocculation process is suggested for harvesting many species of microalgae at laboratory and industrial scales due to its lower cost (Vandamme et al., 2013). In this process, diverse types of coagulants are added to the cultivation medium to form aggregates from cells and deferred solvent atoms, which enable the sedimentation process. Different types of flocculation techniques, including self‐flocculation, chemical flocculation, and bioflocculation exist (Matter et al., 2019). Flocculation is a very effective method for harvesting biomass, and it is executed by adding bioflocculants or chemical flocculants that have resilient molecular interactions with negatively charged particles of microalgae. Those particles that are used in the flocculation process could be metal cations like aluminum or polyacrylamide by‐products (Blockx et al., 2018; Ruggeri et al., 2021). In general, the flocculation method involves neutralization, patching, sweeping, and bridging (Chen et al., 2015). For the harvesting process, negatively charged microalgae cells interact with the chemical flocculants that are positively charged, which facilitates effective harvesting of microalgae (Mathimani & Mallick, 2018).

##### Bio‐flocculation

This type of flocculation procedure uses biopolymers, including “extracellular polymeric substances and γ‐glutamic acids,” as bioflocculants (Barros et al., 2015; Chen et al., 2019). The bioflocculation process has been categorized into bacterial, fungi, and plant‐based flocculation. Many studies recommend that microbes such as bacteria are inordinate drivers of bioflocculation procedures. The effective results of bio‐flocculation were obtained by harvesting *Chlorella vulgaris* and nutrient removal from whole seafood‐generated wastewater (Nguyen et al., 2020).

##### Autoflocculation

Autoflocculation involves the self‐attachment of microalgal cells, which attach to each other and form floc. Normally, the autoflocculation process could happen due to the pressure of the environment, like variations in pH, temperature, nutrients, and amounts of magnesium and calcium ions in the cultivation medium. Flocs of microalgae cells form due to negatively charged ions on the surface of cells with easy electrostatic connections (Wan et al., 2015). It was found that the harvesting of *Nannochloropsis* sp. was amplified up to 95% when the medium pH was 10 (Schlesinger et al., 2012). In another study for the harvesting of *Chlorella vulgaris*, the hydroxide ions of Mg, K, and Ca were added to the cultivation medium for flocculating, and above 98% biomass was obtained (Alam et al., 2016).

#### 2.2.3 Processing of harvested biomass

The final product of microalgae after harvesting, processing, and drying is normally an orange or green powder. Different types of methods are used to dry the biomass of microalgae, including drum drying, spray drying, solar drying, freeze drying, and convective drying (de Farias Neves et al., 2019). These microalgae powders were finally used in the form of pastilles or pills or can be served as a food element. This type of final product also allows easy transport and long‐term constancy. Additionally, *Schizochytrium* sp. is cultured for oil productivity in particular conditions of pH, temperature, time, and aeration. The biomass of *Schizochytrium* sp. is attained through enzymatic hydrolysis, centrifugation, and then clarified by filtration for the removal of solid constituents. Finally, oil is obtained through neutralization, clarification, and deodorization at high temperatures ranging from 160 to 260°C.

### 2.3 Potential use of microalgae

Microalgae provide a rich, quality feed supply for livestock and a substitute for primary crops. Microalgae are easy to cultivate and have a high growth level, so they have not only decreased the cost but also conveyed socio‐economic benefits. The demand for animal food and proteins is increasing with the continuous rise in population. As well, microalgae, a feed additive for livestock, can offer good energy and a substantial basis for rumen microbial fermentation and efficiently decrease in vitro methane production (Maia et al., 2016).

**FIGURE 1:**
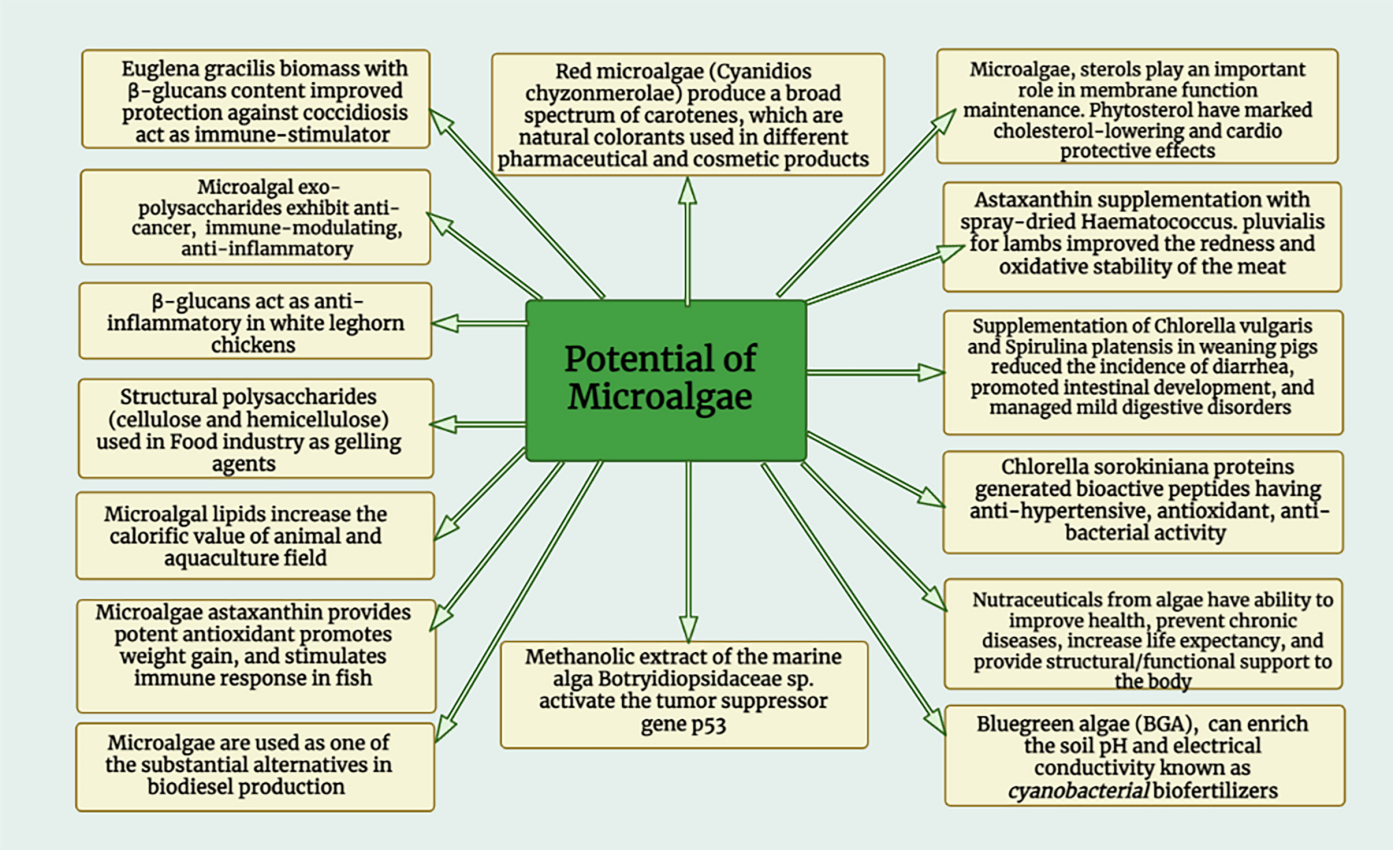
Potential uses of microalgae.

Microalgae biomass could be used in several applications, including the food industry, pharmaceutical, cosmetic, and biofuel production (Carrasco‐Reinado et al., 2019; Fleurence & Levine, 2018). The investments in microalgae have extended to over nine hundred million globally, and these developments might be important to developing biorefineries from microalgae (Chen et al., 2019). Descriptions of numerous potential applications of microalgae are given below, and presented in Figure 1.

#### 2.3.1 Agriculture

Microalgae are used as biofertilizers in the agriculture market in different ways. The benefits of microalgae application in agriculture include the following:

##### Nitrogen fixation

Blue‐green algae could resolute atmospheric nitrogen and convert it into biological nitrogen forms, which are further used by developed plants. So, the use of blue‐green algae in soils upsurges the accessibility of nitrogen, which is an important nutrient for plant growth and also increases crop yield (Baweja et al., 2019; Renuka et al., 2018; Sharma et al., 2012; Win et al., 2018). Presently, many free‐living species of blue‐green algae are identified as significant constituents of cyanobacterial bio‐fertilizers (Renuka et al., 2016). The use of a biofilm produced by the cyanobacterium *Anabaena torulosa* was shown to improve nitrogen accessibility in agricultural soil in a wheat crop (Swarnalakshmi et al., 2013). Blue‐green algae cyanobacteria are also recognized as having the capability to create soil crusts that evade nitrogen leakage, minimizing the stress of water contamination with nitrogen (Renuka et al., 2018).

##### Nutrients' availability in soils

The use of microalgae and cyanobacteria in crop soils upsurges the accessibility of further important plant nutrients. They take phosphorus and nitrogen from diverse surrounding environments like photosynthetic microorganisms, and stock them in their biomass. Microalgal or cyanobacterial biomass may be regarded as a substantial source of these nutrients, which are crucial for the growth and development of higher plants (Ronga et al., 2019; Sharma et al., 2012; Win et al., 2018). Additional important microelements, including potassium, magnesium, iron, and sulfur, are also important for plants' growth and development and are obtained from the biomass of microalgal or cyanobacterial organisms. These essential elements are normally used in redox reactions, and important for metabolism of plants (Manjunath et al., 2016; Marks et al., 2019; Renuka et al., 2018). Biomass and microalgal–bacterial flocs in the tomato crop were treated with *N. oculata*, and its impact on fruit development and soil composition was evaluated. The researchers came to the conclusion that the presence of photosynthetic organisms increased the availability of nitrogen, phosphorus, and potassium in agricultural soil and improved the quality of fruit through an increase in plant carotenoids and sugar content (Coppens et al., 2016). In a different study, okra crops were treated with cyanobacterial–bacterial biofilms, which increased the amount of iron and zinc in the soil and led to better weight and root production (Manjunath et al., 2016). Microalgae and cyanobacteria are also used for reclamation of impaired soils by modulating their pH, soils' salinity control, and the removal of heavy metals from soils (Pan et al., 2019; Prasanna et al., 2014; Priya et al., 2014; Sharma et al., 2012). They are also used for remediation of soil and an upsurge in soils' fertility and quality features, like porosity, ventilation permeability, aggregation, and humidity (Chatzissavvidis & Therios, 2014; Kumar et al., 2015; Win et al., 2018).

##### Direct growth stimulation

Microalgae and cyanobacteria could directly enhance plants' development and growth through advancements in germination levels and characteristics of plants, including enlarged root and shoot length, enlarged leaf area, and greater nutritional quantities. These developments are attained through the metabolite action of microalgal and cyanobacterial species, which consequently activate many metabolic reactions, including respiration, synthesis of nucleic acid, chlorophyll production, and photosynthesis (Górka et al., 2018). Regularly used metabolites consist of plant growth stimulators like amino acids, phytohormones, vitamins B12, biotin, and polypeptides (Sharma et al., 2012).

#### 2.3.2 Biofuel

Aerobic and anaerobic approaches are normally used for the production of hydrogen. The cells endure extreme physiological modifications during these two stages, followed by hydrogen production. Lastly, the by‐products of algal biomass are produced after the collection of hydrogen. Thermochemical procedures include hydrothermal liquefaction, and just lipids are recuperated by gasification and pyrolysis, causing the loss of carbohydrates and proteins. This method has been used in exceptionally high conditions of pressure and temperature. Unusually, the biomass of microalgae could be transformed into methane without a dewatering process or energy input. Therefore, the whole energy is brought into being increased through biogas production. The distinctive microalgae have a considerable quantity of organic material and are converted to anaerobic digestion at a temperature of 35°C. Anaerobic digestion is an important method for biofuel production from microalgae. Additionally, *Chlamydomonas* sp. produces hydrogen through the hydrolysis process, which is reflected as a biodegradable energy source (Khan et al., 2018).

The triacylglycerol's and neutral lipids from microalgae have been broadly discovered for the production of biofuel for many years. Neutral lipids could be removed from the dry, lipid‐rich biomass of microalgae by using many solvents, including organic solvents, switchable solvents, ionic liquids, supercritical solvents, and deep eutectic solvents, and then it is converted to biodiesel through transesterification (Lee et al., 2021). In some procedures, direct wet microalgal slurry is used for esterification; for example, dimethyl ether abstraction of wet *Nannochloropsis oculata* biomass with increased production of fatty acids, lipids, and methyl ester (Wang, Oshita, & Takaoka, 2021).

#### 2.3.3 Cosmetics

Microalgae and cyanobacteria with high anti‐oxidizing properties of colorings have very important value in the cosmetic industry. This generally comprises photosynthetic pigments like carotenoids, for instance, astaxanthin from *Haematococcus pluvialis*, which has a red color, and β‐carotenes from *Dunaliella salina*, which have antioxidant properties that are ten times more potent than other carotenoids and a hundred times more solid than alpha‐tocopherol (Hamed, 2016). Phycoerythrobilins from Porphyridium and Spirulina and blue pigment phycocyanobilins with antioxidant activity could be castoffs in the embellished cosmetics industry involving lipstick and eye‐liner (Hamed, 2016). Another pigment, canthaxanthin is mostly gotten from Nannochloropsis sp. and sold out as tanning pills (Koller et al., 2014). Red microalgae (*Cyanidios chyzonmerolae*) yield a wide‐ranging variety of carotenes, which are natural pigments applied in various cosmetic products (Kannaujiya et al., 2019).

#### 2.3.4 Food and feed

Microalgae are the best source of food and feed ingredients for humans and animals, as they contain high quantity of protein ranging from 6% to 71% of dry matter (Caporgno & Mathys, 2018); for instance, Arthrospira and Chlorella species have the best protein values. Despite the carotenoids, proteins, and lipid, microalgae also comprise an excessive number of bioactive compounds, like minerals, vitamins, and fibers. Further, microalgae, specifically marine algae, also contain a considerable amount of minerals, including calcium, potassium, magnesium, iron, and iodine. It was reported that blue‐green algae or red algae have a higher quantity of protein as compared to green or brown algae (Cardoso et al., 2020).

The beneficial nutritional characteristics of algae approve their capability to be used as feed in the livestock industry, especially the high level of carotenoid in many algal species that increases the color of skin and egg yolks in poultry. It was stated that a small addition of microalgae biomass to poultry feed could securely exchange traditional proteins and have a positive influence on animal physiology (Yaakob et al., 2014). It was also proved in another study that Spirulina platensis was completely or partially used as an alternative to bean protein in the diet of dairy cows. It was observed that microalgae feeding dairy cows have augmented milk fat as compared to soybeans feeding dairy cows (Lamminen et al., 2019). Omega‐3 fatty acid supplements obtained from microalgae as food could also decrease the dosage of antibiotics, which have a bad effect on the health of human beings (Moheimani et al., 2018). Additionally, microalgae with a rich amount of dietary fiber and polysaccharides are also stated to constrain pathogenic bacteria and be beneficial for intestinal health by controlling the abdominal microflora (de Jesus Raposo et al., 2016).

Structural polysaccharides, including cellulose and hemicellulose, present in the cell wall of microalgae give firmness to the cell structure for protection against environmental intrusion. These types of microalgal polysaccharides are used in the food industry as gelling agents because of their promising rheological features (Bernaerts et al., 2018). One more type of structural polysaccharide, entitled β‐glucans, has possible functional features (Schulze et al., 2016). Broiler chickens fed with 55% β‐glucans containing dried *Euglena gracilis* microalgae presented enhanced protection against coccidiosis because of its immune‐stimulating characteristics (Levine et al., 2018). Those microalgae that have polyunsaturated fatty acids have been used as aquaculture feeds to enhance the fatty acid quantity of oily fish (Levasseur et al., 2020).

#### 2.3.5 Medicine

Microalgae *Chlorella vulgaris* have polysaccharides with distinctive structural properties, including monosugars, uronic acids, sulfate content, and proteins. The refined polysaccharides assisted in the production of silver nanoparticles, which have possible anti‐microbial activity (El‐Naggar et al., 2020). These purified polysaccharides also have anti‐aging and immune‐modulatory properties in *Caenorhabditis elegans* biomass (Liu et al., 2018).

Exo‐polysaccharides of microalgae are involved in cell signaling or intercellular communication to provide protection from adversarial environmental situations (Pierre et al., 2019). Microalgal exo‐polysaccharides are recognized due to their immune‐modulating (Wu et al., 2016), anti‐cancer (Halaj et al., 2019; Zhang, Liu, & Chen, 2019), anti‐inflammatory (Capek et al., 2020; Xiao et al., 2018), and prebiotic (Atik et al., 2021; Çelekli et al., 2019) actions. Microalgae phytosterols have a significant role in the maintenance of membrane function. Phytosterols have noticeable cholesterol controlling and cardioprotective properties, along with antioxidant, anti‐inflammatory, and anti‐cancer activity (Levasseur et al., 2020).

Microalgae also have potential sources of bioactive peptides (Carrasco‐Reinado et al., 2020). Hydrolysis of *Chlorella sorokiniana* proteins by using proteolytic enzymes bromelain, pepsin, and thermolysine produced bioactive peptides with different molecular masses having anti‐hypertensive and anti‐bacterial actions (Tejano et al., 2019). Carotenoids of microalgae like astaxanthin have antioxidant and anti‐inflammatory potential, improve weight gain, and activate immune reactions (Lu et al., 2021). Microalgal biomass also contains dietary fibers that hold and inhibit the absorption of extra nutrients in the intestine, endorsing the prevention of diabetes mellitus, high serum cholesterol, and colon cancer (Kaczmarczyk et al., 2012). Methanol extract of the marine algae *Botryidiopsidaceae* sp. was presented to stimulate the apoptotic protease caspase 3 and tumor suppressor gene p53, so it is used for cancer therapy (Suh et al., 2017).

## 3 THE USAGE OF MICROALGAE AS AN ALTERNATIVE FOOD SOURCE

Hunger is a global issue that upsets one out of nine individuals in the world, and the most important concern is lethality due to a deficiency of proper protein and caloric intake, known as protein–energy malnutrition (Massironi et al., 2023). Moreover, overfishing from marines and the decline of arable land will also increase this issue, proposing that we must manage new, maintainable and accessible sources that can fulfill the growing requirements of feed without enough land or fresh water supply. Microalgae have the ability to be a maintainable food and feed way out, but still, more advancement is necessary to convey these organisms used in normal food assembly. It is already stated that algae have very important nutrients, but it is not enough for human satisfaction to include algae in their diet as a regular and significant element. Organoleptic properties, such as flavor, texture, and aroma, will be important aspects for humans, and even for animals, in approving algae as their diet element. It is essential to contrive algae to make it more satisfying and attractive by improving its smell and taste (Wells et al., 2017).

**FIGURE 2:**
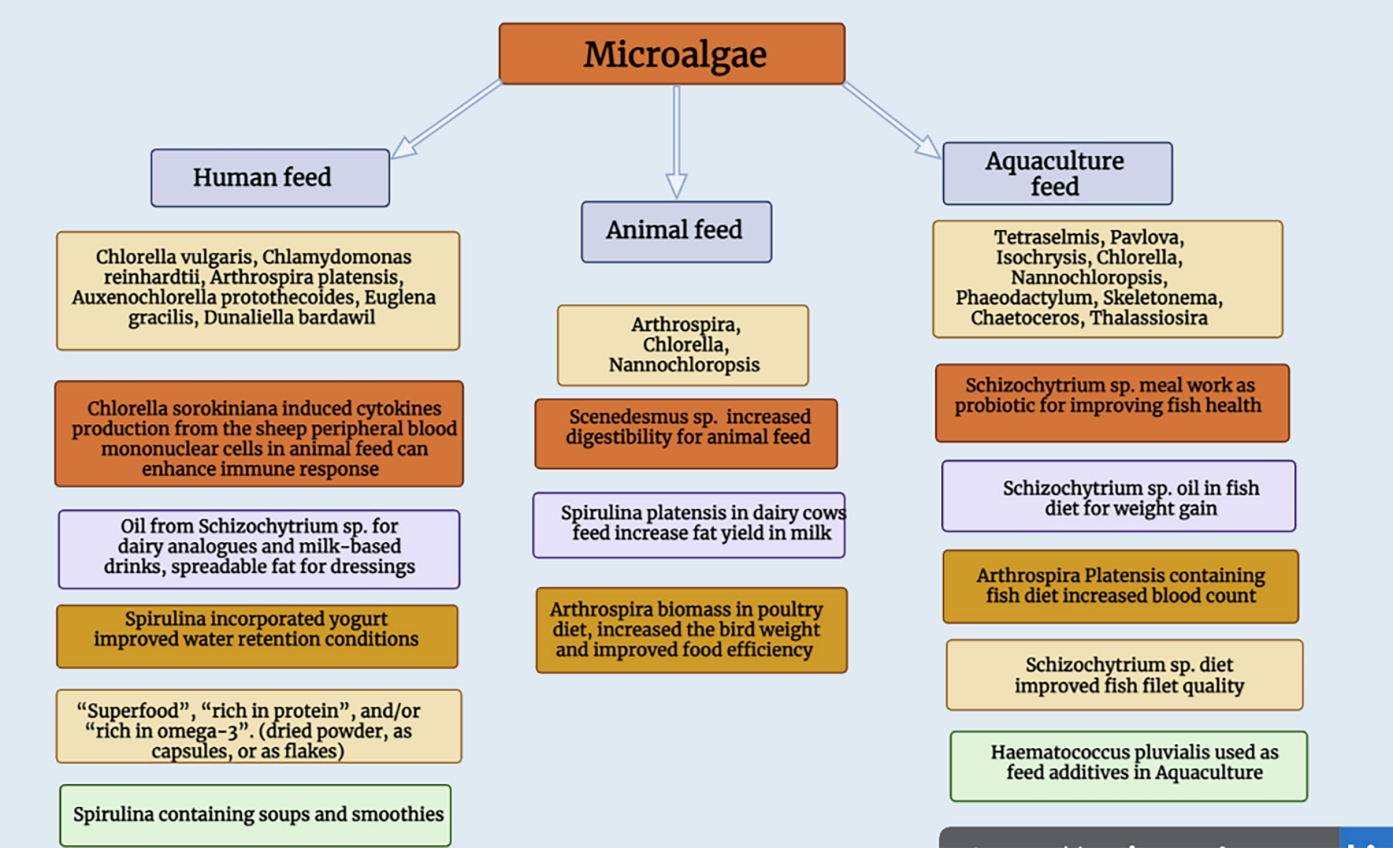
Uses of microalgae in human and animal feed.

Presently, about 22,000 to 25,000 tons of microalgae biomass are generated for human nutrition and animal nutrition, and about 30% of this is acquired for feed productivity, as shown in Figure 2 (Levasseur et al., 2020). In recent times, microalgae have been used as a healthy food and the key products founded on microalgae are food complements and additives. For example, docosahexaenoic acid is used in fortifications of milk, juices, and other drinks, particularly for children and infants (Araújo & Peteiro, 2021; Cerón‐García et al., 2018; Singh et al., 2019).

### 3.1 Usage of microalgae as human food

Microalgae are approved as a food constituent by the “European Food Safety Authority” (Vieira et al., 2020). *Chlorella vulgaris*, *Arthrospira platensis*, *Chlamydomonas reinhardtii*, *Euglena gracilis*, *Auxenochlorella protothecoides*, and *Dunaliella bardawil* are assumed to be “generally regarded as safe” by the United States Food and Drug Administration (Torres‐Tiji et al., 2020). Although it is generally believed that *Chlorella* microalgae are safe for human consumption, different countries have different accepted *Chlorella* species. For example, *C. protothecoides* is accepted in Japan and the United States, *C. vulgaris* is accepted in Europe and Japan, *C. pyrenoidesa* is accepted in Europe and China, *Dunaliella salina* is accepted in China and Canada, and *C. sorokiniana* and *C. regularis* are both accepted in Canada (Torres‐Tiji et al., 2020). Additionally, *Euglena gracilis* is considered a safe food in Japan, Canada, and China. Omega‐3 oils are naturally produced by algae, and because natural fish production is going to decrease, algae oils are used as nutritive complements for direct human consumption instead of fish (Koller et al., 2014).

Over the previous decade, numerous noticeable companies involved in the production and sale of food have started to include themselves in the manufacturing of microalgae‐containing foods all over the world. For instance, it is promising to find “Follow Your Heart” Vegan Egg in Europe, which is manufactured from natural microalgae in the Netherlands, and Spirulina Filled crackers can be easily found in Asia. Some other algae‐containing food products that can be found in America include Züpa Superfood Soup, Spirulina‐comprising avocado soup, and Roo'Bar made up of Chia and protein‐rich Spirulina algae, available in the Canadian food market (Thangaraj et al., 2023).

Most microalgal biomass food supplements are presently commercialized in the form of capsules, tablets, or powdered forms and are endorsed as protein‐rich, omega‐3‐rich “superfoods.” These superfoods mostly comprise the biomass of Spirulina microalgae. For instance, “Dragon Superfoods Spirulina powder” found in the form of powder, “Apollo Hospitals Life Spirulina” found in the form of capsules, and “Label Spirulina La Ferme de Bancel” found in the form of flakes. Other species of microalgae like carotenoids‐rich “Laboratoires Lierac Sunissime” are found in the form of tanning capsules got from *Dunaliella salina*; astaxanthin and lutein containing “Jeil Health Science Eye treasure,” got from *Haematococcus* and is proved to relieve eye strain (Goswami et al., 2021).

Now it is a global trend to assimilate microalgal biomass into customary food products, like bread, and many other food products have been propelled all over the world. For instance, British buyers can purchase “Mavericks greenzilla breadsticks,” containing 2% Spirulina, and “Spirulina‐filled crackers” found in the Malaysian market (Lafarga et al., 2021). Many food products contain Spirulina or Chlorella species of algae as a colorant. Additionally, most of the microalgae‐containing food products are dedicated to vegan consumers and to those consumers who select to buy biological or ecologic food products. Alongside the existence of seafood aroma properties, it is significant to avoid consuming off‐flavors in plant‐based seafood substitutes like floral, grassy, earthy, or rotten aromas. Grassy and earthy aromas are usually found in microalgae, though, intense grassy aromas are perceived in *N. oceanica* and *S. costatum*. Lastly, the earthy aromas were strongest in *C. vulgaris* and *S. costatum*. Based on aroma profiles, *P. tricornutum*, *R. salina*, and *T. chui* microalgae have the utmost potential for plant‐based seafood taste since their greater aroma score for seafood features, whereas lesser scores for grassy, floral, earthy, and rancid aromas (López‐Pérez et al., 2017). Some microalgae are also distinctive due to their seafood umami essence; therefore, they are a substitute for traditional seafood (Southey, 2021).

### 3.2 Usage of microalgae as animal's food

At the time of the First World War, on the French Atlantic coastline, animals were fed with algae because of the deficiency of forage and oats. For the duration of 20 years from 1960 to 1980, substantial quantities of brown algae were supplemented with animal feed. Figure 2 depicts the potential use of microalgae in human and animal feed applications. The primary research on nutritional supplementation of microalgae in animals proposed for human ingestion presented that microalgae have better digestibility, acceptability, and absorption. Further studies confirmed the useful effects of the algae, while supplemented to the feedstuff at 5% to 10% concentration (Coffey et al., 2016; Fleurence et al., 2012). Several studies have assessed the bioaccessibility and digestibility of microalgae biomass as feed complementation in many animals involving chickens, sheep, and rats, but some other studies have focused on figuring out the bioavailability of microalgae compounds in aquaculture animals, including Atlantic salmon, rainbow trout, Pacific white shrimp, and Nile tilapia. There was a study on the metabolizable energy and digestibility of amino acids from *Arthrospira platensis* microalgae, which is used as a food ingredient in broiler chicken diets (Tavernari et al., 2018). It was concluded that substituting corn meal and soybeans with up to 7.5% to 10% Arthrospira algae biomass in the poultry diet in the initial 3 weeks of life enhanced the bird weight and food efficacy (Austic et al., 2013). Additionally, some species of microalgae, like *Pavlova* sp. and *Isochrysis* sp., are used in aquaculture as living feedstuffs for the larval phase, but on a small industrial scale. However, in recent times, microalgae species are being considered as feedstock constituents for fingerlings and fully developed fishes (Hodar et al., 2020).

When aquaculture species were nourished with a diet comprising less and moderate 2%–10% concentrations of microalgae biomass, the weight gain was improved as compared to the control. In one experiment, Atlantic salmon was fed with 5% *Schizochytrium* sp. oil‐containing diet, and 31% higher weight gain was observed as compared to a control diet (Wei et al., 2021). Several additional studies have shown that consuming a diet high in microalgae can increase fish survival. The seahorses' and oysters capacity to survive was improved by the inclusion of microalgae species such as *Pavlova* sp., *Chaetoceros* sp., *Nannochloropsis oculata*, and *Isochrysis* sp. in their meal (Nagappan et al., 2021). The improvement in fish survival ability by using microalgae‐composed feeds might also be associated with their functional properties, involving the influence of prebiotics, probiotics, antibacterials, antivirals, and immunostimulants.

## 4 USE OF MICROALGAE AS AN ALTERNATIVE FOOD SOURCE IN DIFFERENT COUNTRIES

Microalgae is considered as a reliable natural source of alternative food that could satisfy the rising demands for food in different countries (Barka & Blecker, 2016). Microalgae production is mostly cultivated globally and is estimated to be approximately 32 million tons of fresh weight worth more than 6 billion USD (Neori, 2011), with 85% said to be a reliable natural source of alternative food products for human consumption (Lähteenmäki‐Uutela et al., 2021). Figure 1 depicts the potential applications of microalgae.

### 4.1 Consumer acceptance of different types of algal‐based foods

Microalgae‐based food products are more regarded as functional or health‐related foods that can be offered to consumers as superfoods and dietary supplements (Rösch et al., 2019; Siddiqui, Alvi, et al., 2022). Microalgae‐based foods are still quite uncommon in different parts of the world. Consumer education is necessary in communicating and enlightening consumers on the nutritional values of the added microalgae and its economic potential (Parniakov et al., 2018). However, if the microalgae‐based food products are properly utilized to their potential, it will be interesting to find out how consumers will comfortably accept the new microalgae products. The type of product to which microalgae is added is called a carrier product, and this carrier product plays a vital role in how consumers perceive the added microalgae (Siddiqui, Bahmid, et al., 2022). There are different types of carrier products that are more likely to be suitable for microalgae addition, as shown in Table 1. Flavor, texture, and appearance are the most important attributes of the algae food, which are directly associated with consumer acceptability.

**TABLE 1:** Most consumed microalgae‐based product in different countries.

Country	Product name	Product application	Amount consumed per year	References
China, India, and Taiwan	Dried Spirulina biomass	Food and beverages	>12,000 tons per year	Mu, Mehar, et al. (2019)
	Dried Chlorella biomass	Food and beverages	5000 tons per year	
	Dried *D. salina* biomass	Carotenoids	3000 tons per year	
	Dried *Aphanizomenon flos*‐aqua biomass	Food products	1500 tons for food	
	Dried *H. pluvialis* biomass	Astaxanthin	700 tons for astaxanthin	
	Dried *Crypthecodinium cohnii* biomass	DHA	500 tons of DHA	
	Dried *Schizochytrium* biomass	DHA	20 tons of DHA	
Taiwan	Powders, nectar, and noodles	Chlorella Manufacturing	2000 tons per year	Andrade et al. (2018)
Germany	Chlorella powders	Chlorella powder	2000 tons per year	
USA	Spirulina Powders, extracts	Spirulina powders, extracts for food application	3000 tons per year	
USA	Powders, beverages, extracts	Spirulina powders, beverages, and extracts for food application	3000 tons per year	
Myanmar	Spirulina chips, pasta, and liquid extract	Spirulina powder	3000 tons per year	
Japanese	Konbu (*Saccharina stackhouse*)	Glutamate (2%–3% dry weight)		Mouritsen et al. (2019)
India	Chicken rotti	To improve the nutritional, physicochemical and sensory properties of chicken rotti		Parniakov et al. (2018)
Australia	β‐Carotene	Food additive and ingredient		Matos (2019)
United States	Omega‐3 eicosapentaenoic and docosahexaenoic acids	Dietary supplements or food ingredients		
China, Japan, Europe, and the US	Chlorella	Food application		Sousa et al. (2008)
Mexico and Africa		Food application	3000 tons/year	
Italy	Rice pasta, sfogliatine, and grissini	Food application		Matos (2019)
	Chocolate bars and biscuits	Food application		
	Novel yoghurt	Novel yoghurt products		
	*Spirulina maxima* fresh spaghetti	Food application		

#### 4.1.1 Factors affecting consumer acceptance

Due to the rising concern for sustainability and health, consumers are becoming more and more interested in the ingredients used in the food products they buy (Rune et al., 2022). Socio‐cultural barriers such as religious and socio‐economic are among the few factors that make adults resist the acceptance of novel foods with microalgae incorporation, unlike children, who are mostly unconcerned and more willing to accept novel foods with microalgae incorporation in the previous decades (Siddiqui, Zannou, et al., 2022; Sousa et al., 2008). In recent decades, there has been no evidence of rejection of food products from microalgae as a result of socio‐cultural barriers, compared to cultivated meat and edible insects, which are also regarded as sustainable alternative protein sources (Rösch et al., 2019; Rumin et al., 2021). However, due to consumer concerns that microalgae may be polluted by toxins, heavy metals, or other pollutants, coupled with a lack of visibility and communication of the microalgae with consumers, small socio‐cultural obstacles may potentially restrict consumer knowledge (Rumin et al., 2021).

#### 4.1.2 Economical, ecological, and nutritional significance of microalgae compared to seafood

Seafood alternatives are important in addressing the dietary requirements of vegans while at the same time addressing the environmental and ethical apprehensions correlated with conventional seafood utilization. The ever‐increasing number of people embracing a plant‐based lifestyle emphasizes the significance of a balanced and benevolent substitute for seafood. The nutritional value added to the normal plant‐based diet of the vegan population by these alternatives is also an important point. Plant options like seaweed and algae provide vital nutrients like protein, omega‐3 fatty acids, and vitamins that are otherwise missed out on by a vegan diet, which excludes seafood. Schade et al. (2020) compared the nutritional parameters of selected microalgae and fish species. The microalgae reported a smaller calorific value and fat content when compared to seafood. The eicosapentaenoic acid (EPA) content was higher in microalgal species than in seafood compared with lower amounts of docosahexaenoic acid (DHA). Other studies have observed that the EPA and DHA levels in microalgal lipids are considerably higher and less contaminated than those from seafood sources (Lu et al., 2021). Many studies show that, depending on the species and growing conditions, microalgae could contain a considerably higher percentage of protein, even up to 60% (Bleakley & Hayes, 2017). Microalgal proteins are also observed to contain both essential and nonessential amino acids, making them one of the ideal vegan protein sources (Barka & Blecker, 2016). Microalgae are also excellent sources of vitamins like E, C, B6, B1, B2, B7, and A (Koyande et al., 2019). Microalgae are also known to synthesize pigments like astaxanthin, phycobiliproteins, phycoerythrin, phycocyanin, lutein, and β‐carotene, which exhibit significant biological activities (Zhang et al., 2016). Other than food applications, microalgae are now being researched to be considered as a source for extracting valuable components such as chitin and chitosan, which are otherwise predominantly sourced from marine organisms. Carrageenan, a polysaccharide extracted from microalgae, can also be used to replace marine gelatin for gel strength applications (Fauzi et al., 2021). Considering the sustainability point of view as well, microalgal propagation and utilization outperform many seafood resources. According to Schade et al. (2020), considering the ecological impact yardsticks such as greenhouse gas emissions, acidification potential, eutrophication potential, and water consumption, microalgae are superiorly efficient to aquacultured seafood options. All these factors indicates that considering the emerging trend of eco‐sensitive food choices, microalgae hold huge potential as an alternative source of nutrition compared to seafood.

## 5 FLAVOR AND TEXTURE FEATURES OF SEAFOOD

Flavor and texture are two of the most important attributes of food, which have a direct correlation with consumer acceptance. It is sensory traits such as mouth feel, touch, and smell in specific combinations that create distinct characteristics in different food items, enabling consumers to make differentiations among the categories and differentiate as plant‐ or animal‐based. Seafood is no different in this aspect and possesses some of the most unique and diverse texture, color, and flavor properties in the food world. The high concentration of moisture and the differences in the quality and quantity of connective tissue make seafood significantly different in texture properties when compared to terrestrial meat. Seafood muscles in general have a higher water‐holding capacity compared to other meats (Petricorena, 2015). The sense perceived by a consumer when he/she tastes and smells food simultaneously is often termed as flavor, which is also highly significant in the case of seafood. The seafood is reported to have an array of taste elements, which include amino acids like glutamate, alanine, glycine, and arginine, and nucleotides like inosine monophosphate (IMP), guanosine monophosphate (GMP), and adenosine monophosphate (AMP) (Duppeti et al., 2022). The varying concentrations of such elements, depending on the seafood variety, harvesting location and methods, handling and storage conditions, cooking method, etc., determine the flavor perception during the consumption of the item (Wu et al., 2022). In the case of the development of seafood alternative protein‐based products, mimicking these complex textural and flavor attributes is the key to their acceptance by consumers. That may be the reason why most of the alternate protein companies do not attempt to replicate the construct and texture of seafood but concentrate more on sensory compatibility based on form and flavor (Onwezen et al., 2021). A successful adaptation of seafood texture and flavor into the analog products requires a deeper understanding regarding the alignment and construction of seafood muscle fibers as well as the ratio of flavor components.

### 5.1 Seafood color

The external appearance of different seafood varieties is governed by genetic factors determining the presence and intensity of chromatophores and pigments such as melanin and carotenoids (Chapman & Miles, 2018). The color that is significant regarding the microalgal‐based vegan seafood analogs is the pigment responsible for tissue coloration. Different seafood tissues display different colorations according to the type of pigmentation, processing methods, spoilage, etc. So, simulating a seafood muscle color depends on the type of product intended to be developed for the consumer. Tuna, one of the popular seafood varieties, is known for its red color, which is due to the presence of oxymyoglobin produced from the oxygenation of myoglobin present in the red muscle (Marrone et al., 2015). Another premium seafood variety, the salmon, is also unique in its meat coloration. The salmon meat color is provided by the presence of carotenoid pigments like astaxanthin, which are either present naturally in the wild salmon, which they acquire through the diet or administered through feed in the case of cultured varieties (Acharya, 2012). Astaxanthin is also present in shrimp, giving the meat a characteristic coloration often associated with quality, which they acquire from their diet composed of microalgae, krill, and phytoplankton (Parisenti et al., 2011). In cephalopods like squid, cuttlefish, and octopus, the skin is rich in chromatophores with melanin and xanthommatin, but the meat is devoid of any. As a result, the meat is white in color, and further color changes depend on the cooking‐induced protein modifications (Torres‐Arreola et al., 2018).

### 5.2 Seafood flavor

Taste is the number one stimulus for the plant‐based alternate seafood purchase decision by consumers (Takefuji, 2021). Seafood or any other food palatability is formed against the consumer response toward consumption frequency along with the sensory encounters associated with it, which establishes the connection between specific food preferences and routine dietary habits (Ahmed et al., 2022; Forde, 2016). Even though this is the case, a specific palatability experience could be provided to customers by combining dominant aroma compounds while mimicking a food experience. In the case of fresh seafood, the aroma compounds in the flesh are reported to be acquired through endogenous or exogenous means, where they could be absorbed from the environment and further stored subcutaneously within the lipid reserve, later resulting in fat‐derived volatile aromatic constituents like ketones and aldehydes contributing toward the flavor (Peinado et al., 2016). Seafood flavor is also classified as taste compounds (non‐volatile) like low molecular weight peptides, organic acids and bases, free amino acids, nucleotides, carbohydrates, inorganic salts, etc. (Mohammadi et al., 2021) and odor compounds (volatile) like polyunsaturated fatty acids (PUFAs) oxidation products produced by autoxidation or lipoxygenase activity such as aldehydes, alcohols, and ketones (Tao, 2015). In the case of seafood like shellfish, the aroma is contributed by sulfuric complexes such as methanethiol, dimethyl disulfide and dimethyl sulfide (DMS), which are obtained from the breakdown of sulfur‐containing compounds like taurine, methionine, and dimethyl sulfoniopropionate (DMSP) (Varlet & Fernandez, 2010). Another class of compounds leading to seafood odor is nitrogen‐containing constituents like trimethylamine (TMA), derived from the microbial deterioration of trimethylamine oxide (TMAO) (Velasquez et al., 2016). Other than these significant components, the most important characteristic of seafood flavor is umami, which is a culminative result of the presence of components such as glutamate (Glu), inosine monophosphate (IMP), aspartate (Asp), adenosine monophosphate (AMP), salt, and guanosine monophosphate (GMP) (Ruan et al., 2022). Other than that, free amino acids such as arginine (Arg), alanine (Ala), and glycine (Gly) are also attributed to the sweet flavor of seafood.

The flavor of seafood after cooking is also another important attribute when considering the flavor perception of consumers. Volatile sulfur compounds play a significant role in creating post cooked seafood flavor. According to McGorrin (2011), sulfur compounds like dimethyl sulfide, with an odor perception of stewed clam, has been found in clams and oysters; methional, with an odor perception of boiled potato, has been found in boiled clams and crustaceans; pyrrolidino‐2,4‐(Me2)dithiazine, with a roasted odor perception, has been found in boiled shellfish; 2‐acetylthiazole, with an odor perception of popcorn, has been found in boiled clam; 2‐acetyl‐2‐thiazoline, with a roasted odor perception, has been found in cooked crustaceans; and 2‐methyl‐3‐furanthiol, with an odor perception of fishy and metallic, has been found in canned tuna. It is also observed that compounds such as furans and pyrazines found in fresh seafood generate unique aroma by taking part in the Maillard reaction during heat processing (Giri et al., 2010; Liu et al., 2022). Table 2 depicts the major flavor active components present in different seafood varieties.

**TABLE 2:** Flavor active components in different seafood varieties.

Bigeye tuna (*Thunnus obesus*)	Tilapia (*Oreochromis aureus*)	White leg shrimp (*Litopenaeus vannamei*)	Squid (*Symplectoteuthis oualaniensis*)	Mussel (*Mytilus galloprovincialis*)
Octanal	Hexanal	(Z)‐3‐methyl‐1‐butene‐1‐thiol	Pentane, 2‐methyl‐	Tetrahydrofuran
*n*‐Nonanal	Nonanal	(Z)‐4‐heptenal	Pentane, 3‐methyl‐	Trans‐2‐Butenal
Heptanal	Heptanal	1‐Octen‐3‐one	Cyclohexane	3‐Methylbutanal
(E)‐2‐Hexenal (M)	Octanal	2‐Acetyl‐1‐pyrroline	Methylcyclopentane	2‐Methylbutanal
(E)‐2‐Hexenal (D)	2,3 dihydro furan	(Z)‐1,5‐octadien‐3‐one	Benzene	1‐Penten‐3ol
Hexanal (M)	2‐Pyridinepropanoic acid, alpha. ‐methyl‐. beta. ‐oxo‐, ethyl ester	Acetic acid	Myrcene	Heptane
Hexanal (D)	Hexanedioic acid, bis(2‐ethylhexyl) ester	3‐(Methylthio)propanal	Ethylbenzene	Pentanal
Butanal	1‐Octen‐3‐ol	Linalool	Undecane	2‐Ethyl‐furan
Acetone	2,6‐bis (1,1‐dimethylethyl)‐4‐methyl‐phenol	2‐methylpropanoic acid	Octadecane	3‐Hydroxy‐2‐butanone
1‐Octen‐3‐Ol	1‐Hexanol	(E, Z)‐2,6‐nonadienal	(−)‐Limonene	Pyridine
1‐Heptanol	1‐Heptanol	2‐Acetylpyridine	Pentadecane	2‐Methoxy‐furan
1‐Hexanol (M)	1‐Nonanol	Butanoic acid	Hexadecane	Pyrrole
1‐Hexanol (D)	Ethyl benzene	Phenylacetaldehyde	Ethanol	Toluene
1‐Pentanol (M)	1,2 dimethyl benzene	2‐ and 3‐Methylbutanoic acid	Acetaldehyde	1‐Pentanol
1‐Pentanol (D)	Pentadecane	(E, E)‐2,4‐nonadienal	Hexanal	3‐Methyl‐2‐butenal
(E)‐2‐Pentenal (M)	Trichloromethane	3‐Methyl‐2,4‐nonanedione	Heptaldehyde	Octane
(E)‐2‐Pentenal (D)	Methoxy phenyl oxime	Pentanoic acid	Octanal	Hexanal
1‐Butanol	5‐(Phenoxy) methyl‐2‐amino‐1,3,4‐thiadiazoles	(E, Z)‐2,4‐decadienal	Nonanal	3‐Pentanol
1‐Propanol		2‐Acetyl‐2‐thiazoline	Decanal	2‐Hydroxy‐3‐pentanone
2‐Methyl‐1‐ Propanol		(E, E)‐2,4‐decadienal	Isovaleraldehyde	2‐4‐Dimethyl‐heptane
Ethanol		Geosmine	2‐Butanone	Furfural
2‐Heptanone		2‐methoxyphenol	Acetone	2‐4‐Dimethyl‐1‐heptene
3‐Pentanone (M)		(E, Z,E)‐2,4,6‐nonatrienal	2,3‐Pentanedione	2‐Methyl‐1H‐pyrrole
3‐Pentanone (D)		(E, E,Z)‐2,4,6‐nonatrienal	Nonanoic acid	2‐Hexenal
Propanal		Benzothiazole	Octanoic acid	2‐Furanmethanol (furfuryl alcohol)
Ethyl Acetate (M)		trans‐4,5‐Epoxy‐(E)‐2‐decenal	Acetic acid glacial	4‐Methyl‐octane
Ethyl Acetate (D)		4‐hydroxy‐2,5‐dimethyl‐3(2H)‐furanone	Ethyl acetate	p‐Xylene
2‐Butanone		(E)‐3‐heptenoic acid	Trimethyl amine	Styrene
Ethyl Butanoate		4‐methylphenol	Carbamic acid, monoammonium salt	4‐Heptanone
Propyl Acetate (M)		γ‐Decalactone	2,6‐Dimethyl pyrazine	trans‐4‐Heptenal
Propyl Acetate (D)		3‐Hydroxy‐4,5‐dimethyl‐2(5H)‐furanone		1‐(2‐Furanyl)‐ethanone
Acetoin (M)		2‐Aminoacetophenone		2‐3‐Dimethyl‐pyrazine
Acetoin (D)		Decanoic acid		5‐Methyl‐2‐furancarboxaldehyde
		5‐Ethyl‐3‐hydroxy‐4‐methyl‐2(5H)‐furanone		Benzaldehyde
		Indole		2‐thiophenecarboxal‐dehyde
		3‐Methylindole		2‐Pentyl furan
		2‐Phenylacetic acid		2‐Octanone
		4‐Hydroxy‐3‐methoxybenzaldehyde		2‐Ethyl‐6‐methyl‐pyrazine
				Octanal
				trans, trans‐2,4‐Heptadienal
				Phenylacetaldehyde
				4,7‐Dimethyl‐undecane
				4‐Methoxy‐2,5‐dimethyl‐3(2H)‐furanone
				1,2,4‐Trithiolane
				2‐Nonanone
				Nonanal
				2,6,6‐Trimethyl‐2‐cyclohexene‐1,4‐dione
				cis, trans‐2,6‐Nonadienal
				Decanal
				Heptadecane
				2,6,10,14‐Tetramethyl pentadecane
				cis‐1‐Ooctadecene
				1‐Nonadecene
				Octadecanal
				Octadecanoic acid ethyl ester
Pan et al. (2019)	Phermthong et al. (2021)	Mall and Schieberle (2016)	Huang et al. (2018)	Giogios et al. (2013)

#### 5.2.1 Odor active compounds

Understanding the composition of odor‐active volatile compounds is significant in designing a seafood identical food product, as consumer acceptance and choices are dependent on it (Ma et al., 2020). Humans identify odor by inhaling air through the nasal cavity, which in turn encounters the olfactory epithelium, where the air components interact with the mucus‐laden receptor cells signaling the brain (Williams & Ringsdorf, 2020). The odor perception occurs primarily as the understanding of the smell characteristics and secondarily by the intensity of the odor molecule (Keller & Vosshall, 2016). The raw smell of the seafood is attributed to volatile components like alcohols, aldehydes, hydrocarbons, and ketones, which are produced through the decomposition of PUFA components (Undeland, 2016). Odor active value (OAV) is a unit that is used to assess the volatile components that influence the total odor perception, which helps to understand the role of each of the odor components toward the overall odor profile of a food material as it takes into consideration both the concentration and the odor threshold of a compound present in the food material (Wu, Sun, & He, 2014; Zhou et al., 2016). Aldehydes are an important category of odor volatiles present in every variety of meat, with lower thresholds when compared to ketones and alcohols. The odor‐active compounds among the aldehydes were observed in different seafood varieties and were attributed to being created by the oxidation of fat (Vidal et al., 2016). Ketones are another category of odor volatiles present in raw seafood generated by microbial action, fat oxidation, and degradation of amino acids. Saturated and unsaturated volatile alcohols are also one of the odor‐influencing components in seafood, where the latter is reported to have a lower threshold value than the former (Wang, Zhang, et al., 2018). Many miscellaneous volatile components, such as 2‐pentyl‐furan and 2‐ethyl‐furan, were also reported from seafood (Ma et al., 2020), which are observed to originate from the oxidation of *n*‐3 and *n*‐6 PUFA (Vidal et al., 2016). Some of the volatile components with the highest OAVs were trimethylamine (TMA), which registers a rotten fish/ammonia odor perception; butanoic acid, which registers a rancid butter/cheesy odor perception; 2‐methyl butanoic acid, which registers a sweet/cheese/rancid odor perception; acetone, which registers a light ethereal/nauseating odor perception; acetic acid, which registers a vinegar/acidic/cheesy odor perception; and 3‐methyl‐1‐butanol, which registers a rancid/pungent/balsamic odor perception (Yimdee & Wang, 2016).

#### 5.2.2 Taste active compounds

Odor and taste go hand in hand in determining the consumer acceptability of food products. Other than the basic tastes like sweet, sour, salty, and bitter, seafood is particularly known for its umami and kokumi taste characteristics, which make them highly desirable to consumers, generating savoryness, continuity, density, and palatability (Zhang et al., 2012). Researchers have attributed taste‐active compounds such as 5′‐nucleotides, organic bases, free amino acids, organic acids, flavor peptides, and inorganic ions to the distinctive taste of raw and cooked seafood (Yu et al., 2017). The contributors to the umami taste were determined to be potassium, sodium, alanine, arginine, adenosine‐5′‐monophosphate (AMP), inosine‐5′‐monophosphate (IMP), glycine, and guanosine‐5′‐monophosphate (GMP) (Zhang, Ayed, et al., 2019). These compounds are also reported to have compatible or non‐compatible interactions that alter a particular taste during the sensory process. For example, a 1:1 ratio of 5′ nucleotide and L‐glutamic acid has been reported to produce umami taste to a fold of seven times compared to glutamate alone, as well as Na^+^ and Cl^−^ ions from salt also has been reported to increase the intensity of umami taste (Wang, Zhou, & Liu, 2020). Amino acids are an important group of taste‐active compounds that directly contribute to primary tastes such as sour (aspartic acid and glutamic acid), bitter (leucine, isoleucine, tyrosine, and valine), and sweet (proline, alanine, lysine, glycine, serine, and threonine) (Hakimi et al., 2022).

### 5.3 Seafood texture

The texture of biological tissues is determined by the amount of moisture and fat they contain, the alignment of the muscle fibers, the availability of sufficient quantity and quality of connective tissue, etc. General seafood tissue is observed to hold around 70%–80% of moisture, 15%–20% of proteins, and 2%–5% of lipids, along with about 2% of carbohydrates, vitamins, and minerals (Ahmed et al., 2022). The seafood muscle is mostly composed of muscular sheets known as myotomes, linked with connective tissue known as mycommata (Cheng et al., 2014). Myotomes are composed of muscle fiber constituents called myosin and actin filaments, which form myofibrils with rotating anisotropic and isotropic forms with a diameter of 5 μm, playing a crucial role in muscle contractions (Konno, 2017). The connective tissue holding these muscle components, called mycommata, is made up of collagen protein, comprising about 3%–10% of the total seafood proteins that determine the texture of seafood tissue (Aussanasuwannakul et al., 2012).

Texture is a critical quality parameter required for the wholesomeness and acceptability of seafood by the consumers, which is in turn crucial for seafood alternative products that intend to imitate the seafood texture. Seafood texture is governed by different factors such as myofibrillar protein and collagen, which facilitate resistance (tensile strength) (Wang et al., 2022), and by deteriorative processes such as microbial degradation and autolysis, resulting in protein denaturation and further softening of the tissue. The firm texture has been reported to be more acceptable to the consumer than the soft texture, as the former is associated with freshness. The texture of seafood meat also varies based on species, age, location, growth circumstances, and tissue location in the body (Cheng et al., 2014; Wu, Gu, et al., 2014). Studies have specifically demonstrated that even in the same seafood type, like salmon fish, texture parameters vary drastically, proving the irregular distribution of seafood texture (Wu, Gu, et al., 2014).

Like taste, cooking method has also shown a significant influence on the final mouthfeel of seafood meat. Cooking has been observed to denature the myofibrillar proteins, in turn altering the texture, along with taste, odor, and color (Li et al., 2017). To understand the texture profile of seafood, data on different texture parameters is necessary for comparison with sensory scores on the same. The standard method most widely used for assessing the texture parameters is using the “Texture Analyzer,” which measures the resistance offered by the sample against the exerted force. Bourne et al. (1978) proposed the texture profile analysis (TPA) method for the objective estimation of texture responses. In the assessment, a flat‐ended cylindrical probe with a 500 N load cell was employed. Double compressions of the samples' initial height were applied using a crosshead speed of 0.1 mm/s and a trigger force of 2 N. The average values for the parameters of the TPA assessment were calculated using the force‐by‐time data from each test. The hardness (N) 1 and 2 values, which represent the highest resistance to compression in the first and second phases, define cohesiveness as a ratio of Area 2/Area1's first compression's positive force area to Area 2/Area1's second compression's positive force area. Springiness (mm), which is the ratio of the force input duration during the second compression of length 2/length 1 to that during the first compression; gumminess (kgf), which is the result of cohesiveness and hardness, the force necessary to break down a semi‐solid meal for swallowing; and chewiness (kgf/mm), which is the product of hardness, springiness, and cohesiveness the labor required to chew a solid food into a condition of preparation for swallowing are attributed in the analysis to explain the texture performance. Hence, any manufacturing attempts on seafood alternate meat must meet the texture parameters achieved through TPA of genuine seafood raw material.

**FIGURE 3:**
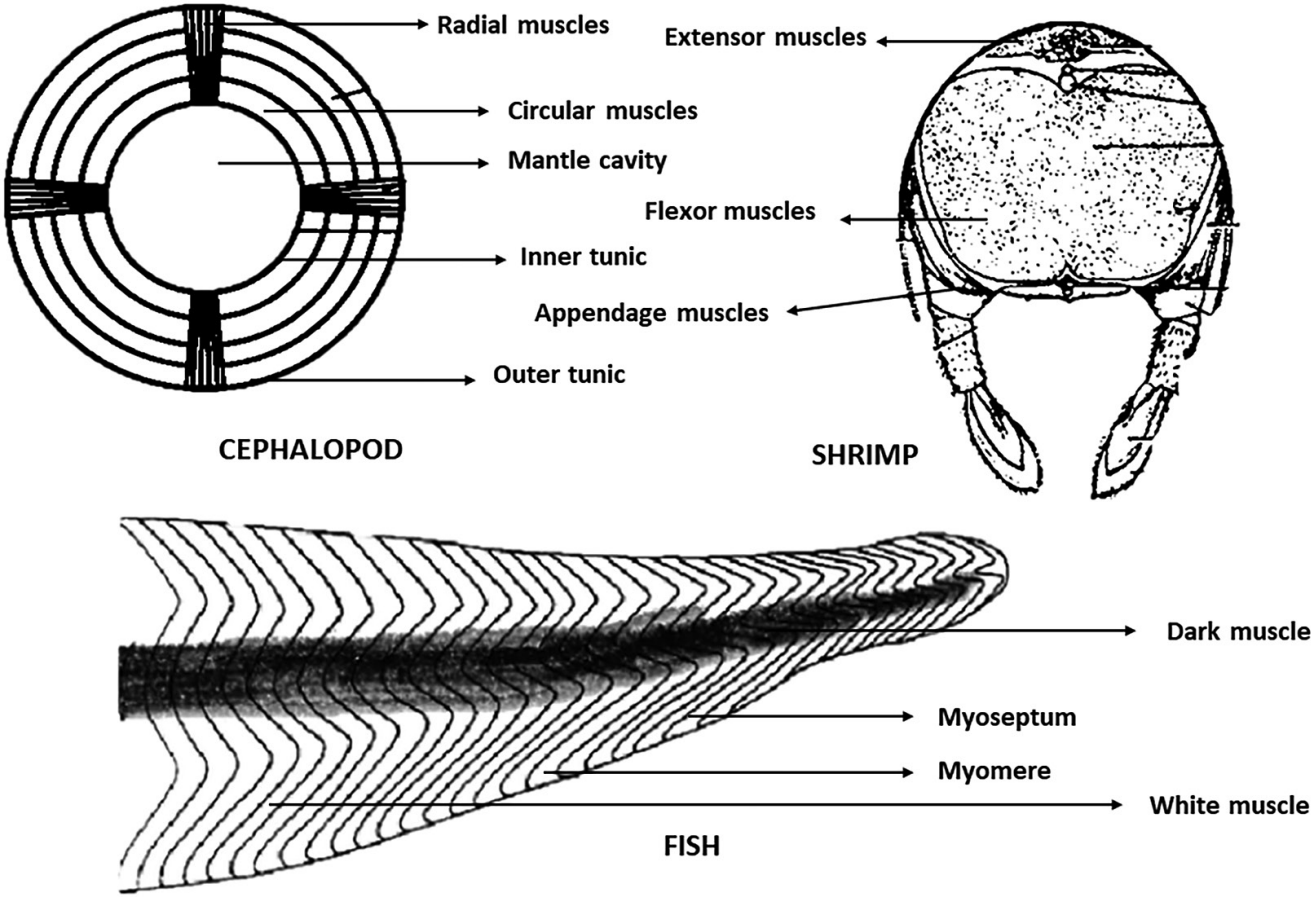
Muscle structure of different seafood varieties.

Cheng et al. (2014) describe that the texture of a seafood is influenced by structural factors like protein structure, cross‐linkage of collagen, changes in myofibrils, presence of connective tissue, muscle cellularity, muscle fiber diameter, physical factors such as seafood variety, age and size parameters, seasonal fluctuations, nutritional influence of feed, and chemical factors like distribution and quantity of moisture, distribution and quantity of fat, protein content, and collagen content. For preparing seafood identical plant‐based products, a thorough understanding of the seafood muscle structure and composition is essential. Table 3 describes the texture profile attributes of various seafood varieties, and Figure 3 depicts the structural arrangement of muscles in different seafood varieties.

**TABLE 3:** Texture profile characteristics of different seafood varieties.

Meat source	Hardness (*N*)	Cohesiveness (cm^2^)	Springiness (cm)	Gumminess (N/cm^2^)	Chewiness (N/cm)	References
Atlantic salmon (*Salmo salar*)	29.127	0.705	5.88	12.33	72.061	Wu, Sun, and He (2014)
Buefin tuna (*Thunnus maccoyii*)	523.4	0.371	0.958	305.12	310.5	Bu et al. (2022)
Tilapia (*Oreochromis aureus*)	46.2	0.29	0.6	14.2	8.57	Bilbao‐Sainz et al. (2020)
White leg shrimp (*Litopenaeus vannamei*)	8	0.21	0.34	3.99	5.35	Mehta et al. (2023)
Squid (*Symplectoteuthis oualaniensis*)	16.6	0.37	1.31	6.09	8.09	Xiao et al. (2021)
Green mussel (*Perna viridis*)	0.38	0.24	0.875	0.091	0.08	Shamsheer (2011)

**FIGURE 4:**
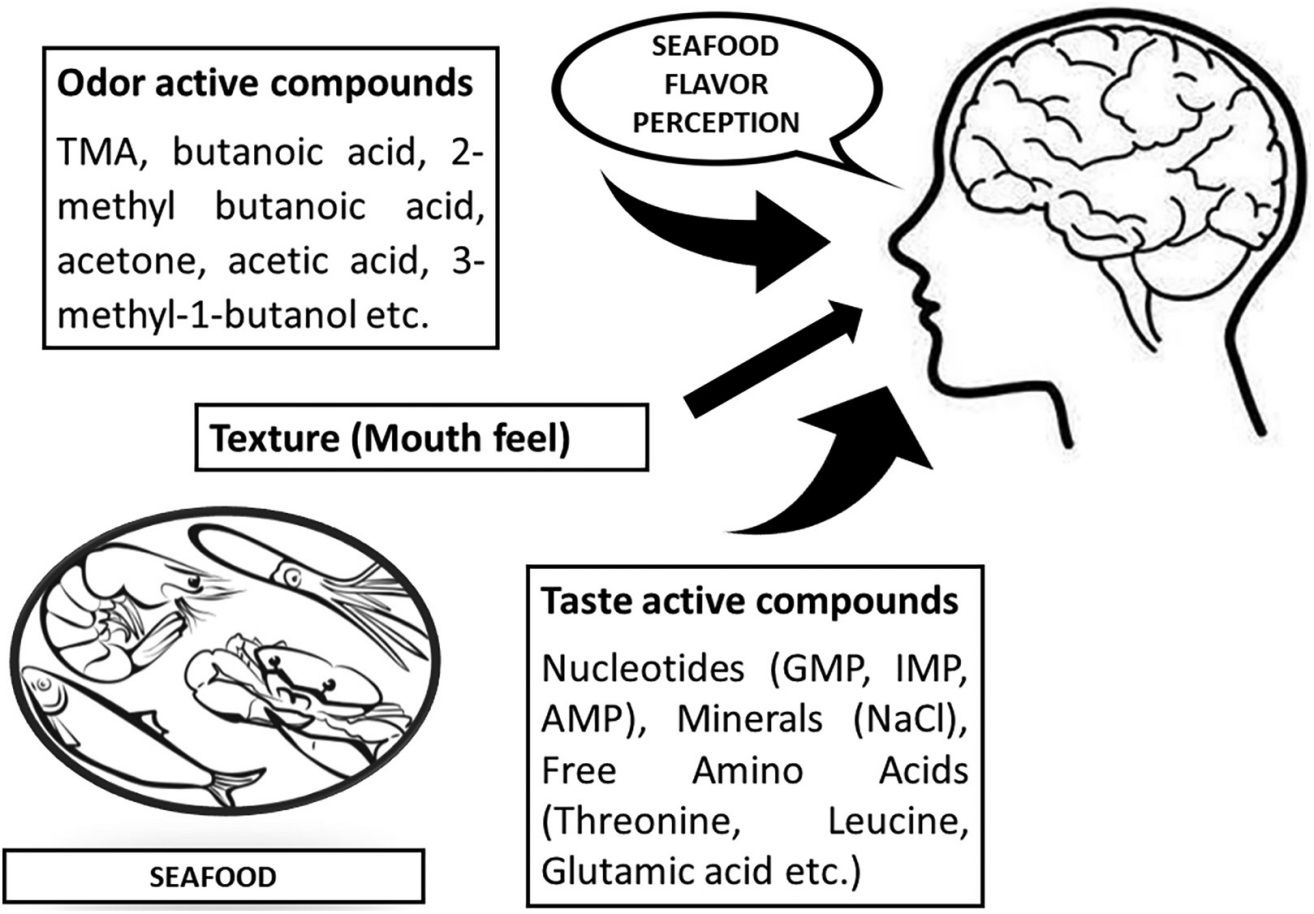
Flavor perception of seafood.

Any attempt to mimic the diverse olfactory and textural attributes of seafood must consider adapting a combination of features that is unique to each variety of seafood. The flavor components identical to seafood flavor constituents in optimum intensities could give the food a seafood‐identical flavor. The texture must be adapted by carefully constructing the muscle fiber arrangement, including the nutritional components such as fat percentage and pigmentation. Microalgae are a potential group of plants ideal to be used for the development of plant‐based seafood analogs, owing to their rich flavor including seafood identical umami (Matos et al., 2022; Zhao et al., 2024) significant availability of lipids, especially PUFA, which is also abundant in seafood and plays a major role in seafood flavor (Matos, 2016), rich source of pigments like carotenes (β‐carotene, astaxanthin, and lycopene) and xanthophylls (zeaxanthin and lutein) which could be used for creating seafood identical pigmentation in analog seafood meat (Maoka, 2020), presence of vitamins and availability of trace minerals such as sodium calcium, selenium, zinc, iron, and potassium giving health as well as taste modulations to the meat (de Farias Neves et al., 2019). Figure 4 depicts the flavor conception process of seafood by consumers.

## 6 GIVING PROPERTIES OF SEAFOOD TO ALTERNATIVE FOODS

For a plant‐based seafood alternative food product to be successful, the unique color, texture, and flavor of seafood must be simulated to the maximum so that non‐meat eaters could enjoy the seafood eating experience to its full potential. A perfect balance of these attributes, according to the seafood variety intended to mimic, is going to determine the final output of the product. A shortcoming in any of the parameter ratios may result in consumer dissatisfaction and product rejection. There are some conventional meat and seafood identical food products like tofu and tempeh, which are prepared using plant‐based ingredients such as legumes, grains, mushrooms, wheat gluten, and soybeans, and processed with an ideal amount of moisture, taste, and odor ingredients, fat, suitable binding, and color agents (Kyriakopoulou et al., 2019). The technique of simulating the muscle structure and texture of seafood consists of replicating their fiber structure even up to a nanoscale, resulting from protein chain bonding at inter‐ or intramolecular level (Borderías et al., 2020).

### 6.1 Using microalgae for seafood color simulation

To simulate natural seafood tissue coloration, pigments that could give a reddish color to the structured vegan alternative are mostly preferred. The microalgae are rich in natural pigments, which could provide seafood with identical coloration to the vegan alternative while at the same time provide additional benefits like antioxidant properties (Rodrigues et al., 2015). Phycobiliproteins, carotenoids, and chlorophylls are the major types of pigments that could be extracted from microalgae. Among which, phycobiliproteins and carotenoids could be potentially utilized for seafood identical color simulation due to their natural reddish pigmentation. Phycoerythrin, the red pigment in the class of phycobiliproteins, could be harvested from microalgae such as *Porphyridium* sp. (Bhalamurugan et al., 2018) and carotenoids like β‐carotene and Astaxanthin from *Dunaliella salina* and *Haematococcus pluvialis* (Paniagua‐Michel, 2015; Xu & Harvey, 2019).

### 6.2 Using microalgae for seafood flavor simulation

The potential of microalgae as a food source has been established owing to its palatability attributes, such as flavor, color, and texture. Different odor‐active volatile components have been isolated from algal biomass, reinforcing its aroma potential (Coleman et al., 2022). Different microalgal species, like *Tetraselmis chui*, *Phaeodactylum tricornutum*, and *Rhodomonas salina*, have been identified to possess strong seafood identical odor and taste, where the species *R. salina* and *T. chui* have been observed to hold a flavor profile identical to crabmeat (Matos et al., 2022). Microalgae being rich in protein is another factor that could result in the presence of a higher concentration of free amino acids such as glutamic acid, resulting in a higher intensity of umami taste and giving a desirable seafood identical flavor to the food products (Coleman et al., 2022). While incorporating microalgal flavor, care needs to be given to hiding the bitter taste and greasy odor for better consumer preference, which could help boost its ability in successfully simulate seafood with identical aromas (Francezon et al., 2021). Odor‐active components such as alcohols, ketones, and aldehydes have been detected in microalgae, which are formed because of the breakdown of PUFA due to autoxidation (Achyuthan et al., 2017). Microalgae like *Nannochloropsis oceanica* have been reported to contain high‐intensity grassy, fatty/fishy, and fishy/grassy odors resulting from the presence of hexanal, heptanal and 2,6‐nonadienal, 2,4‐decadienal and 4‐heptenal and 1‐octen‐3‐ol volatiles, respectively, which has been attributed to the presence of high levels of EPA and arachidonic acid (AA) content in the microalgae (Huerlimann et al., 2010). The microalgae are also rich in sulfur‐containing volatiles like dimethyl sulfide (DMS), which play an active role in the aroma characteristics of seafoods after thermal processing (Varlet & Fernandez, 2010). Odor‐active compounds such as alkyl aldehydes and benzaldehydes, which are responsible for the spoilage odor in seafood formed because of the microbial degradation of amino acids, are also reported from many microalgal species (*R. salina*, *P. tricornutum* and *T. chui*), identical to seafood‐extracted flavorings (Fu et al., 2021). Diketones like 2,3‐butanedione and 2,3‐heptanedione, responsible for the bacteria‐induced buttery smell in shrimps, have also been reported from microalgae. Fu et al. (2021) reported a higher concentration of umami taste‐contributing components in microalgae, such as free amino acids and nucleotides, and even though the microalgae are rich in seafood‐identical flavors, they also contain certain unpleasant odors, which may interfere with their application as a seafood alternative flavor additive. So, a proper flavor manipulation methodology must be adapted to deliver the most optimum aroma as per the requirement. Different odor management techniques have been suggested by many researchers, such as adding nitrogen to the microalgal growth medium, identifying the optimal harvesting time, and subjecting the microalgal biomass to thermal processing before application to nullify the unpleasant odor potential and maximize the seafood‐identical aroma components. Table 4 depicts different flavor components present in various seafood varieties in comparison to selected microalgae.

**TABLE 4:** Flavor components in selected microalgal species in comparison with various seafood varieties.

Microalgae species^a^	Salmon (*Salmo salar*)	Bigeye tuna (*Thunnus obesus*)	Tilapia (*Oreochromis aureus*)	White leg shrimp (*Litopenaeus vannamei*)	Squid (*Symplectoteuthis oualaniensis*)	Mussel (*Mytilus galloprovincialis*)
Acetic acid	1‐Penten‐3‐ol	Octanal	Hexanal	Acetic acid	Ethylbenzene	Hexanal
Octanoic acid	Hexanal	n‐Nonanal	Nonanal	(E, Z)‐2,4‐decadienal	Hexanal	2‐Hexenal
2‐Ethyl‐1‐hexanol	(E)‐2‐hexenal	(E)‐2‐Hexenal (M)	Octanal	(E, E)‐2,4‐decadienal	Octanal	Benzaldehyde
1‐Octen‐3‐ol	Benzaldehyde	(E)‐2‐Hexenal (D)	1‐Octen‐3‐ol		Nonanal	Octanal
1‐Penten‐3‐ol	1‐Octen‐3‐ol	Hexanal (M)			Decanal	trans, trans‐2,4‐Heptadienal
Benzaldehyde	Octanal	Hexanal (D)			Octanoic acid	Nonanal
Decanal	(E, E)‐2,4‐heptadienal	1‐Octen‐3‐Ol			Acetic acid glacial	Decanal
2,4‐Decadienal	2‐ethyl‐1‐hexanol					
2,4‐Heptadienal	(E)‐2‐octenal					
Hexanal	3,5‐octadien‐2‐one					
2‐Hexenal	nonanal					
Octanal	decanal					
2‐Octenal	2‐undecanone					
Nonanal	(E, Z)‐2,4‐decadienal					
Moreira et al. (2019)	Vincent Varlet et al. (2006)	Pan et al. (2022)	Phermthong et al. (2021)	Mall and Schieberle (2016)	Huang et al. (2018)	Giogios et al. (2013)

^a^ *Nannochloropsis oceanica, Tetraselmis chuii*.

### 6.3 Using microalgal components for seafood texture simulation

While reconstructing a seafood tissue structure, various aspects need to be considered. Microalgae are rich in all the necessary texture altering components like protein, ions, fat, dietary fiber, etc., which plays a crucial role in the texture performance of the seafood‐identical meat tissue. Plant‐based proteins sourced from resources such as microalgae could be used alone or in combination to achieve the desired texture. Plant proteins are frequently combined with wheat gluten to provide preferred textural properties (Yuliarti et al., 2021). The plant‐based proteins, other than the protein content, provide valuable functional properties to the seafood meat analog, like gel strength, emulsifying property, water‐holding capacity, and lipid‐binding capacity, which are necessary for a texture‐identical seafood alternative (Sha & Xiong, 2020). The globular state of plant‐based proteins aids in the formation of the typical fibrous texture of fish tissue when they are unwound, cross‐linked, and aligned accordingly (Ismail et al., 2020). This may be possible due to the physical forces in action such as van der Waals, hydrogen bonding, disulfide bonds, enzymatic, and electrostatic (Jones & McClements, 2010).

Protein solubility, gel strength, and denaturation are influenced by different salt ion concentrations that affect the texture properties when the protein‐rich components are subjected to a production process involving the application of thermal and shear forces (Tahergorabi & Jaczynski, 2012). It has been observed that long fibers of protein are formed at a lower ionic strength as it increases the solubility of proteins, depending on the distribution of charges (Jansens et al., 2019). The firmness of the tissue fibers can also be enhanced by the addition of specific salt ions. Ions of calcium and magnesium salts have been reported to increase the gel strength of soy protein (Wang et al., 2019). Ammonium sulfate was reported to enhance gel strength in fish gelatine as it facilitated the formation of a compressed hydrogel microstructure with elevated water‐holding capacity (Chen et al., 2020). Calcium ions also generated an identical effect on surimi‐based hydrogels (Zhou et al., 2019).

The presence of lipids within the protein matrix also seems to influence the structural integrity of the tissue, as the lipid particles fill the structural spaces within the gel matrix, indirectly or directly influencing the texture properties. Incorporation of lipid into surimi‐based gels has been observed to increase the physical properties of the gel, such as water‐holding capacity and breaking force (Zhou et al., 2017).

Another dietary component that could be utilized for manipulating food texture is dietary fiber. The role of dietary fibers in modifying the textural properties of seafood analog production has been previously reported (Ran et al., 2022). Dietary fibers selected according to their functional properties like water‐holding capacity, emulsifying property, and thickening ability could be incorporated into restructured seafood products for enhancing the textural properties and used as extenders, binders, or fillers in seafood analog preparation (Huang et al., 2021; Moreno et al., 2016; Yin et al., 2019).

### 6.4 Texture simulation techniques for meat analog production

**FIGURE 5:**
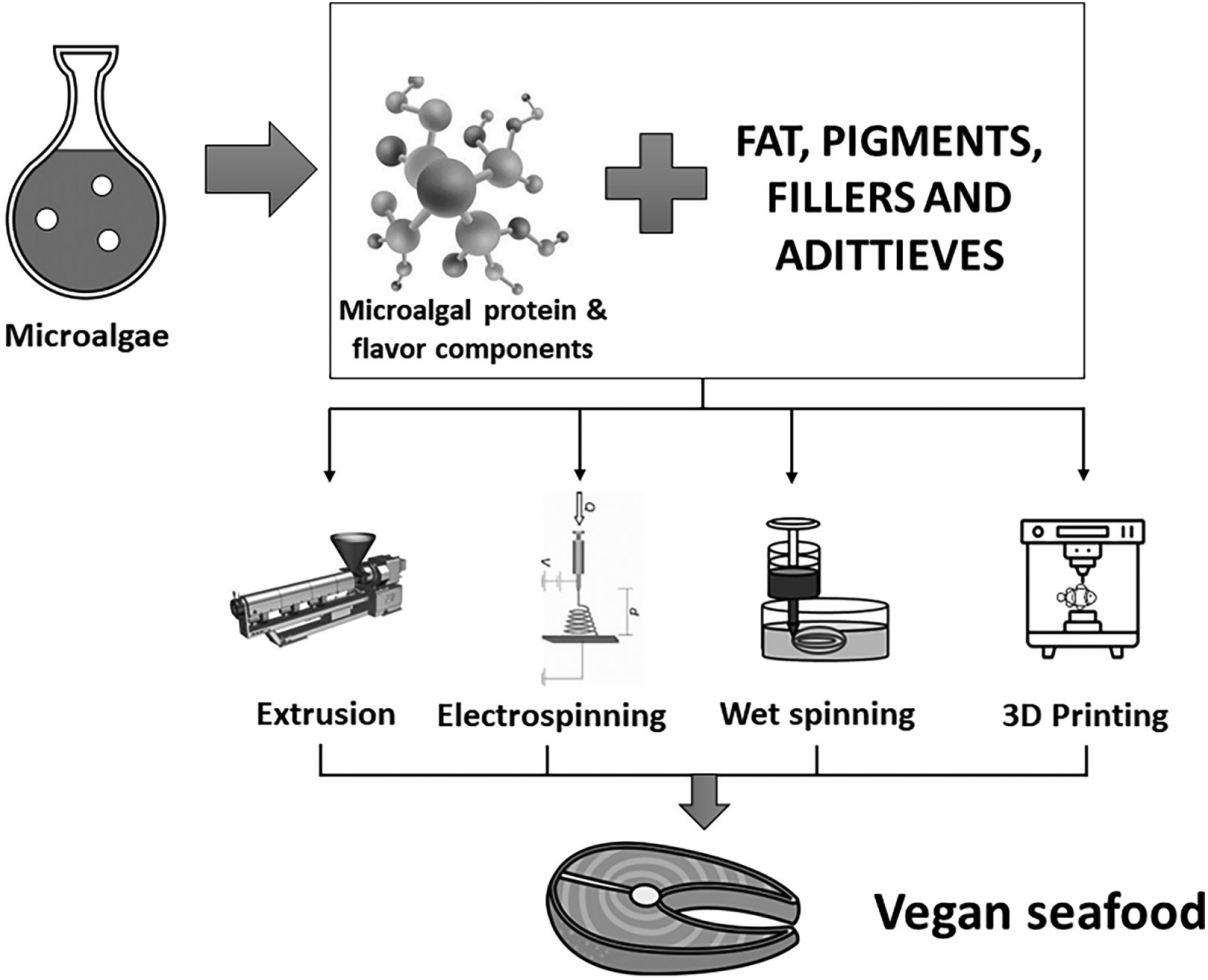
Technological process of producing microalgae‐based seafood alternative.

Different technologies are employed to produce seafood‐identical plant‐based tissue with the desired texture properties. The technology usually involves thermal assistance in combination with sheer force for modifying the texture‐altering ingredients and generating ideal characteristics during the process. The most common technology is the extrusion method (Dekkers et al., 2018); many other technologies such as electrospinning, wet spinning, and 3D printing are also employed for this purpose. Even though the direct application of microalgal biomass in texture molding is more economical, it has been observed that the thickness of the cell walls and oil content present within the biomass interfere with the texture performance, and it is advocated to utilize microalgal extracts for texture simulation (Fu et al., 2021). Figure 5 highlights the technological process of developing a seafood identical food product from microalgae.

#### 6.4.1 Extrusion method for texture simulation

Extrusion is a continuous process involving thermal and mechanical force interventions for the structural transformation of raw materials. It has been classified as low moisture extrusion (<35%) and high moisture extrusion (>50%) depending on the ware content (Caporgno et al., 2020). The high moisture extrusion method has been found advantageous as it produces, ready‐to‐eat, fibrous simulating texture, flavor, and appearance of seafood tissue (Bouvier & Campanella, 2014). The low moisture extrusion is mostly used for producing texturized vegetable protein (TVP) in dried form, which could be rehydrated to form chunks, nuggets, and crumbles that resemble minced seafood tissue (Sha & Xiong, 2020). The extrusion is performed by feeding the base material mixture containing protein, fat, and other ingredients in ideal concentration into the barrel with two rotating screws arranged in an intermeshing pattern under controlled conditions of temperature, pressure, and shear force, which are further forced through a cooling die to get the desired shape (Kyriakopoulou et al., 2019). During this process, the components in the mixture, especially protein and starch, are subjected to endothermal and physicochemical alterations at cooking temperatures in the range of 130 to 180°C and standing times of up to 150 s (Caporgno et al., 2020). The cooling die plays an important role as it provides a condition for the melted protein to cool down at a slower pace, eliminating the chance of expansion, and thereby facilitating an aligned solidification of the protein molecules, resulting in a 3D network of crosslinked layers of fibrous tissue (Wild, 2016). The output result of an extrusion process is influenced by physical parameters such as process temperature, pressure, screw speed, and die shape (Pietsch et al., 2019; Sha & Xiong, 2020), as well as nutritional parameters such as the availability of starch and protein (Zhang et al., 2021), which determine the final texture of the extrudate. Using high moisture extrusion for analogs with a higher percentage (>50%) of microalgal protein incorporation has been observed to generate some undesirable results, like unintentional texture and color properties (Grahl et al., 2018). Despite this, it was accepted in general that by keeping the moisture at minimum levels and extruding at high temperature and screw speed, anisotropic, fibrous structures could be produced from plant‐based extrusion (Grahl et al., 2018; Pietsch et al., 2019).

#### 6.4.2 Electrospinning method for texture simulation

Electrospinning technology is used to produce well‐defined, narrow‐profiled fibers at a nanoscale by putting high voltage across a polymer solution, which must be highly soluble and concentrated with optimum conductivity, surface tension, and viscosity. If these processing conditions are satisfied, the solution is drawn toward a collector, forming a Taylor cone, which spins a thread‐like structure as the solvent is slowly evaporated, leaving entangled polymer fibers (Nieuwland et al., 2014). The protein to be texturized using the electrospinning method is required to be adequately soluble and concentrated, as the spherical configuration of protein molecules could result in insufficient overlap, reducing the spinning efficiency (Dekkers et al., 2018). To resolve this issue, higher protein concentrations without compromising solubility could be applied. When using plant proteins like microalgae, as they are spherical in their natural condition, they need to be degenerated for unfolding by the application of heat before electrospinning. The electrical conductivity and viscosity of the solution are another important factor going to influence the fiber consistency. The solvent jet during the spinning process could be stabilized by increased viscosity, while a higher protein concentration could heighten the electrical conductivity (Moreira et al., 2019). An acidic solution has been reported to produce structured fibers with minimum droplet agglomeration in an experiment conducted using spirulina protein by Moreira et al. (2019). In general, plant‐based proteins could be successfully employed for producing fibrous structures in the process of forming seafood‐identical tissue using electrospinning technology.

#### 6.4.3 Wet spinning method for texture simulation

In wet spinning, the extrusion of the protein mix is performed in a bath made up of a coagulant and a solvent, which stimulates crosslinking and fiber formation by reducing the protein solubility. The solvent could be selected with protein precipitation capacity so that, along with the nozzle shear force, it could generate efficient protein alignment, resulting in elongated filaments (Dekkers et al., 2018). The solvent could also contain additives like calcium (Ca + 2) as binding agents, which creates ideal pH conditions for the formation of intermolecular and intramolecular protein bonds (Kyriakopoulou et al., 2019). It was observed that an identical protein concentration in the pre‐spun solution with that of the spinning matrix, along with a higher spinning temperature, elevated the protein mobility, resulting in lesser interactions between the molecules and preventing fiber predicament (Mu, Xu, et al., 2019).

#### 6.4.4 3D printing method for texture simulation

3D printing technology has been adopted in many food as well as non‐food applications throughout the scientific sphere (He et al., 2020). The highlight of the technology is its convenience and accuracy in printing complex designs with a variety of raw materials. The most conventional type of 3D printing technology is the syringe injection method, where the solution containing protein with high viscosity is squeezed out of a thin syringe like nozzle to form layers and eventually a pre‐determined 3D structure (Sha & Xiong, 2020). The protein solution to be used for printing must be homogenous in nature to be printable, which is decided by different factors like flow characteristics, drying rate, etc. (He et al., 2020). As the 3D‐printed seafood analogs are usually cooked after printing, the matrix must also withstand the thermal processing conditions experienced during the cooking process as well. Factors such as nozzle diameter, height, and printing speed also have an influence on the precision and integrity of the printed structure (Nachal et al., 2019). The biochemical composition of the printing material is also important when printing complex structures like seafood tissue. The presence of salts (NaCl) and combinations of starch and protein at predetermined ratios have been observed to facilitate fibrous structure formation (Phuhongsung et al., 2020).

### 6.5 Commercial outcomes for microalgal protein‐based seafood

There is no second opinion regarding the consumer popularity of seafood based on its nutritional significance and flavor diversity. Consumer preference is mostly governed by the demand–supply scenario as well as the level of awareness among the consumers regarding the respective socio‐geo‐political‐economic concerns currently being considered in recent times. There are certain awareness factors emerging regarding seafood, such as health hazards associated with contamination due to heavy metals, biotoxins, pesticide residues, microplastics, an antimicrobial resistance, and socio‐environmental issues like ecosystem degradation, violation of human rights, and overfishing (Djedjibegovic et al., 2020; Estevez et al., 2023; Hussein et al., 2022; Nakamura et al., 2022; Safina & Duckworth, 2013; Smith et al., 2018; Watts et al., 2017; Wilson et al., 2010). All these problems are being actively discussed and exposed in the modern media and are playing a crucial role in forming opinions among customers regarding rejecting or accepting seafood produce. In this scenario, the production of plant‐based alternate seafood products has opened a new niche of food product placement for customers other than vegetarians who like seafood due to its nutritional quality, flavor, and texture but are hesitant to consume it due to health and environmental concerns (Rubio et al., 2019). The food industry has responded to the new market with technologies that combine state‐of‐the‐art nutritional engineering with food design concepts. The utilization of plant proteins also provides benefits such as low health risks, safety against contaminants, and environmental sustainability when compared to natural seafood options. Microalgae are emerging as a new alternate protein source for preparing plant‐based seafood analogs owing to their nutritional similarity, seafood‐identical flavor components, adaptability to different environmentally sustainable growth conditions, and faster rate of productivity (Fu et al., 2021). Various commercial establishments, especially food start‐ups, have come up with different microalgal solutions for simulated plant‐based products. The research and development have gone into the development of efficient algal rearing systems, harvesting technologies, pigment modification, protein extraction, and purification. Regarding the texture and flavor simulation, attempts are being made to develop raw seafood tissue designs that could be utilized for catering to the sushi market and customers who prefer home cooking of their preferred seafood recipe. Other than raw seafood, attempts are also being made to develop microalgal protein‐based, value‐added seafood varieties for catering to the ready‐to‐eat market, such as smoked and dried seafood, canned seafood, and battered and breaded seafood. Advanced texture‐forming technologies are also adopted to generate seafood‐identical shapes for products such as shrimp abdomen, squid rings, and salmon fillet.

## 7 FUNCTIONAL PROPERTIES OF MICROALGAE‐BASED FOODS

### 7.1 Carbohydrates

Microalgae species such as *Porphyridium cruentum*, which contains a good amount of carbohydrates (40%–70%), and *Spirogyra* sp., which contains 33%–64% of carbohydrates, are considered for the manufacture of biofuel due to their high percentage of carbohydrates (Andreeva et al., 2021). About 50% of the dry mass of *Chlorella* sp. is made up of carbohydrates (Guccione et al., 2014). Additionally, the prebiotic effects of polysaccharides and some oligosaccharides from *Arthrospira* species, *Nostoc* species, and *Chlorella* species have been studied in the past (Eltanahy & Torky, 2021). Additionally, microalgae produce glucose or starch‐like substances, which are the key photosynthesis‐derived carbon‐containing compounds (Williams & Laurens, 2010). Glycogen storage is a known characteristic of cyanophytes, while some species also produce semi‐amylopectin (Nakamura et al., 2005). The Rhodophyta produces a carbohydrate‐like polymer regarded as floridean starch, whereas the Chlorophyta generates glucose polymers known as amylose and amylopectin (Busi et al., 2014). Chrysolaminarin is a linear polymer of 1,3‐ and 1,6‐linked glucose units that is produced by diatoms (*Bacillariophycae, Heterokontophyta*) (Gügi et al., 2015). In the exponential development phase, some diatoms can store up to 30% (dry weight) of (1,3)‐D‐glucan, and their cells can store up to 80% when there are severe nutritional restrictions (Barkia et al., 2019). About 20% of the biomass of microalgae is made up of carbohydrates, which typically accumulate as starch or other polysaccharides such as glucans, sulfated polysaccharides, and exopolysaccharides. As a result, microalgae are a source of healthy carbohydrates, and interest in using them in food applications has grown.

### 7.2 Proteins and amino acids

Microalgal cells' structure and metabolism depend heavily on proteins. Protein‐Energy Malnutrition (PEM), which is a group of clinical diseases brought on by the insufficient intake of both total energy and necessary amino acids, is one of the most significant contributors to world hunger (Scholes, 2022). A new supply of affordable, well‐balanced protein is required to address this issue. Some algae contain an extremely high proportion of protein in their dry biomass. Up to 70% of the biomass of some species, including *Arthrospira platensis*, has been estimated to be made up of proteins. The average protein level for the remaining GRAS (generally recognized as safe) species is over 40%, which is high compared to soybean (38%), pea (2.8%), rice (10%), milk (4%), and eggs (13%) (Torres‐Tiji et al., 2020). In addition to having a high protein content, algae also have a protein that is significantly richer in critical amino acids than other types of plant proteins. On a dry‐weight basis, it has been found that certain species of microalgae have very high protein concentrations. These concentrations can vary from 42% to over 70% in some cyanobacteria and 58% for *Chlorella vulgaris* (Milovanovic et al., 2012). Microalgae are superior to humans because they can generate all the necessary amino acids, which are all present in microalgae (Böcker et al., 2021). The amino acid values are also balanced and comparable to those found in great‐quality protein sources, including egg albumin, lactoglobulin, and soy (Williams & Laurens, 2010). By 2054, proteins from microalgae and insects may displace 50% of the market for proteins and peptides that are currently derived from terrestrial plants, according to Khanra et al. (2018). Considering this development, *Chlorella*, *Navicula*, *Tetraselmis*, and *Nitzschia* microalgal peptides have been used.

From *Chlorella ellipsiodea*, Ko et al. (2012) extracted a pentapeptide that contains Leu‐Asn‐Gly‐Asp‐Val‐Trp as an amino acid sequence. They then reported that the compound had notable 1,1‐Diphenyl‐2‐picrylhydrazyl (DPPH), peroxyl radical, and hydroxyl radical scavenging capacities, with IC50 values of 0.02, 0.92, and angiotensin‐converting enzyme (ACE) activity that was inhibited by two collected peptides from *Nannochloropsis oculata* showing the Gly‐Met‐Asn‐Asn‐Leu‐Thr‐Pro sequence and Leu‐Glu‐Gln sequence of amino acids, respectively. These peptides were reported to have anti‐hypertensive characteristics (Lucakova et al., 2022). Protein digestibility is an important parameter to evaluate the availability of the same for human nutrition, especially when discussing the replacement of a highly digestible protein source like seafood. Microalgal protein, depending on species and strains, has been reported to have in vivo protein digestibility of 51% to 90% in rats, mice, and humans (Acquah et al., 2020). However, the TAA (total amino acids) and EAA (essential amino acids) concentrations, as well as protein digestibility, bioaccessibility, and bioavailability, must be considered when determining whether microalgae proteins are suitable for human ingestion. When ingested, the cellulose walls of most microalgae species may prevent proper nutritional absorption. Several techniques, such as the Protein Digestibility Corrected Amino Acid Score (PDCAAS) and the Digestible Indispensable Amino Acid Score (DIAAS) approaches, are advised for evaluating the quality of proteins. The PDCAAS values, the content of protein, and the cell wall composition of various microalgae used as food, feed, and functional food. High‐quality protein sources such as eggs, soy, and whey have PDCAAS values between 0.9 and 1.0 (FAO, 2018). Unfortunately, there is currently no evidence available about the DIAAS values of microalgae or microalgae‐based protein derivatives for human meals (Wang, Tibbetts, & McGinn, 2021). However, the dried intact‐cell meal made from *Pavlova* sp. and fed to young salmon fish was recently shown to have great in vivo DIAAS values (1.0–3.6) (Tibbetts & Patelakis, 2022). The rheological properties and stability characteristics of microalgae in the production and storage stages are also influenced by proteins. These proteins are a potential source of bioactive peptides, which, when eaten, have a variety of diverse physiological benefits (Franca‐Oliveira et al., 2021). In addition, a variety of microalgae species produce enzymes with significant applications, such as enzymes with antioxidant capacities such as catalase, superoxide dismutase, and peroxidase (Roy et al., 2021). Furthermore, microalgae peptides with notable antibacterial, antioxidant, anti‐hypertensive, anti‐inflammatory, and anti‐atherosclerotic characteristics have so far been produced using enzymatic hydrolysis techniques.

### 7.3 Lipids and fatty acids

**FIGURE 6:**
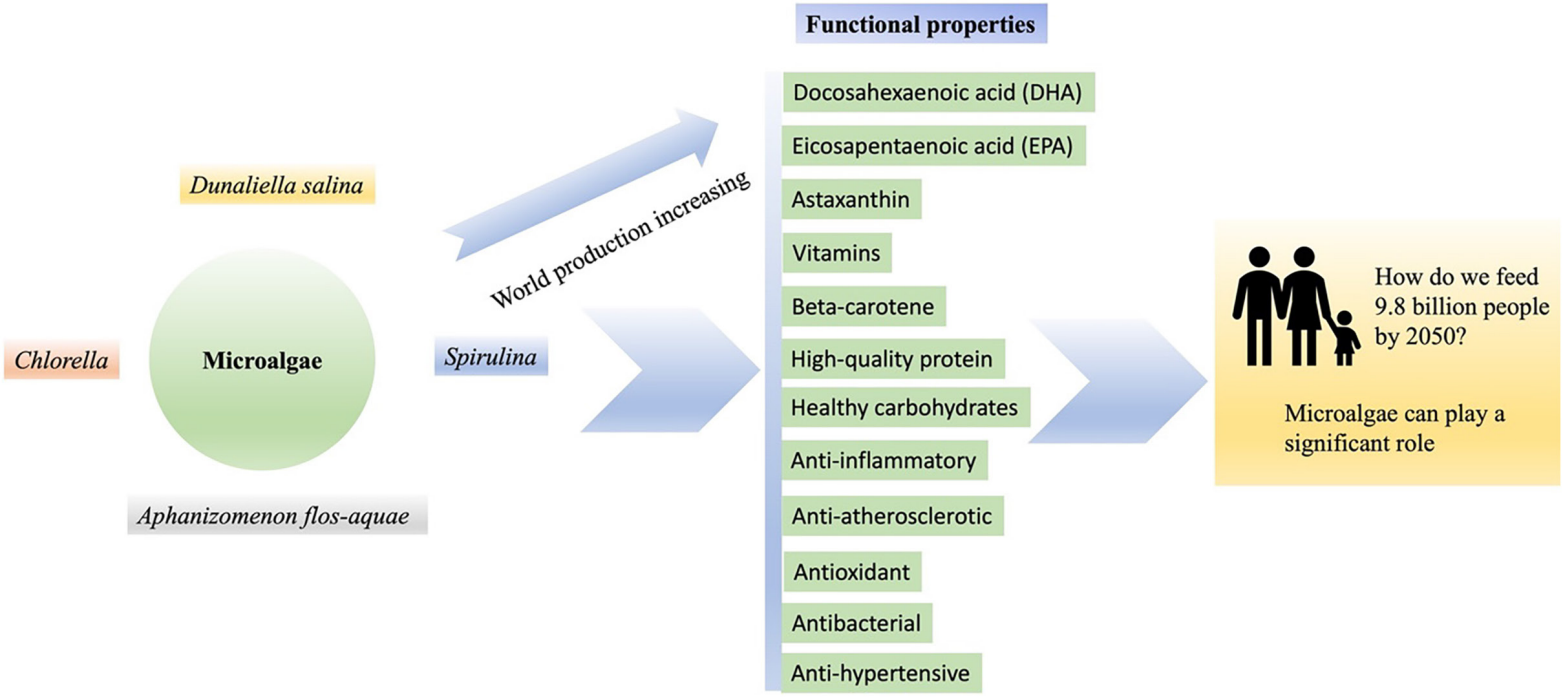
Functional properties of microalgae.

Numerous microalgal species have demonstrated a good percentage of lipid contents, and this portion can make up 20%–50% of the dry biomass (w/w) (Table 5). But additional values between 1% and 70% have also been observed (Spolaore et al., 2006). The quality and concentrations of various types of lipids are significantly influenced by cultivation circumstances, including nutrient availability, growth phase, salinity, pH, light intensity, and temperature (Perdana et al., 2021). Lipid production is dependent on the microalgal species. The creation of biodiesel was the main objective of early studies on algal lipids. However, as nutraceuticals and in infant formulas, polyunsaturated fatty acids have far more economic value (Martins et al., 2013). Because the human body is unable to synthesize polyunsaturated fatty acids (PUFAs), it must receive them from food. Omega‐3 fatty acids, such as EPA (eicosapentaenoic acid), ALA (alpha‐linolenic acid), and DHA (docosahexaenoic acid), and omega‐6 fatty acids, such as LA (linoleic acid), ARA (arachidonic acid), GLA (gamma‐linoleic acid), and CLA (conjugated linoleic acid), are separated into two types (Figure 6) (Mauro et al., 2022). It is estimated that the market value of this product is $140 USD/kg. PUFAs derived from microalgae are likewise regarded as one of the main commercial high‐value goods (Barkia et al., 2019). The popularity of microalgal fatty acids is mostly due to their minor levels of pollution than fish oils (e.g., methyl mercury, dioxins, and polychlorinated biphenyls). Algal fatty acid consumption has also demonstrated positive effects against a variety of cardiovascular illnesses, including thrombosis, myocardial infarction, hypertension, and cardiac arrhythmia (Adarme‐Vega et al., 2014). For producing DHA, several species of microalgae, such as *Schizochytrium limacinum* and *Cryptothecodininum cohnii*, have been commercially grown in fermenters (Qu et al., 2013). With an estimated $9 billion USD/year global market, fatty acid is commonly utilized as a nutritional complement in infant recipes (Barkia et al., 2019). There are several high‐value chemicals in microalgae besides carotenoids and PUFAs that have yet to be described, opening up more possibilities for the development of bioactive goods.

**TABLE 5:** Functional components of different microalgae and their products.

Species/products	Carbohydrates (% w/w)	Proteins (% w/w)	Lipids (% w/w)	Vitamins (mg/100 g w/w)	Pigments (mg/g w/w)	References
*Chlorella vulgaris*	8–28	48–57	5–21	–	–	Wang, Tibbetts, et al. (2020)
*Arthrospira platensis*	10–24	49–69	5–18	B3 (12.8) A (0.34) B9 (0.09) C (10.1)	Phycocyanin (54.65)	Kratzer and Murkovic (2021)
*Haematococcus pluvialis*	24	48	14	–	Astaxanthin (0.19–0.27)	Kratzer and Murkovic (2021)
*Dunaliella salina*	32	57	6	C (2500)	Lutein (0.04) Beta‐carotene (0.01–0.15) Zeaxanthin (6)	Kratzer and Murkovic (2021)
*Nannochloropsis oceanica*	31–39	29	18–24	D3 (0.1)	–	Lucakova et al. (2022)
*Schizochytrium* sp.	38–71	13	32			Wang, Tibbetts, et al. (2020)
*Chlorella sorkiniana*	–	50	–	A (30.77) B3 (23.8) B9 (0.09) C (10.4)	Lutein (5.21)	Wang, Tibbetts, et al. (2020)
*Nannochloropsis* sp.	9–36	28–32	14–18	–	–	Lucakova et al. (2022)
*Pavlova lutheri*	6–9	23–29	9–15			Becker (2007)
*Arthrospira platensis*‐based biscuits	22.57	35.41	16.32	C (1.25)	Carotenoids (0.02) Phycocyanin (1.35)	Abd El Baky et al. (2015)
*Spirulina platensis*‐based bread	51.09	11.63	1.36	–	–	Ak et al. (2016)
*Chlorella* sp.‐based cookies	75.2	12.2	0.3	–	–	Sahni et al. (2018)
*Spirulina platensis*‐based noodles	59.72	18.99	8.39	–	–	Kumoro et al. (2016)
*Dunaliella salina* based pasta	83.12	13.6	1.18	–	–	El‐Baz et al. (2017)

*Crypthecodinium*, *Schizochytrium*, and *Ulkenia* sp. are well‐known PUFA microalgal producers; however, additional genera like *Phaeodactylum*, *Nannochloropsis*, *Monodus*, and *Porphyridium* have also demonstrated significant quantities of EPA and DHA (Remize et al., 2021). EPA is abundant in some microalgae species, including *Nannochloropsis gaditana*, *Pavlova lutheri*, *Nannochloropsis oculata*, *Tetradesmus pseudonana*, and Phaeodactylum tricornutum, whereas docosahexaenoic acid (DHA) is abundant in others, including *Schizochytrium* sp. (DHA). *Parietochloris incisa* contains arachidonic acid, while *Arthrospira* species contain gamma‐linoleic acid and stearidonic acid (Nilsson et al., 2020). Cold‐water fish and other types of seafood have traditionally been the main sources of these elements in human diets. Fish eat algae and plankton as part of their diet, which are the ones that manufacture these necessary long‐chain PUFAS, which are abundant in these fatty acids. Some microalgae, such as *Phaeodactylum tricornutum*, can accumulate 30%–40% of all the fatty acids produced as EPA, and *Schizochytrium* sp. can gather around 50% of all the cell's total lipids as DHA (Wang, Wen, et al., 2018). To avoid some of the disadvantages of utilizing fish for these oils, including odor and non‐sustainability, algae can be a useful alternative to fish oil supplements.

### 7.4 Vitamins and minerals

In comparison to certain well‐known sources like soy flour, carrot, and orange, microalgae are a good potential supply of vitamins (Grossman, 2016). Microalgae can store retinol and carotenes, which have been shown to inhibit the growth of several cancer types (Koyande et al., 2019). These compounds can accumulate in microalgae. Recent research by Ljubic et al. (2020) looked at the buildup of vitamin D3 (cholecalciferol) up to 1 ng/g dry weight in *Nannochloropsis oceanica*, *Rhodomonas salina*, *Arthrospira maxima*, and *Chlorella minutissima* after exposure to various UVB dosages (0–36 kJ/m^2^/day) for 1 week. According to Edelmann et al. (2019), 5 g of *Nannochloropsis* and *Chlorella* microalgae powder can supply a fifth of the daily suggested intake (400 g/day) of vitamin B9. According to Tarento et al. (2018), *cyanobacteria* possess 200 g/g of vitamin K1, which is roughly six times more than the 37 g/g values recorded for parsley, a common food source of vitamin K1. Despite its limited availability in plant foods, vitamin B12 is a vital vitamin that is essential for optimum health, especially in the elderly (Amorim et al., 2021). The green alga *Dunaliella tertiolecta* is one illustration, as it has been exhibited to be a very good source of vitamins A, B9, B1, and E. B‐complex, D3, C, E, D2, and K are just a few of the water‐ and lipid‐soluble vitamins found in high concentrations in some microalgae species (Sandgruber et al., 2021). Excellent sources of vitamin D include *Tetraselmis suecica*, *Nannochloropsis oceanica*, and *Dunaliella tertiolecta*, and some microalgae, including *Dunaliella salina* and *Chlorella* sp., accumulate significant levels of vitamin C. A source of vitamin B12 was also identified as *Chlorella* sp. (Ljubic et al., 2020; Lucakova et al., 2022). Microalgae are also a rich source of minerals. Di Lena et al. (2020) report that in the microalgal biomass, sodium and calcium were found abundantly, followed by potassium, phosphorus, and magnesium. Trace minerals such as iron, manganese, and copper were also present in significant quantities.

### 7.5 Pigments

Living things mostly have purple, red, orange, and yellow colors due to carotenoids, which are lipid‐soluble pigments. Chlorophyceae, a leading carotenoid‐producing group among microalgal species, can produce just a little amount of fucoxanthin, diatoxanthin, and diadinoxanthin but up to 90% of xanthophylls and carotenes (Sui et al., 2021). According to Khanra et al. (2018), *Scenedesmus almeriensis*, *Dunaliella tertiolecta*, *Dunaliella bardawil*, and *Dunaliella salina* are the main producers of carotene. The *Chlorella* genus is said to be the greatest source of lutein, with *Chlorella protothecoides* being the leading (around 4.6% on a dry weight basis), and lutein has attracted a lot of research attention due to its food coloring substances and nutraceutical use, especially as an anticancer and antioxidant agent (Ho et al., 2014). Lutein is also extracted from several microalgal species, including *Muriellopsis* sp., *Galdieria sulphuraria*, and *Scenedesmus almeriensis*. When compared to other aquatic plants, microalgae have higher concentrations of fucoxanthin. Foods high in fucoxanthin have favorable biological effects, including antioxidant, antibacterial, anticancer, and antihypertensive properties (Chuyen & Eun, 2017). The second most utilized microalgae carotenoid after ‐carotene is astaxanthin, a red xanthophyll (Koyande et al., 2019). *Haematococcus pluvialis*, one of the microalgae species, produces the most astaxanthin, at a rate of 81% of its total carotenoid yield, which is 7% (Levasseur et al., 2020). In addition to *Haematococcus pluvialis*, a few other species, including *Chlorococcum* sp., *Chlorella zofingiensis*, and *Scenedesmus* sp., can accumulate astaxanthin, though to a much lesser extent. *Scenedesmus almeriensis* and *Nannochloropsis oculata* microalgal strains are the main producers of zeaxanthin. Zeaxanthin consumption is linked to health advantages such as defense against cancer, tumors, macular degeneration, and cataracts, according to research (Pereira et al., 2021). Algae can also provide other nutrients, such as antioxidants (including lycopene, b‐carotene, and astaxanthin) and polysaccharides (like glucans), that are beneficial to human health.

### 7.6 Functional and nutraceutical applications of microalgae

Microalgae has been identified as an excellent raw material for the development of functional foods. Some of the microalgal protein hydrolysates isolated have been reported to possess properties like gelling capacity, foaming ability, emulsifying capacity, water, and lipid absorption capabilities (Ursu et al., 2014). Functional and antioxidizing attributes of food emulsions were observed to be enhanced when microalgal components were incorporated (Caporgno & Mathys, 2018). Microalgal components could be incorporated into gels to enhance their structure and as a means to deliver antioxidants and omega‐3 fatty acids to prospective consumers (Koyande et al., 2019). Microalgal components also possess numerous nutraceutical properties beneficial for human health. Microalgae like *Spirulina* sp. have been reported to contain phycocyanin pigment, which finds its application in skin care products (Ragusa et al., 2021). Spirulina extracts are also an ingredient in immunomodulatory and anti‐inflammatory medications (Wu et al., 2016). Microalgae, such as *Chlorella* sp., contain several vital components like vitamins and minerals, as a result of which different nutraceuticals with antioxidant, immunomodulatory, antihypertensive, antihyperlipidemic, and antidiabetic activities have been derived from them, including cardiovascular medication (Bito et al., 2020).

## 8 FUTURE PROSPECTS IN MICROALGAE‐BASED FOODS

The microalgae species and their valuable co‐products are being used as ingredients in functional meals and nutraceuticals. It is accepted that products made from microalgal extracts are more valuable biologically and commercially. Notwithstanding the challenges of large‐scale production, there is a clear need for microalgal components in meals, vitamins, and possible pharmaceuticals. To fully understand the benefits and possible downsides of prospective microalgae, however, much more research is needed to describe their biochemical makeup. For their potential as antioxidants and antihypertensive agents, purified peptides and microalgal protein hydrolysates have been investigated. The identification of compounds with potential antibacterial, antifungal, antitumorigenic, and related effects, as well as the discovery of new bioactive metabolites, are further opportunities. Algae also has to go through a number of changes in order to improve growth yields, nutrient quality, organoleptic characteristics, and—most importantly—the social acceptability of algae as food. The attractiveness of algae's organoleptic characteristics may be a key to societal acceptance since it may be necessary to encourage people to consume algae products while also making them aware of the food's proven health advantages. The development of environmentally friendly, sustainable processing techniques that are efficient for transforming raw microalgal biomass into added‐value foods or isolated nutraceutical ingredients without compromising the microalgae's nutritional value or environmental impact is also required to increase the industrial use of microalgae for functional foods. Microalgal biomass may be processed sustainably and safely while retaining its nutritional content using cutting‐edge non‐thermal techniques such as microwave, fermentation, pulsed electric field, ultrasonication, and enzyme‐assisted processing. Finally, there is a need for effective teamwork between researchers and microalgal‐based enterprises to better identify industrial gaps and consumer needs, given the expanded variety of functional foods available on the market. This will aid in directing the development of research plans that will offer in‐depth information on nutritional gaps, particularly the in vivo bio‐accessibility, bioavailability, and health advantages of functional chemicals linked to the ingestion of microalgae. In any case, before commercialization, the food industry should properly address the norms and regulations controlling the quality, safety, and labeling of functional foods based on microalgae.

Another issue is the safety of microalgae for human ingestion. Heavy metal, mycotoxin, pesticide residue, and pathogen poisoning are risks linked with their ingestion. Other issues include the existence of anti‐nutritional elements, allergens, and the processing‐related change of compounds that may rise allergenicity, for instance. Future studies should concentrate on the stability of new microalgal proteins in food items as well as the breakdown and buildup of bioactive pollutants during processing. Beyond what is now permitted, inclusion in the EU list of permitted algae for use as a novel food is necessary. There are currently only a few microalgae species that have been approved, and aside from the omega‐3‐PUFA‐rich oil that is extracted from specific heterotrophic microalgae, only *Chlorella* and *Spirulina* sp. are available on the global market. These algae are primarily used for their potential as food colorants than as sources of nutrients. The Green Deal by the European Commission focuses on a variety of areas where growing and using microalgae can be very beneficial. For instance, achieving the “farm to fork” strategy for the improvement of a sustainable food system, protecting biodiversity, fostering the development of a circular economy, and becoming climate neutral by 2050 could all play a significant role in the development of microalgae for food, pharmaceuticals, and cosmetics in Europe and beyond. Microalgae may offer trustworthy and sustainable alternatives for frequently used goods with animal or plant origins, considering the expanding human population and the accessibility of terrestrial food sources. However, only a small number of strains—primarily those of the cyanobacterium *Arthrospira* and a few green microalgae (such as *Chlorella*, *Dunaliella*, and *Haematococcus*)—have been used up to this point for the industrial manufacture of a small number of goods. It will take a lot of research and development to move algal products from a niche market to being used widely as food commodities.

## 9 CONCLUSION

This review attempts to answer one of the important questions regarding 21st century food security, which is the sustainable and renewable availability of nutritional components to populations worldwide. The animal proteins, which currently dominate the current protein requirement, face multiple challenges in the form of increased carbon footprints and sustainability issues. Seafood protein, which is considered a healthy alternative to terrestrial meat, is also under scrutiny regarding overfishing, water pollution, microplastics, and intensive farming practices. Switching to plant‐based proteins is a possible solution for undressing food sustainability issues. Other than nutrition, the sensory and palate preferences of traditional meat and seafood consumers are also important factors to be considered for ensuring adaptation by the population. This article provides the comprehensive information required for such transformation regarding the potential of microalgal varieties as a raw material for producing seafood‐identical food products with identical nutritional and sensory properties. This work discusses in detail the nutritional similarity of microalgae with seafood, important species identified for the purpose, propagation and harvesting methodologies of microalgae, identification and isolation of nutritional, flavor, and color components present in microalgae identical to seafood, and manufacturing techniques for preparing seafood‐identical tissue. The work also briefly indicates the already established commercial establishments and start‐ups involved in seafood‐identical food manufacturing. The overall article provides essential information for the industry and researchers currently involved in plant‐based meat value addition.

## AUTHOR CONTRIBUTIONS

**Shahida Anusha Siddiqui:** Conceptualization (equal); investigation (equal); methodology (equal); project administration (equal); resources (equal); supervision (equal); validation (equal); visualization (equal); writing – original draft (equal); writing – review and editing (equal). **İlknur Ucak:** Writing – original draft (equal), investigation (equal); validation (equal); visualization (equal). **Maliha Afreen:** Writing – original draft (equal), investigation (equal); validation (equal); visualization (equal). **Abhilash Sasidharan:** Writing – original draft (equal); writing – review and editing (equal); investigation (equal); validation (equal). **Bello Mohammed Yunusa:** Writing – original draft (equal): investigation (equal); validation (equal); visualization (equal). **Shuva Bhowmik:** Writing – original draft (equal); investigation (equal); validation (equal); visualization (equal). **Ravi Pandiselvam:** Validation (equal). **Tigran Garrievich Ambartsumov:** Methodology (equal); investigation (equal). **Mohd Asif Shah:** Validation (equal).

## CONFLICT OF INTEREST STATEMENT

The authors declare no conflict of interest.

## Data Availability

The authors confirm that the data supporting the findings of this study are available within the article.

## References

[fsn34313-bib-0001] Abd El Baky, H. H. , El Baroty, G. S. , & Ibrahem, E. A. (2015). Functional characters evaluation of biscuits sublimated with pure phycocyanin isolated from spirulina and spirulina biomass. Nutrición Hospitalaria, 32(1), 231–241. 26262722 10.3305/nh.2015.32.1.8804

[fsn34313-bib-0002] Acharya, D. (2012). Fillet quality and yield of farmed Atlantic salmon (Salmo salar L.): Variation between families, gender differences and the importance of maturation. Norwegian University of Life Sciences.

[fsn34313-bib-0003] Achyuthan, K. E. , Harper, J. C. , Manginell, R. P. , & Moorman, M. W. (2017). Volatile metabolites emission by in vivo microalgae—An overlooked opportunity? Metabolites, 7(3), 39. 28788107 10.3390/metabo7030039 PMC5618324

[fsn34313-bib-1001] Acquah, C. , Tibbetts, S. , Pan, S. , & Udenigwe, C. (2020). Chapter 19 – Nutritional quality and bioactive properties of proteins and peptides from microalgae. In E. Jacob‐Lopes , M. M. Maroneze , M. I. Queiroz , & L. Q. Zepka (Eds.), Handbook of microalgae‐based processes and products (pp. 493–531). Academic Press. 10.1016/B978-0-12-818536-0.00019-1

[fsn34313-bib-0004] Adarme‐Vega, T. C. , Thomas‐Hall, S. R. , Lim, D. K. , & Schenk, P. M. (2014). Effects of long chain fatty acid synthesis and associated gene expression in microalga *Tetraselmis* sp. Marine Drugs, 12(6), 3381–3398. 24901700 10.3390/md12063381 PMC4071582

[fsn34313-bib-0005] Ahmed, I. , Jan, K. , Fatma, S. , & Dawood, M. A. (2022). Muscle proximate composition of various food fish species and their nutritional significance: A review. Journal of Animal Physiology and Animal Nutrition, 106(3), 690–719. 35395107 10.1111/jpn.13711

[fsn34313-bib-0006] Ak, B. , Avsaroglu, E. , Isik, O. , Özyurt, G. , Kafkas, E. , & Etyemez, M. (2016). Nutritional and physicochemical characteristics of bread enriched with microalgae Spirulina platensis. International Journal of Engineering Research and Applications, 6(9), 30–38.

[fsn34313-bib-0007] Alam, M. A. , Vandamme, D. , Chun, W. , Zhao, X. , Foubert, I. , Wang, Z. , Muylaert, K. , & Yuan, Z. (2016). Bioflocculation as an innovative harvesting strategy for microalgae. Reviews in Environmental Science and Bio/Technology, 15, 573–583.

[fsn34313-bib-0008] Amorim, M. L. , Soares, J. , Coimbra, J. S. , Leite, M. , Albino, L. F. T. , & Martins, M. A. (2021). Microalgae proteins: Production, separation, isolation, quantification, and application in food and feed. Critical Reviews in Food Science and Nutrition, 61(12), 1976–2002. 32462889 10.1080/10408398.2020.1768046

[fsn34313-bib-0009] Ananthi, V. , Raja, R. , Carvalho, I. S. , Brindhadevi, K. , Pugazhendhi, A. , & Arun, A. (2021). A realistic scenario on microalgae based biodiesel production: Third generation biofuel. Fuel, 284, 118965.

[fsn34313-bib-0010] Andrade, L. , Andrade, C. , Dias, M. , Nascimento, C. , & Mendes, M. (2018). Chlorella and spirulina microalgae as sources of functional foods. Nutraceuticals, and Food Supplements, 6(1), 45–58.

[fsn34313-bib-0011] Andreeva, A. , Budenkova, E. , Babich, O. , Sukhikh, S. , Dolganyuk, V. , Michaud, P. , & Ivanova, S. (2021). Influence of carbohydrate additives on the growth rate of microalgae biomass with an increased carbohydrate content. Marine Drugs, 19(7), 381. 34356806 10.3390/md19070381 PMC8305958

[fsn34313-bib-0012] Araújo, R. , & Peteiro, C. (2021). Algae as food and food supplements in Europe . Technical report by the Joint Research Centre (JRC), Publications Office of the European Union, 1–34.

[fsn34313-bib-0013] Atik, D. S. , Gürbüz, B. , Bölük, E. , & Palabıyık, İ. (2021). Development of vegan kefir fortified with Spirulina platensis. Food Bioscience, 42, 101050.

[fsn34313-bib-0014] Aussanasuwannakul, A. , Slider, S. D. , Salem, M. , Yao, J. , & Brett Kenney, P. (2012). Comparison of variable‐blade to Allo‐Kramer shear method in assessing rainbow trout (*Oncorhynchus mykiss*) fillet firmness. Journal of Food Science, 77(9), S335–S341. 22897606 10.1111/j.1750-3841.2012.02879.x

[fsn34313-bib-0015] Austic, R. E. , Mustafa, A. , Jung, B. , Gatrell, S. , & Lei, X. G. (2013). Potential and limitation of a new defatted diatom microalgal biomass in replacing soybean meal and corn in diets for broiler chickens. Journal of Agricultural and Food Chemistry, 61(30), 7341–7348. 23826784 10.1021/jf401957z

[fsn34313-bib-0016] Barka, A. , & Blecker, C. (2016). Microalgae as a potential source of single‐cell proteins. A review. Biotechnology, Agronomy, Society and Environment, 20, 427–436.

[fsn34313-bib-0017] Barkia, I. , Saari, N. , & Manning, S. R. (2019). Microalgae for high‐value products towards human health and nutrition. Marine Drugs, 17(5), 304. 31137657 10.3390/md17050304 PMC6562505

[fsn34313-bib-0018] Barros, A. I. , Gonçalves, A. L. , Simões, M. , & Pires, J. C. (2015). Harvesting techniques applied to microalgae: A review. Renewable and Sustainable Energy Reviews, 41, 1489–1500.

[fsn34313-bib-0019] Bartek, L. , Strid, I. , & Henryson, K. (2021). Life cycle assessment of fish oil substitute produced by microalgae using food waste. Sustainable Production and Consumption, 2021(27), 2002–2021.

[fsn34313-bib-0020] Baweja, P. , Kumar, S. , & Kumar, G. (2019). Organic fertilizer from algae: A novel approach towards sustainable agriculture. In Biofertilizers for Sustainable Agriculture and Environment (pp. 353–370). Springer.

[fsn34313-bib-0021] Becker, E. W. (2007). Micro‐algae as a source of protein. Biotechnology Advances, 25(2), 207–210. 17196357 10.1016/j.biotechadv.2006.11.002

[fsn34313-bib-0022] Behera, B. , Acharya, A. , Gargey, I. A. , Aly, N. , & Balasubramanian, P. (2019). Bioprocess engineering principles of microalgal cultivation for sustainable biofuel production. Bioresource Technology Reports, 5, 297–316.

[fsn34313-bib-0023] Benedetti, M. , Vecchi, V. , Barera, S. , & Dall'osto, L. (2018). Biomass from microalgae: The potential of domestication towards sustainable biofactories. Microbial Cell Factories, 17, 1–18. 30414618 10.1186/s12934-018-1019-3 PMC6230293

[fsn34313-bib-0024] Bernaerts, T. M. , Kyomugasho, C. , Van Looveren, N. , Gheysen, L. , Foubert, I. , Hendrickx, M. E. , & Van Loey, A. M. (2018). Molecular and rheological characterization of different cell wall fractions of *Porphyridium cruentum* . Carbohydrate Polymers, 195, 542–550. 29805010 10.1016/j.carbpol.2018.05.001

[fsn34313-bib-0025] Bhalamurugan, G. L. , Valerie, O. , & Mark, L. (2018). Valuable bioproducts obtained from microalgal biomass and their commercial applications: A review. Environmental Engineering Research, 23(3), 229–241.

[fsn34313-bib-0026] Bilbao‐Sainz, C. , Sinrod, A. J. , Williams, T. , Wood, D. , Chiou, B.‐S. , Bridges, D. F. , Wu, V. C. , Lyn, C. , Rubinsky, B. , & McHugh, T. (2020). Preservation of tilapia (*Oreochromis aureus*) fillet by isochoric (constant volume) freezing. Journal of Aquatic Food Product Technology, 29(7), 629–640.

[fsn34313-bib-1002] Bito, T. , Okumura, E. , Fujishima, M. , & Watanabe, F. (2020). Potential of chlorella as a dietary supplement to promote human health. Nutrients, 12(9), 2524. 10.3390/nu12092524 32825362 PMC7551956

[fsn34313-bib-1003] Bleakley, S. , & Hayes, M. (2017). Algal proteins: Extraction, application, and challenges concerning production. Foods, 6(5), 33. 10.3390/foods6050033 28445408 PMC5447909

[fsn34313-bib-0027] Blockx, J. , Verfaillie, A. , Thielemans, W. , & Muylaert, K. (2018). Unravelling the mechanism of chitosan‐driven flocculation of microalgae in seawater as a function of pH. ACS Sustainable Chemistry & Engineering, 6(9), 11273–11279.

[fsn34313-bib-1004] Böcker, L. , Bertsch, P. , Wenner, D. , Teixeira, S. , Bergfreund, J. , Eder, S. , Fischer, P. , & Mathys, A. (2021). Effect of *Arthrospira platensis* microalgae protein purification on emulsification mechanism and efficiency. Journal of Colloid and Interface Science, 584, 344–353. 10.1016/j.jcis.2020.09.067 33070074

[fsn34313-bib-0028] Borderías, A. J. , Tovar, C. A. , Domínguez‐Timón, F. , Díaz, M. T. , Pedrosa, M. M. , & Moreno, H. M. (2020). Characterization of healthier mixed surimi gels obtained through partial substitution of myofibrillar proteins by pea protein isolates. Food Hydrocolloids, 107, 105976.

[fsn34313-bib-0029] Boukid, F. , & Castellari, M. (2021). Food and beverages containing algae and derived ingredients launched in the market from 2015 to 2019: A front‐of‐pack labeling perspective with a special focus on Spain. Food, 10(1), 173. 10.3390/foods10010173 PMC7830845 33467009

[fsn34313-bib-0030] Boukid, F. , Rosell, C. M. , Rosene, S. , Bover‐Cid, S. , & Castellari, M. (2022). Non‐animal proteins as cutting‐edge ingredients to reformulate animal‐free foodstuffs: Present status and future perspectives. Critical Reviews in Food Science and Nutrition, 62(23), 6390–6420. 33775185 10.1080/10408398.2021.1901649

[fsn34313-bib-0031] Bourne, M. , Kenny, J. , & Barnard, J. (1978). Computer‐assisted readout of data from texture profile analysis curves 1. Journal of Texture Studies, 9(4), 481–494.

[fsn34313-bib-0032] Bouvier, J.‐M. , & Campanella, O. H. (2014). Extrusion processing technology: Food and non‐food biomaterials. John Wiley & Sons.

[fsn34313-bib-0033] Brasil, B. , Silva, F. , & Siqueira, F. (2017). Microalgae biorefineries: The Brazilian scenario in perspective. New Biotechnology, 39, 90–98. 27343427 10.1016/j.nbt.2016.04.007

[fsn34313-bib-0034] Bu, Y. , Han, M. , Tan, G. , Zhu, W. , Li, X. , & Li, J. (2022). Changes in quality characteristics of southern bluefin tuna (*Thunnus maccoyii*) during refrigerated storage and their correlation with color stability. LWT, 154, 112715.

[fsn34313-bib-0035] Busi, M. V. , Barchiesi, J. , Martín, M. , & Gomez‐Casati, D. F. (2014). Starch metabolism in green algae. Starch‐Stärke, 66(1–2), 28–40.

[fsn34313-bib-0036] Calicioglu, O. , Flammini, A. , Bracco, S. , Bellù, L. , & Sims, R. (2019). The future challenges of food and agriculture: An integrated analysis of trends and solutions. Sustainability, 11(1), 222.

[fsn34313-bib-0037] Callegari, A. , Bolognesi, S. , Cecconet, D. , & Capodaglio, A. G. (2020). Production technologies, current role, and future prospects of biofuels feedstocks: A state‐of‐the‐art review. Critical Reviews in Environmental Science and Technology, 50(4), 384–436.

[fsn34313-bib-0038] Canelli, G. , Tarnutzer, C. , Carpine, R. , Neutsch, L. , Bolten, C. J. , Dionisi, F. , & Mathys, A. (2020). Biochemical and nutritional evaluation of chlorella and Auxenochlorella biomasses relevant for food application. Frontiers in Nutrition, 7, 565996. 33117841 10.3389/fnut.2020.565996 PMC7557355

[fsn34313-bib-0039] Capek, P. , Matulová, M. , Šutovská, M. , Barboríková, J. , Molitorisová, M. , & Kazimierová, I. (2020). Chlorella vulgaris α‐L‐arabino‐α‐L‐rhamno‐α, β‐D‐galactan structure and mechanisms of its anti‐inflammatory and anti‐remodelling effects. International Journal of Biological Macromolecules, 162, 188–198. 32565301 10.1016/j.ijbiomac.2020.06.151

[fsn34313-bib-0040] Caporgno, M. , & Mathys, A. (2018). Trends in microalgae incorporation into innovative food products with potential health benefits. Frontiers in Nutrition, 5, 1–10. 30109233 10.3389/fnut.2018.00058 PMC6080594

[fsn34313-bib-0041] Caporgno, M. P. , Böcker, L. , Müssner, C. , Stirnemann, E. , Haberkorn, I. , Adelmann, H. , Handschin, S. , Windhab, E. , & Mathys, A. (2020). Extruded meat analogues based on yellow, heterotrophically cultivated Auxenochlorella protothecoides microalgae. Innovative Food Science & Emerging Technologies, 59, 102275.

[fsn34313-bib-1005] Caporgno, M. P. , Haberkorn, I. , Böcker, L. , & Mathys, A. (2019). Cultivation of Chlorella protothecoides under different growth modes and its utilisation in oil/water emulsions. Bioresource Technology, 288, 121476. 10.1016/j.biortech.2019.121476 31128535

[fsn34313-bib-0042] Cardoso, L. G. , Duarte, J. H. , Andrade, B. B. , Lemos, P. V. F. , Costa, J. A. V. , Druzian, J. I. , & Chinalia, F. A. (2020). Spirulina sp. LEB 18 cultivation in outdoor pilot scale using aquaculture wastewater: High biomass, carotenoid, lipid and carbohydrate production. Aquaculture, 525, 735272.

[fsn34313-bib-0043] Carrasco‐Reinado, R. , Escobar, A. , Carrera, C. , Guarnizo, P. , Vallejo, R. A. , & Fernández‐Acero, F. J. (2019). Valorization of microalgae biomass as a potential source of high‐value sugars and polyalcohols. LWT, 114, 108385.

[fsn34313-bib-0044] Carrasco‐Reinado, R. , Escobar‐Niño, A. , Fajardo, C. , Morano, I. M. , Amil‐Ruiz, F. , Martinez‐Rodríguez, G. , Fuentes‐Almagro, C. , Capilla, V. , Tomás‐Cobos, L. , Soriano‐Romaní, L. , Guarnizo, P. , Vallejo, R. A. , & Fernández‐Acero, F. J. (2020). Development of new antiproliferative compound against human tumor cells from the marine microalgae *Nannochloropsis gaditana* by applied proteomics. International Journal of Molecular Sciences, 22(1), 96. 33374179 10.3390/ijms22010096 PMC7795124

[fsn34313-bib-0045] Çelekli, A. , Alslibi, Z. A. , & Üseyin Bozkurt, H. (2019). Influence of incorporated Spirulina platensis on the growth of microflora and physicochemical properties of ayran as a functional food. Algal Research, 44, 101710.

[fsn34313-bib-0046] Cerón‐García, M. , González‐López, C. , Camacho‐Rodríguez, J. , López‐Rosales, L. , García‐Camacho, F. , & Molina‐Grima, E. (2018). Maximizing carotenoid extraction from microalgae used as food additives and determined by liquid chromatography (HPLC). Food Chemistry, 257, 316–324. 29622217 10.1016/j.foodchem.2018.02.154

[fsn34313-bib-0047] Chapman, F. A. , & Miles, R. D. (2018). How ornamental fish get their color: FA192, 5/2018. EDIS, 2018(3), 1–6.

[fsn34313-bib-0048] Chatzissavvidis, C. , & Therios, I. (2014). Role of algae in agriculture. In V. H. Pomin (Ed.), Seaweeds (pp. 1–37). Nova Science Publishers.

[fsn34313-bib-0049] Cheirsilp, B. , & Torpee, S. (2012). Enhanced growth and lipid production of microalgae under mixotrophic culture condition: Effect of light intensity, glucose concentration and fed‐batch cultivation. Bioresource Technology, 110, 510–516. 22361073 10.1016/j.biortech.2012.01.125

[fsn34313-bib-0050] Chen, H. , He, Z. , Zhang, B. , Feng, H. , Kandasamy, S. , & Wang, B. (2019). Effects of the aqueous phase recycling on bio‐oil yield in hydrothermal liquefaction of spirulina platensis, α‐cellulose, and lignin. Energy, 179, 1103–1113.

[fsn34313-bib-0051] Chen, H. , Shi, P. , Fan, F. , Chen, H. , Wu, C. , Xu, X. , Wang, Z. , & Du, M. (2020). Hofmeister effect‐assisted one step fabrication of fish gelatin hydrogels. LWT, 121, 108973.

[fsn34313-bib-0052] Chen, X. , Sun, D. , Zhang, X. , Liang, P. , & Huang, X. (2015). Novel self‐driven microbial nutrient recovery cell with simultaneous wastewater purification. Scientific Reports, 5(1), 1–10. 10.1038/srep15744 PMC4621542 26503712

[fsn34313-bib-0053] Cheng, J. H. , Sun, D. W. , Han, Z. , & Zeng, X. A. (2014). Texture and structure measurements and analyses for evaluation of fish and fillet freshness quality: A review. Comprehensive Reviews in Food Science and Food Safety, 13(1), 52–61. 33412693 10.1111/1541-4337.12043

[fsn34313-bib-0054] Chuyen, H. V. , & Eun, J.‐B. (2017). Marine carotenoids: Bioactivities and potential benefits to human health. Critical Reviews in Food Science and Nutrition, 57(12), 2600–2610. 26565683 10.1080/10408398.2015.1063477

[fsn34313-bib-0055] Coffey, D. , Dawson, K. , Ferket, P. , & Connolly, A. (2016). Review of the feed industry from a historical perspective and implications for its future. Journal of Applied Animal Nutrition, 4, e3.

[fsn34313-bib-0056] Coleman, B. , Van Poucke, C. , Dewitte, B. , Ruttens, A. , Moerdijk‐Poortvliet, T. , Latsos, C. , De Reu, K. , Blommaert, L. , Duquenne, B. , Timmermans, K. , van Houcke, J. , Muylaert, K. , & Robbens, J. (2022). Potential of microalgae as flavoring agents for plant‐based seafood alternatives. Future Foods, 5, 100139.

[fsn34313-bib-0057] Coppens, J. , Grunert, O. , Van Den Hende, S. , Vanhoutte, I. , Boon, N. , Haesaert, G. , & De Gelder, L. (2016). The use of microalgae as a high‐value organic slow‐release fertilizer results in tomatoes with increased carotenoid and sugar levels. Journal of Applied Phycology, 28, 2367–2377.

[fsn34313-bib-0058] Daneshvar, E. , Ok, Y. S. , Tavakoli, S. , Sarkar, B. , Shaheen, S. M. , Hong, H. , Luo, Y. , Rinklebe, J. , Song, H. , & Bhatnagar, A. (2021). Insights into upstream processing of microalgae: A review. Bioresource Technology, 329, 124870. 33652189 10.1016/j.biortech.2021.124870

[fsn34313-bib-0059] de Farias Neves, F. , Demarco, M. , & Tribuzi, G. (2019). Drying and quality of microalgal powders for human alimentation. In Microalgae‐from physiology to application. IntechOpen.

[fsn34313-bib-0060] de Jesus Raposo, M. F. , De Morais, A. M. M. B. , & De Morais, R. M. S. C. (2016). Emergent sources of prebiotics: Seaweeds and microalgae. Marine Drugs, 14(2), 27. 26828501 10.3390/md14020027 PMC4771980

[fsn34313-bib-0061] De Mendonca, H. V. , Assemany, P. , Abreu, M. , Couto, E. , Maciel, A. M. , Duarte, R. L. , dos Santos, M. G. , & Reis, A. (2021). Microalgae in a global world: New solutions for old problems? Renewable Energy, 165, 842–862.

[fsn34313-bib-1006] De Morais, G. M. , da Silva Vas, B. , de Morais, E. G. , & Costa, J. A. V. (2015). Biologically active metabolites synthesized by microalgae. BioMed Research International, 2015, 835761. 10.1155/2015/835761 26339647 PMC4538420

[fsn34313-bib-0062] Dekkers, B. L. , Boom, R. M. , & van der Goot, A. J. (2018). Structuring processes for meat analogues. Trends in Food Science & Technology, 81, 25–36.

[fsn34313-bib-1007] Di Lena, G. , Casini, I. , Lucarini, M. , Sánchez del, P. J. , Aguzzi, A. , Caproni, R. , Gabrielli, P. , & Lombardi‐Boccia, G. (2020). Chemical characterization and nutritional evaluation of microalgal biomass from large‐scale production: A comparative study of five species. European Food Research and Technology, 246. 10.1007/s00217-019-03346-5

[fsn34313-bib-1008] Djedjibegovic, J. , Marjanovic, A. , Tahirovic, D. , Caklovica, K. , Turalic, A. , Lugusic, A. , Omeragic, E. , Sober, M. , & Caklovica, F. (2020). Heavy metals in commercial fish and seafood products and risk assessment in adult population in Bosnia and Herzegovina. Scientific Reports, 10, 13238. 10.1038/s41598-020-70205-9 32764674 PMC7411038

[fsn34313-bib-1009] Duppeti, H. , Manjabhatta, S. N. , Martin, A. , & Kempaiah, B. B. (2022). Effects of different processing methods on the biochemical composition, color and non‐volatile taste active compounds of whiteleg shrimp (*Litopenaeus vannamei*). Food Chemistry Advances, 1, 100118. 10.1016/j.focha.2022.100118

[fsn34313-bib-0063] Edelmann, M. , Aalto, S. , Chamlagain, B. , Kariluoto, S. , & Piironen, V. (2019). Riboflavin, niacin, folate and vitamin B12 in commercial microalgae powders. Journal of Food Composition and Analysis, 82, 103226.

[fsn34313-bib-0064] El‐Baz, F. K. , Abdo, S. M. , & Hussein, A. M. (2017). Microalgae *Dunaliella salina* for use as food supplement to improve pasta quality. International Journal of Pharmaceutical Sciences Review and Research, 46(2), 45–51.

[fsn34313-bib-0065] El‐Naggar, N. E.‐A. , Hussein, M. H. , Shaaban‐Dessuuki, S. A. , & Dalal, S. R. (2020). Production, extraction and characterization of Chlorella vulgaris soluble polysaccharides and their applications in AgNPs biosynthesis and biostimulation of plant growth. Scientific Reports, 10(1), 3011. 32080302 10.1038/s41598-020-59945-w PMC7033187

[fsn34313-bib-0066] Eltanahy, E. , & Torky, A. (2021). Microalgae as cell factories: Food and feed‐grade high‐value metabolites, CHAPTER 1. Microalgae as Cell Factories: Food and Feed‐grade High‐value Metabolites .

[fsn34313-bib-1010] Estevez, P. , Oses Prieto, J. , Burlingame, A. , & Gago Martinez, A. (2023). Characterization of the Ciguatoxin profile in fish samples from the eastern Atlantic ocean using capillary liquid chromatography‐high resolution mass spectrometry. Food Chemistry, 418, 135960. 10.1016/j.foodchem.2023.135960 36965390

[fsn34313-bib-0067] FAO . (2018). Meeting the sustainable development goals. FAO.

[fsn34313-bib-0068] Fasaei, F. , Bitter, J. , Slegers, P. , & Van Boxtel, A. (2018). Techno‐economic evaluation of microalgae harvesting and dewatering systems. Algal Research, 31, 347–362.

[fsn34313-bib-1011] Fauzi, M. A. R. D. , Pudjiastuti, P. , Wibowo, A. C. , & Hendradi, E. (2021). Preparation, properties and potential of carrageenan‐based hard capsules for replacing gelatine: A review. Polymers, 13, 2666. 10.3390/polym13162666 34451207 PMC8400433

[fsn34313-bib-0069] Feng, X. , Walker, T. H. , Bridges, W. C. , Thornton, C. , & Gopalakrishnan, K. (2014). Biomass and lipid production of Chlorella protothecoides under heterotrophic cultivation on a mixed waste substrate of brewer fermentation and crude glycerol. Bioresource Technology, 166, 17–23. 24880808 10.1016/j.biortech.2014.03.120

[fsn34313-bib-1012] Fleurence, J. (2021). Biology and ecology of microalgae. In J. Fleurence (Ed.), Microalgae. 10.1002/9781119854944.ch1

[fsn34313-bib-0070] Fleurence, J. , & Levine, I. A. (2018). Antiallergic and allergic properties. In Microalgae in health and disease prevention (pp. 307–315). Elsevier.

[fsn34313-bib-0071] Fleurence, J. , Morançais, M. , Dumay, J. , Decottignies, P. , Turpin, V. , Munier, M. , Garcia‐Bueno, N. , & Jaouen, P. (2012). What are the prospects for using seaweed in human nutrition and for marine animals raised through aquaculture? Trends in Food Science & Technology, 27(1), 57–61.

[fsn34313-bib-0072] Forde, C. G. (2016). Flavor perception and satiation. In Flavor (pp. 251–276). Elsevier.

[fsn34313-bib-0073] Franca‐Oliveira, G. , Fornari, T. , & Hernández‐Ledesma, B. (2021). A review on the extraction and processing of natural source‐derived proteins through eco‐innovative approaches. PRO, 9(9), 1626.

[fsn34313-bib-0074] Francezon, N. , Tremblay, A. , Mouget, J.‐L. , Pasetto, P. , & Beaulieu, L. (2021). Algae as a source of natural flavors in innovative foods. Journal of Agricultural and Food Chemistry, 69(40), 11753–11772. 34597023 10.1021/acs.jafc.1c04409

[fsn34313-bib-0075] Fu, Y. , Chen, T. , Chen, S. H. Y. , Liu, B. , Sun, P. , Sun, H. , & Chen, F. (2021). The potentials and challenges of using microalgae as an ingredient to produce meat analogues. Trends in Food Science & Technology, 112, 188–200.

[fsn34313-bib-1013] García, J. L. , de Vicente, M. , & Galán, B. (2017). Microalgae, old sustainable food and fashion nutraceuticals. Microbial Biotechnology, 10(5), 1017–1024. 10.1111/1751-7915.12800 28809450 PMC5609256

[fsn34313-bib-0076] Geada, P. , Moreira, C. , Silva, M. , Nunes, R. , Madureira, L. , Rocha, C. M. , Pereira, R. N. , Vicente, A. A. , & Teixeira, J. A. (2021). Algal proteins: Production strategies and nutritional and functional properties. Bioresource Technology, 332, 125125. 33865652 10.1016/j.biortech.2021.125125

[fsn34313-bib-0077] Giogios, I. , Kalogeropoulos, N. , & Grigorakis, K. (2013). Volatile compounds of some popular Mediterranean seafood species. Mediterranean Marine Science, 14, 343–352.

[fsn34313-bib-0078] Giri, A. , Osako, K. , & Ohshima, T. (2010). Identification and characterisation of headspace volatiles of fish miso, a Japanese fish meat based fermented paste, with special emphasis on effect of fish species and meat washing. Food Chemistry, 120(2), 621–631.

[fsn34313-bib-0079] Górka, B. , Korzeniowska, K. , Lipok, J. , & Wieczorek, P. P. (2018). The biomass of algae and algal extracts in agricultural production. In Algae Biomass: Characteristics and Applications: Towards Algae‐Based Products (pp. 103–114). Springer.

[fsn34313-bib-0080] Goswami, R. K. , Agrawal, K. , & Verma, P. (2021). An overview of microalgal carotenoids: Advances in the production and its impact on sustainable development. In Bioenergy Research: Evaluating Strategies for Commercialization and Sustainability (pp. 105–128). Wiley.

[fsn34313-bib-0081] Grahl, S. , Palanisamy, M. , Strack, M. , Meier‐Dinkel, L. , Toepfl, S. , & Mörlein, D. (2018). Towards more sustainable meat alternatives: How technical parameters affect the sensory properties of extrusion products derived from soy and algae. Journal of Cleaner Production, 198, 962–971.

[fsn34313-bib-0082] Grossman, A. (2016). Nutrient acquisition: The generation of bioactive vitamin B12 by microalgae. Current Biology, 26(8), R319–R321. 27115686 10.1016/j.cub.2016.02.047

[fsn34313-bib-0083] Grossmann, L. , Hinrichs, J. , & Weiss, J. (2020). Cultivation and downstream processing of microalgae and cyanobacteria to generate protein‐based technofunctional food ingredients. Critical Reviews in Food Science and Nutrition, 60(17), 2961–2989. 31595777 10.1080/10408398.2019.1672137

[fsn34313-bib-0084] Guccione, A. , Biondi, N. , Sampietro, G. , Rodolfi, L. , Bassi, N. , & Tredici, M. R. (2014). Chlorella for protein and biofuels: From strain selection to outdoor cultivation in a Green Wall panel photobioreactor. Biotechnology for Biofuels, 7(1), 1–12. 24932216 10.1186/1754-6834-7-84 PMC4057815

[fsn34313-bib-0085] Gügi, B. , Le Costaouec, T. , Burel, C. , Lerouge, P. , Helbert, W. , & Bardor, M. (2015). Diatom‐specific oligosaccharide and polysaccharide structures help to unravel biosynthetic capabilities in diatoms. Marine Drugs, 13(9), 5993–6018. 26393622 10.3390/md13095993 PMC4584364

[fsn34313-bib-0086] Hakimi, S. , Kari, N. , Ismail, N. , Ismail, M. , & Ahmad, F. (2022). Evaluation of taste active peptides and amino acids from anchovy proteins in fish sauce by in silico approach. Food Science and Biotechnology, 31(7), 767–785. 35720460 10.1007/s10068-022-01097-w PMC9203624

[fsn34313-bib-0087] Halaj, M. , Paulovičová, E. , Paulovičová, L. , Jantová, S. , Cepák, V. , Lukavský, J. , & Capek, P. (2019). Extracellular biopolymers produced by Dictyosphaerium family‐chemical and immunomodulative properties. International Journal of Biological Macromolecules, 121, 1254–1263. 30342124 10.1016/j.ijbiomac.2018.10.116

[fsn34313-bib-0088] Hamed, I. (2016). The evolution and versatility of microalgal biotechnology: A review. Comprehensive Reviews in Food Science and Food Safety, 15(6), 1104–1123. 33401835 10.1111/1541-4337.12227

[fsn34313-bib-0089] He, C. , Zhang, M. , & Fang, Z. (2020). 3D printing of food: Pretreatment and post‐treatment of materials. Critical Reviews in Food Science and Nutrition, 60(14), 2379–2392. 31313590 10.1080/10408398.2019.1641065

[fsn34313-bib-0090] Hijazi, R. , Mounsef, J. R. , & Kanaan, H. Y. (2020). Design Considerations for Photo‐Bioreactors: A Review . Paper presented at the 2020 5th International Conference on Renewable Energies for Developing Countries (REDEC).

[fsn34313-bib-0091] Ho, S.‐H. , Chan, M.‐C. , Liu, C.‐C. , Chen, C.‐Y. , Lee, W.‐L. , Lee, D.‐J. , & Chang, J.‐S. (2014). Enhancing lutein productivity of an indigenous microalga Scenedesmus obliquus FSP‐3 using light‐related strategies. Bioresource Technology, 152, 275–282. 24296122 10.1016/j.biortech.2013.11.031

[fsn34313-bib-0092] Hodar, A. , Vasava, R. , Mahavadiya, D. , & Joshi, N. (2020). Fish meal and fish oil replacement for aqua feed formulation by using alternative sources: A review. Journal of Experimental Zoology‐India, 23(1), 13–21.

[fsn34313-bib-0093] Huang, J. , Ye, B. , Wang, W. , Li, J. , Yi, S. , Li, X. , Xu, Y. , & Mi, H. (2021). Incorporation effect of inulin and microbial transglutaminase on the gel properties of silver carp (*Hypophthalmichthys molitrix*) surimi. Journal of Food Measurement and Characterization, 15, 1–11.

[fsn34313-bib-0094] Huang, L. , Wu, Z. , Chen, X. , Weng, P. , & Zhang, X. (2018). Characterization of flavour and volatile compounds of fermented squid using electronic nose and HPMS in combination with GC‐MS. International Journal of Food Properties, 21(1), 760–770.

[fsn34313-bib-0095] Huerlimann, R. , De Nys, R. , & Heimann, K. (2010). Growth, lipid content, productivity, and fatty acid composition of tropical microalgae for scale‐up production. Biotechnology and Bioengineering, 107(2), 245–257. 20506156 10.1002/bit.22809

[fsn34313-bib-1014] Hussein, M. A. , Hammad, O. S. , Tharwat, A. E. , Darwish, W. S. , Sayed‐Ahmed, A. , Zigo, F. , Farkašová, Z. , & Rehan, I. F. (2022). Health risk assessment of organochlorine pesticide residues in edible tissue of seafood. Frontiers in Veterinary Science, 9, 1042956. 10.3389/fvets.2022.1042956 36544552 PMC9761600

[fsn34313-bib-0096] Ismail, I. , Hwang, Y.‐H. , & Joo, S.‐T. (2020). Meat analog as future food: A review. Journal of Animal Science and Technology, 62(2), 111. 32292920 10.5187/jast.2020.62.2.111 PMC7142285

[fsn34313-bib-0097] Jansens, K. J. , Rombouts, I. , Grootaert, C. , Brijs, K. , Van Camp, J. , Van der Meeren, P. , Rousseau, F. , Schymkowitz, J. , & Delcour, J. A. (2019). Rational design of amyloid‐like fibrillary structures for tailoring food protein techno‐functionality and their potential health implications. Comprehensive Reviews in Food Science and Food Safety, 18(1), 84–105. 33337021 10.1111/1541-4337.12404

[fsn34313-bib-0098] Jareonsin, S. , & Pumas, C. (2021). Advantages of heterotrophic microalgae as a host for phytochemicals production. Frontiers in Bioengineering and Biotechnology, 9, 628597. 33644020 10.3389/fbioe.2021.628597 PMC7907617

[fsn34313-bib-0099] Jones, O. G. , & McClements, D. J. (2010). Functional biopolymer particles: Design, fabrication, and applications. Comprehensive Reviews in Food Science and Food Safety, 9(4), 374–397. 33467840 10.1111/j.1541-4337.2010.00118.x

[fsn34313-bib-0100] Kaczmarczyk, M. M. , Miller, M. J. , & Freund, G. G. (2012). The health benefits of dietary fiber: Beyond the usual suspects of type 2 diabetes mellitus, cardiovascular disease and colon cancer. Metabolism, 61(8), 1058–1066. 22401879 10.1016/j.metabol.2012.01.017 PMC3399949

[fsn34313-bib-0101] Kannaujiya, V. K. , Kumar, D. , Pathak, J. , & Sinha, R. P. (2019). Phycobiliproteins and their commercial significance. In Cyanobacteria (pp. 207–216). Elsevier.

[fsn34313-bib-0102] Kassim, M. A. (2015). A study on growth, fermentation and thermochemical conversion of two microalgae species. Monash University.

[fsn34313-bib-0103] Keller, A. , & Vosshall, L. B. (2016). Olfactory perception of chemically diverse molecules. BMC Neuroscience, 17, 1–17. 27502425 10.1186/s12868-016-0287-2 PMC4977894

[fsn34313-bib-1015] Kersting, M. , Kalhoff, H. , Honermeier, B. , Sinningen, K. , & Lücke, T. (2021). Erucic acid exposure during the first year of life‐Scenarios with precise food‐based dietary guidelines. Food Science and Nutrition, 10(1), 115–121. 10.1002/fsn3.2652 35035914 PMC8751447

[fsn34313-bib-0104] Khan, M. I. , Shin, J. H. , & Kim, J. D. (2018). The promising future of microalgae: Current status, challenges, and optimization of a sustainable and renewable industry for biofuels, feed, and other products. Microbial Cell Factories, 17(1), 1–21. 29506528 10.1186/s12934-018-0879-x PMC5836383

[fsn34313-bib-0105] Khanra, S. , Mondal, M. , Halder, G. , Tiwari, O. , Gayen, K. , & Bhowmick, T. K. (2018). Downstream processing of microalgae for pigments, protein and carbohydrate in industrial application: A review. Food and Bioproducts Processing, 110, 60–84.

[fsn34313-bib-0106] Klamczynska, B. , & Mooney, W. (2017). Heterotrophic microalgae: A scalable and sustainable protein source. In Sustainable protein sources (pp. 327–339). Elsevier.

[fsn34313-bib-0107] Ko, S.‐C. , Kang, N. , Kim, E.‐A. , Kang, M. C. , Lee, S.‐H. , Kang, S.‐M. , Lee, J.‐B. , Jeon, B.‐T. , Kim, S.‐K. , Park, S. J. , Park, P.‐J. , Jung, W.‐K. , Kim, D. , & Jeon, Y.‐J. (2012). A novel angiotensin I‐converting enzyme (ACE) inhibitory peptide from a marine *Chlorella ellipsoidea* and its antihypertensive effect in spontaneously hypertensive rats. Process Biochemistry, 47(12), 2005–2011.

[fsn34313-bib-0108] Koller, M. , Muhr, A. , & Braunegg, G. (2014). Microalgae as versatile cellular factories for valued products. Algal Research, 6, 52–63.

[fsn34313-bib-0109] Konno, K. (2017). Myosin denaturation study for the quality evaluation of fish muscle‐based products. Food Science and Technology Research, 23(1), 9–21.

[fsn34313-bib-0110] Koyande, A. K. , Chew, K. W. , Rambabu, K. , Tao, Y. , Chu, D.‐T. , & Show, P.‐L. (2019). Microalgae: A potential alternative to health supplementation for humans. Food Science and Human Wellness, 8(1), 16–24.

[fsn34313-bib-0111] Kratzer, R. , & Murkovic, M. (2021). Food ingredients and nutraceuticals from microalgae: Main product classes and biotechnological production. Food, 10(7), 1626. 10.3390/foods10071626 PMC8307005 34359496

[fsn34313-bib-0112] Kumar, K. S. , Dahms, H.‐U. , Won, E.‐J. , Lee, J.‐S. , & Shin, K.‐H. (2015). Microalgae—A promising tool for heavy metal remediation. Ecotoxicology and Environmental Safety, 113, 329–352. 25528489 10.1016/j.ecoenv.2014.12.019

[fsn34313-bib-0113] Kumoro, A. , Johnny, D. , & Alfilovita, D. (2016). Incorporation of microalgae and seaweed in instant fried wheat noodles manufacturing: Nutrition and culinary properties study. International Food Research Journal, 23(2), 715–722.

[fsn34313-bib-0114] Kyriakopoulou, K. , Dekkers, B. , & van der Goot, A. J. (2019). Plant‐based meat analogues. In Sustainable meat production and processing (pp. 103–126). Elsevier.

[fsn34313-bib-0115] Laamanen, C. , Desjardins, S. , Senhorinho, G. , & Scott, J. (2021). Harvesting microalgae for health beneficial dietary supplements. Algal Research, 54, 102189.

[fsn34313-bib-0116] Laamanen, C. A. , Ross, G. M. , & Scott, J. A. (2016). Flotation harvesting of microalgae. Renewable and Sustainable Energy Reviews, 58, 75–86.

[fsn34313-bib-0117] Lafarga, T. , Sánchez‐Zurano, A. , Villaró, S. , Morillas‐España, A. , & Acién, G. (2021). Industrial production of spirulina as a protein source for bioactive peptide generation. Trends in Food Science & Technology, 116, 176–185.

[fsn34313-bib-0118] Lähteenmäki‐Uutela, A. , Rahikainen, M. , Camarena‐Gómez, M. T. , Piiparinen, J. , Spilling, K. , & Yang, B. (2021). European Union legislation on macroalgae products. Aquaculture International, 29, 487–509.

[fsn34313-bib-0119] Lamminen, M. , Halmemies‐Beauchet‐Filleau, A. , Kokkonen, T. , Jaakkola, S. , & Vanhatalo, A. (2019). Different microalgae species as a substitutive protein feed for soya bean meal in grass silage based dairy cow diets. Animal Feed Science and Technology, 247, 112–126.

[fsn34313-bib-0120] Lee, S. Y. , Khoiroh, I. , Vo, D.‐V. N. , Senthil Kumar, P. , & Show, P. L. (2021). Techniques of lipid extraction from microalgae for biofuel production: A review. Environmental Chemistry Letters, 19, 231–251.

[fsn34313-bib-0121] Levasseur, W. , Perré, P. , & Pozzobon, V. (2020). A review of high value‐added molecules production by microalgae in light of the classification. Biotechnology Advances, 41, 107545. 32272160 10.1016/j.biotechadv.2020.107545

[fsn34313-bib-0122] Levine, R. , Horst, G. , Tonda, R. , Lumpkins, B. , & Mathis, G. (2018). Evaluation of the effects of feeding dried algae containing beta‐1, 3‐glucan on broilers challenged with Eimeria. Poultry Science, 97(10), 3494–3500. 10.3382/ps/pey227 30007294

[fsn34313-bib-0123] Li, C. H. , Bland, J. M. , & Bechtel, P. J. (2017). Effect of precooking and polyphosphate treatment on the quality of microwave cooked catfish fillets. Food Science & Nutrition, 5(3), 812–819. 28572972 10.1002/fsn3.465 PMC5448386

[fsn34313-bib-0124] Liu, G. , Ito, T. , Kijima, Y. , Yoshitake, K. , Asakawa, S. , Watabe, S. , & Kinoshita, S. (2022). Zebrafish Danio rerio myotomal muscle structure and growth from a spatial transcriptomics perspective. Genomics, 114(5), 110477. 36058475 10.1016/j.ygeno.2022.110477

[fsn34313-bib-0125] Liu, X. , Zhang, M. , Liu, H. , Zhou, A. , Cao, Y. , & Liu, X. (2018). Preliminary characterization of the structure and immunostimulatory and anti‐aging properties of the polysaccharide fraction of *Haematococcus pluvialis* . RSC Advances, 8(17), 9243–9252. 35541856 10.1039/c7ra11153c PMC9078644

[fsn34313-bib-0126] Ljubic, A. , Jacobsen, C. , Holdt, S. L. , & Jakobsen, J. (2020). Microalgae Nannochloropsis oceanica as a future new natural source of vitamin D3. Food Chemistry, 320, 126627. 32213421 10.1016/j.foodchem.2020.126627

[fsn34313-bib-0127] López‐Pérez, O. , Picon, A. , & Nuñez, M. (2017). Volatile compounds and odour characteristics of seven species of dehydrated edible seaweeds. Food Research International, 99, 1002–1010. 28865610 10.1016/j.foodres.2016.12.013

[fsn34313-bib-0128] Lu, Q. , Li, H. , Zou, Y. , Liu, H. , & Yang, L. (2021). Astaxanthin as a microalgal metabolite for aquaculture: A review on the synthetic mechanisms, production techniques, and practical application. Algal Research, 54, 102178.

[fsn34313-bib-0129] Lucakova, S. , Branyikova, I. , & Hayes, M. (2022). Microalgal proteins and bioactives for food, feed, and other applications. Applied Sciences, 12(9), 4402.

[fsn34313-bib-0130] Ma, R. , Liu, X. , Tian, H. , Han, B. , Li, Y. , Tang, C. , Zhu, K. , Li, C. , & Meng, Y. (2020). Odor‐active volatile compounds profile of triploid rainbow trout with different marketable sizes. Aquaculture Reports, 17, 100312.

[fsn34313-bib-0131] Maia, M. R. , Fonseca, A. J. , Oliveira, H. M. , Mendonça, C. , & Cabrita, A. R. (2016). The potential role of seaweeds in the natural manipulation of rumen fermentation and methane production. Scientific Reports, 6(1), 32321. 27572486 10.1038/srep32321 PMC5004155

[fsn34313-bib-0132] Mall, V. , & Schieberle, P. (2016). Characterization of key aroma compounds in raw and thermally processed prawns and thermally processed lobsters by application of aroma extract dilution analysis. Journal of Agricultural and Food Chemistry, 64(33), 6433–6442. 27486834 10.1021/acs.jafc.6b02728

[fsn34313-bib-0133] Manjunath, M. , Kanchan, A. , Ranjan, K. , Venkatachalam, S. , Prasanna, R. , Ramakrishnan, B. , Hossain, F. , Nain, L. , Shivay, Y. S. , Rai, A. B. , & Singh, B. (2016). Beneficial cyanobacteria and eubacteria synergistically enhance bioavailability of soil nutrients and yield of okra. Heliyon, 2(2), e00066. 27441245 10.1016/j.heliyon.2016.e00066 PMC4945968

[fsn34313-bib-0134] Maoka, T. (2020). Carotenoids as natural functional pigments. Journal of Natural Medicines, 74(1), 1–16. 31588965 10.1007/s11418-019-01364-x PMC6949322

[fsn34313-bib-0135] Marks, E. A. , Montero, O. , & Rad, C. (2019). The biostimulating effects of viable microalgal cells applied to a calcareous soil: Increases in bacterial biomass, phosphorus scavenging, and precipitation of carbonates. Science of the Total Environment, 692, 784–790. 31539985 10.1016/j.scitotenv.2019.07.289

[fsn34313-bib-0136] Marrone, R. , Mascolo, C. , Palma, G. , Smaldone, G. , Girasole, M. , & Anastasio, A. (2015). Carbon monoxide residues in vacuum‐packed yellowfin tuna loins (*Thunnus albacares*). Italian Journal of Food Safety, 4(3), 4528. 27800404 10.4081/ijfs.2015.4528 PMC5076633

[fsn34313-bib-0137] Martins, D. A. , Custódio, L. , Barreira, L. , Pereira, H. , Ben‐Hamadou, R. , Varela, J. , & Abu‐Salah, K. M. (2013). Alternative sources of n‐3 long‐chain polyunsaturated fatty acids in marine microalgae. Marine Drugs, 11(7), 2259–2281. 23807546 10.3390/md11072259 PMC3736422

[fsn34313-bib-0138] Massironi, S. , Viganò, C. , Palermo, A. , Pirola, L. , Mulinacci, G. , Allocca, M. , Peyrin‐Biroulet, L. , & Danese, S. (2023). Inflammation and malnutrition in inflammatory bowel disease. The Lancet Gastroenterology & Hepatology, 8, 579–590. 36933563 10.1016/S2468-1253(23)00011-0

[fsn34313-bib-0139] Mathimani, T. , & Mallick, N. (2018). A comprehensive review on harvesting of microalgae for biodiesel–key challenges and future directions. Renewable and Sustainable Energy Reviews, 91, 1103–1120.

[fsn34313-bib-0140] Matos, Â. P. (2016). Essential fatty acids from microalgae. Inform, 27(10), 23–26.

[fsn34313-bib-1016] Matos, Â. P. (2017). The impact of microalgae in food science and technology. Journal of American Oil Chemist's Society, 94, 1333–1350. 10.1007/s11746-017-3050-7

[fsn34313-bib-0141] Matos, Â. P. (2019). Microalgae as a potential source of proteins. In Proteins: Sustainable source, processing and applications (pp. 63–96). Elsevier.

[fsn34313-bib-0142] Matos, Â. P. , Novelli, E. , & Tribuzi, G. (2022). Use of algae as food ingredient: Sensory acceptance and commercial products. Frontiers in Food Science and Technology, 2, 989801.

[fsn34313-bib-0143] Matter, I. A. , Bui, V. K. H. , Jung, M. , Seo, J. Y. , Kim, Y.‐E. , Lee, Y.‐C. , & Oh, Y.‐K. (2019). Flocculation harvesting techniques for microalgae: A review. Applied Sciences, 9(15), 3069.

[fsn34313-bib-0144] Mauro, A. K. , Rengarajan, A. , Albright, C. , & Boeldt, D. S. (2022). Fatty acids in normal and pathological pregnancies. Molecular and Cellular Endocrinology, 539, 111466. 34610360 10.1016/j.mce.2021.111466

[fsn34313-bib-0145] McGorrin, R. J. (2011). The significance of volatile sulfur compounds in food flavors: An overview. In Volatile Sulfur Compounds in Food (Vol. 1068, pp. 3–31). ACS Symposium Series.

[fsn34313-bib-0146] Mehta, N. K. , Rout, B. , Balange, A. K. , & Nayak, B. B. (2023). Dynamic viscoelastic behaviour, gelling properties of myofibrillar proteins and histological changes in shrimp (*L. vannamei*) muscles during ice storage. Aquaculture and Fisheries, 8(2), 180–189.

[fsn34313-bib-0147] Milledge, J. J. , & Heaven, S. (2013). A review of the harvesting of micro‐algae for biofuel production. Reviews in Environmental Science and Bio/Technology, 12, 165–178.

[fsn34313-bib-0148] Milovanovic, I. , Misan, A. , Saric, B. , Kos, J. , Mandic, A. , Simeunovic, J. , & Kovac, D. (2012). Evaluation of protein and lipid content and determination of fatty acid profile in selected species of cyanobacteria . Paper presented at the proceedings of the 6th central European congress on food, CEFood2012, Novi Sad, Serbia.

[fsn34313-bib-0149] Mohammadi, M. , Mirza Alizadeh, A. , & Mollakhalili Meybodi, N. (2021). Off‐flavors in fish: A review of potential development mechanisms, identification and prevention methods. Journal of Human Environment and Health Promotion, 7(3), 120–128.

[fsn34313-bib-0150] Moheimani, N. R. , Vadiveloo, A. , Ayre, J. M. , & Pluske, J. R. (2018). Nutritional profile and in vitro digestibility of microalgae grown in anaerobically digested piggery effluent. Algal Research, 35, 362–369.

[fsn34313-bib-0151] Moreira, J. B. , Lim, L.‐T. , da Rosa Zavareze, E. , Dias, A. R. G. , Costa, J. A. V. , & de Morais, M. G. (2019). Antioxidant ultrafine fibers developed with microalga compounds using a free surface electrospinning. Food Hydrocolloids, 93, 131–136.

[fsn34313-bib-0152] Moreno, H. M. , Herranz, B. , Pérez‐Mateos, M. , Sánchez‐Alonso, I. , & Borderías, J. A. (2016). New alternatives in seafood restructured products. Critical Reviews in Food Science and Nutrition, 56(2), 237–248. 25000341 10.1080/10408398.2012.719942

[fsn34313-bib-0153] Mouritsen, O. G. , Rhatigan, P. , & Pérez‐Lloréns, J. L. (2019). The rise of seaweed gastronomy: Phycogastronomy. Botanica Marina, 62(3), 195–209.

[fsn34313-bib-0154] Mu, B. , Xu, H. , Li, W. , Xu, L. , & Yang, Y. (2019). Spinnability and rheological properties of globular soy protein solution. Food Hydrocolloids, 90, 443–451.

[fsn34313-bib-0155] Mu, N. , Mehar, J. G. , Mudliar, S. N. , & Shekh, A. Y. (2019). Recent advances in microalgal bioactives for food, feed, and healthcare products: Commercial potential, market space, and sustainability. Comprehensive Reviews in Food Science and Food Safety, 18(6), 1882–1897. 33336956 10.1111/1541-4337.12500

[fsn34313-bib-0156] Muylaert, K. , Bastiaens, L. , Vandamme, D. , & Gouveia, L. (2017). Harvesting of microalgae: Overview of process options and their strengths and drawbacks. In Microalgae‐Based Biofuels and Bioproducts (pp. 113–132). Elsevier.

[fsn34313-bib-0157] Nachal, N. , Moses, J. , Karthik, P. , & Anandharamakrishnan, C. (2019). Applications of 3D printing in food processing. Food Engineering Reviews, 11(3), 123–141.

[fsn34313-bib-0158] Nagappan, S. , Das, P. , AbdulQuadir, M. , Thaher, M. , Khan, S. , Mahata, C. , Aljabri, H. , Vatland, A. K. , & Kumar, G. (2021). Potential of microalgae as a sustainable feed ingredient for aquaculture. Journal of Biotechnology, 341, 1–20. 34534593 10.1016/j.jbiotec.2021.09.003

[fsn34313-bib-0159] Nagarajan, D. , Varjani, S. , Lee, D.‐J. , & Chang, J.‐S. (2021). Sustainable aquaculture and animal feed from microalgae–nutritive value and techno‐functional components. Renewable and Sustainable Energy Reviews, 150, 111549.

[fsn34313-bib-1017] Nakamura, K. , Ota, Y. , & Blaha, F. (2022). A practical take on the duty to uphold human rights in seafood workplaces. Marine Policy, 135, 104844. 10.1016/j.marpol.2021.104844

[fsn34313-bib-0160] Nakamura, Y. , Takahashi, J.‐I. , Sakurai, A. , Inaba, Y. , Suzuki, E. , Nihei, S. , Fujiwara, S. , Tsuzuki, M. , Miyashita, H. , Ikemoto, H. , Kawachi, M. , Sekiguchi, H. , & Kurano, N. (2005). Some cyanobacteria synthesize semi‐amylopectin type α‐polyglucans instead of glycogen. Plant and Cell Physiology, 46(3), 539–545. 15695453 10.1093/pcp/pci045

[fsn34313-bib-0161] Neori, A. (2011). “Green water” microalgae: The leading sector in world aquaculture. Journal of Applied Phycology, 23, 143–149.

[fsn34313-bib-0162] Nguyen, T.‐T. , Binh, Q. A. , Bui, X.‐T. , Ngo, H. H. , Vo, H. N. P. , Lin, K.‐Y. A. , Vo, T. D. , Guo, W. , Lin, C. , & Breider, F. (2020). Co‐culture of microalgae‐activated sludge for wastewater treatment and biomass production: Exploring their role under different inoculation ratios. Bioresource Technology, 314, 123754. 32650264 10.1016/j.biortech.2020.123754

[fsn34313-bib-0163] Niccolai, A. , Zittelli, G. C. , Rodolfi, L. , Biondi, N. , & Tredici, M. R. (2019). Microalgae of interest as food source: Biochemical composition and digestibility. Algal Research, 42, 101617.

[fsn34313-bib-0164] Nieuwland, M. , Geerdink, P. , Brier, P. , Van Den Eijnden, P. , Henket, J. T. , Langelaan, M. L. , Stroeks, N. , van Deventer, H. , & Martin, A. H. (2014). Reprint of “food‐grade electrospinning of proteins”. Innovative Food Science & Emerging Technologies, 24, 138–144.

[fsn34313-bib-0165] Nilsson, A. K. , Jiménez, C. , & Wulff, A. (2020). Nutraceutical fatty acid production in marine microalgae and cyanobacteria. In Nutraceutical Fatty Acids from Oleaginous Microalgae: A Human Health Perspective (pp. 23–76). Wiley.

[fsn34313-bib-0166] Nurra, C. , Clavero, E. , Salvadó, J. , & Torras, C. (2014). Vibrating membrane filtration as improved technology for microalgae dewatering. Bioresource Technology, 157, 247–253. 24561630 10.1016/j.biortech.2014.01.115

[fsn34313-bib-0167] Onwezen, M. C. , Bouwman, E. P. , Reinders, M. J. , & Dagevos, H. (2021). A systematic review on consumer acceptance of alternative proteins: Pulses, algae, insects, plant‐based meat alternatives, and cultured meat. Appetite, 159, 105058. 33276014 10.1016/j.appet.2020.105058

[fsn34313-bib-0168] Pahl, S. L. , Lee, A. K. , Kalaitzidis, T. , Ashman, P. J. , Sathe, S. , & Lewis, D. M. (2013). Harvesting, thickening and dewatering microalgae biomass. In Algae for biofuels and energy (pp. 165–185). Springer Netherlands.

[fsn34313-bib-0169] Pan, S. , Jeevanandam, J. , & Danquah, M. K. (2019). Benefits of algal extracts in sustainable agriculture. In Grand Challenges in Algae Biotechnology (pp. 501–534). Springer, Cham.

[fsn34313-bib-0170] Pan, W. , Benjakul, S. , Sanmartin, C. , Guidi, A. , Ying, X. , Ma, L. , Wang, X. , Yu, J. , & Deng, S. (2022). Characterization of the flavor profile of bigeye tuna slices treated by cold plasma using E‐nose and GC‐IMS. Fishes, 7(1), 13.

[fsn34313-bib-0171] Paniagua‐Michel, J. (2015). Microalgal nutraceuticals. In Handbook of marine microalgae (pp. 255–267). Elsevier.

[fsn34313-bib-0172] Parisenti, J. , Beirão, L. H. , Tramonte, V. L. , Ourique, F. , da Silveira Brito, C. C. , & Moreira, C. C. (2011). Preference ranking of colour in raw and cooked shrimps. International Journal of Food Science & Technology, 46(12), 2558–2561.

[fsn34313-bib-0173] Parniakov, O. , Toepfl, S. , Barba, F. J. , Granato, D. , Zamuz, S. , Galvez, F. , & Lorenzo, J. M. (2018). Impact of the soy protein replacement by legumes and algae based proteins on the quality of chicken rotti. Journal of Food Science and Technology, 55, 2552–2559. 30042571 10.1007/s13197-018-3175-1 PMC6033791

[fsn34313-bib-0174] Pascoal, P. V. , Ribeiro, D. M. , Cereijo, C. R. , Santana, H. , Nascimento, R. C. , Steindorf, A. S. , Calsing, L. C. , Formighieri, E. F. , & Brasil, B. S. (2021). Biochemical and phylogenetic characterization of the wastewater tolerant Chlamydomonas biconvexa Embrapa| LBA40 strain cultivated in palm oil mill effluent. PLoS One, 16(4), e0249089. 33826653 10.1371/journal.pone.0249089 PMC8026047

[fsn34313-bib-0175] Peinado, I. , Miles, W. , & Koutsidis, G. (2016). Odour characteristics of seafood flavour formulations produced with fish by‐products incorporating EPA, DHA and fish oil. Food Chemistry, 212, 612–619. 27374575 10.1016/j.foodchem.2016.06.023

[fsn34313-bib-0176] Perdana, B. A. , Chaidir, Z. , Kusnanda, A. J. , Dharma, A. , Zakaria, I. J. , Bayu, A. , & Putra, M. Y. (2021). Omega‐3 fatty acids of microalgae as a food supplement: A review of exogenous factors for production enhancement. Algal Research, 60, 102542.

[fsn34313-bib-0177] Pereira, A. G. , Otero, P. , Echave, J. , Carreira‐Casais, A. , Chamorro, F. , Collazo, N. , Jaboui, A. , Lourenço‐Lopes, C. , Simal‐Gandara, J. , & Prieto, M. A. (2021). Xanthophylls from the sea: Algae as source of bioactive carotenoids. Marine Drugs, 19(4), 188. 33801636 10.3390/md19040188 PMC8067268

[fsn34313-bib-0178] Petricorena, Z. C. (2015). Chemical composition of fish and fishery products. In Handbook of food chemistry (pp. 403–435). Springer.

[fsn34313-bib-0179] Phermthong, P. , Worawattanamateekul, W. , & Hinsui, J. (2021). Effect of ozone treatments on Nile tilapia mince (*Oreochromis niloticus*) off‐odor. Rajamangala University of Technology Srivijaya Research Journal, 13(1), 85–94.

[fsn34313-bib-0180] Phuhongsung, P. , Zhang, M. , & Devahastin, S. (2020). Investigation on 3D printing ability of soybean protein isolate gels and correlations with their rheological and textural properties via LF‐NMR spectroscopic characteristics. LWT, 122, 109019.

[fsn34313-bib-0181] Pierre, G. , Delattre, C. , Dubessay, P. , Jubeau, S. , Vialleix, C. , Cadoret, J.‐P. , Probert, I. , & Michaud, P. (2019). What is in store for EPS microalgae in the next decade? Molecules, 24(23), 4296. 31775355 10.3390/molecules24234296 PMC6930497

[fsn34313-bib-0182] Pietsch, V. L. , Bühler, J. M. , Karbstein, H. P. , & Emin, M. A. (2019). High moisture extrusion of soy protein concentrate: Influence of thermomechanical treatment on protein‐protein interactions and rheological properties. Journal of Food Engineering, 251, 11–18.

[fsn34313-bib-0183] Prasanna, R. , Triveni, S. , Bidyarani, N. , Babu, S. , Yadav, K. , Adak, A. , Khetarpal, S. , Singh, M. P. , Shivay, Y. S. , & Saxena, A. K. (2014). Evaluating the efficacy of cyanobacterial formulations and biofilmed inoculants for leguminous crops. Archives of Agronomy and Soil Science, 60(3), 349–366.

[fsn34313-bib-0184] Priya, M. , Gurung, N. , Mukherjee, K. , & Bose, S. (2014). Microalgae in removal of heavy metal and organic pollutants from soil. In Microbial biodegradation and bioremediation (pp. 519–537). Elsevier.

[fsn34313-bib-0185] Qu, L. , Ren, L.‐J. , & Huang, H. (2013). Scale‐up of docosahexaenoic acid production in fed‐batch fermentation by *Schizochytrium* sp. based on volumetric oxygen‐transfer coefficient. Biochemical Engineering Journal, 77, 82–87.

[fsn34313-bib-1018] Ragusa, I. , Nardone, G. N. , Zanatta, S. , Bertin, W. , & Amadio, E. (2021). Spirulina for skin care: A bright blue future. Cosmetics, 8, 7. 10.3390/cosmetics8010007

[fsn34313-bib-0186] Ran, X. , Lou, X. , Zheng, H. , Gu, Q. , & Yang, H. (2022). Improving the texture and rheological qualities of a plant‐based fishball analogue by using konjac glucomannan to enhance crosslinks with soy protein. Innovative Food Science & Emerging Technologies, 75, 102910.

[fsn34313-bib-0187] Remize, M. , Brunel, Y. , Silva, J. L. , Berthon, J.‐Y. , & Filaire, E. (2021). Microalgae n‐3 PUFAs production and use in food and feed industries. Marine Drugs, 19(2), 113. 33670628 10.3390/md19020113 PMC7922858

[fsn34313-bib-0188] Renuka, N. , Guldhe, A. , Prasanna, R. , Singh, P. , & Bux, F. (2018). Microalgae as multi‐functional options in modern agriculture: Current trends, prospects and challenges. Biotechnology Advances, 36(4), 1255–1273. 29673972 10.1016/j.biotechadv.2018.04.004

[fsn34313-bib-0189] Renuka, N. , Prasanna, R. , Sood, A. , Ahluwalia, A. S. , Bansal, R. , Babu, S. , Singh, R. , Shivay, Y. S. , & Nain, L. (2016). Exploring the efficacy of wastewater‐grown microalgal biomass as a biofertilizer for wheat. Environmental Science and Pollution Research, 23, 6608–6620. 26638970 10.1007/s11356-015-5884-6

[fsn34313-bib-0190] Rodrigues, D. B. , Menezes, C. R. , Mercadante, A. Z. , Jacob‐Lopes, E. , & Zepka, L. Q. (2015). Bioactive pigments from microalgae *Phormidium autumnale* . Food Research International, 77, 273–279.

[fsn34313-bib-0191] Ronga, D. , Biazzi, E. , Parati, K. , Carminati, D. , Carminati, E. , & Tava, A. (2019). Microalgal biostimulants and biofertilisers in crop productions. Agronomy, 9(4), 192.

[fsn34313-bib-0192] Rösch, C. , Roßmann, M. , & Weickert, S. (2019). Microalgae for integrated food and fuel production. GCB Bioenergy, 11(1), 326–334.

[fsn34313-bib-0193] Roy, U. K. , Nielsen, B. V. , & Milledge, J. J. (2021). Antioxidant production in Dunaliella. Applied Sciences, 11(9), 3959.

[fsn34313-bib-0194] Ruan, L. , Ju, Y. , Zhan, C. , & Hou, L. (2022). Improved umami flavor of soy sauce by adding enzymatic hydrolysate of low‐value fish in the natural brewing process. LWT, 155, 112911.

[fsn34313-bib-0195] Rubio, N. , Datar, I. , Stachura, D. , Kaplan, D. , & Krueger, K. (2019). Cell‐based fish: A novel approach to seafood production and an opportunity for cellular agriculture. Frontiers in Sustainable Food Systems, 3, 43.

[fsn34313-bib-0196] Ruggeri, M. V. R. , Godoy, R. F. B. , Arroyo, P. A. , & Trevisan, E. (2021). Evaluation of natural flocculant efficiency in the harvest of microalgae *Monoraphidium contortum* . SN Applied Sciences, 3(6), 627.

[fsn34313-bib-0197] Rumin, J. , de Oliveira, G. , Junior, R. , Bérard, J.‐B. , & Picot, L. (2021). Improving microalgae research and marketing in the European Atlantic area: Analysis of major gaps and barriers limiting sector development. Marine Drugs, 19(6), 319. 34070907 10.3390/md19060319 PMC8229015

[fsn34313-bib-0198] Rune, C. J. B. , Song, Q. , Clausen, M. P. , & Giacalone, D. (2022). Consumer perception of plant‐based burger recipes studied by projective mapping. Future Foods, 6, 100168.

[fsn34313-bib-1019] Safina, C. , & Duckworth, A. (2013). Fish conservation. In S. A. Levin (Ed.), Encyclopedia of biodiversity (2nd ed., pp. 443–455). Academic Press. 10.1016/B978-0-12-384719-5.00315-4

[fsn34313-bib-0199] Sahni, P. , Sharma, S. , & Singh, B. (2018). Evaluation and quality assessment of defatted microalgae meal of chlorella as an alternative food ingredient in cookies. Nutrition & Food Science, 49(2), 221–231.

[fsn34313-bib-0200] Sandgruber, F. , Gielsdorf, A. , Baur, A. C. , Schenz, B. , Müller, S. M. , Schwerdtle, T. , Stangl, G. I. , Griehl, C. , Lorkowski, S. , & Dawczynski, C. (2021). Variability in macro‐and micronutrients of 15 commercially available microalgae powders. Marine Drugs, 19(6), 310. 34071995 10.3390/md19060310 PMC8228358

[fsn34313-bib-0201] Sarker, N. K. (2021). Exploring the potential of wastewater reclamation by means of outdoor cultivation of microalgae in photobioreactors. Energy, Ecology and Environment, 7, 1–16.

[fsn34313-bib-1020] Schade, S. , Stangl, G. I. , & Meier, T. (2020). Distinct microalgae species for food—part 2: Comparative life cycle assessment of microalgae and fish for eicosapentaenoic acid (EPA), docosahexaenoic acid (DHA), and protein. Journal of Applied Phycology, 32, 2997–3013. 10.1007/s10811-020-02181-6

[fsn34313-bib-0202] Schenk, P. M. (2021). Phycology: Algae for food, feed, fuel and the planet (Vol. 1, pp. 76–78). MDPI.

[fsn34313-bib-0203] Schlesinger, A. , Eisenstadt, D. , Bar‐Gil, A. , Carmely, H. , Einbinder, S. , & Gressel, J. (2012). Inexpensive non‐toxic flocculation of microalgae contradicts theories; overcoming a major hurdle to bulk algal production. Biotechnology Advances, 30(5), 1023–1030. 22306161 10.1016/j.biotechadv.2012.01.011

[fsn34313-bib-0204] Scholes, G. (2022). Protein‐energy malnutrition in older Australians: A narrative review of the prevalence, causes and consequences of malnutrition, and strategies for prevention. Health Promotion Journal of Australia, 33(1), 187–193. 33783903 10.1002/hpja.489

[fsn34313-bib-0205] Schulze, C. , Wetzel, M. , Reinhardt, J. , Schmidt, M. , Felten, L. , & Mundt, S. (2016). Screening of microalgae for primary metabolites including β‐glucans and the influence of nitrate starvation and irradiance on β‐glucan production. Journal of Applied Phycology, 28, 2719–2725.

[fsn34313-bib-0206] Sha, L. , & Xiong, Y. L. (2020). Plant protein‐based alternatives of reconstructed meat: Science, technology, and challenges. Trends in Food Science & Technology, 102, 51–61.

[fsn34313-bib-0207] Shamsheer, N. A. M. (2011). Analysis of biochemical parameters of green mussel (Perna viridis) .

[fsn34313-bib-0208] Sharma, R. , Khokhar, M. , Jat, R. , & Khandelwal, S. (2012). Role of algae and cyanobacteria in sustainable agriculture system. Wudpecker Journal of Agricultural Research, 1(9), 381–388.

[fsn34313-bib-0209] Shekh, A. , Sharma, A. , Schenk, P. M. , Kumar, G. , & Mudliar, S. (2022). Microalgae cultivation: Photobioreactors, CO_2_ utilization, and value‐added products of industrial importance. Journal of Chemical Technology & Biotechnology, 97(5), 1064–1085.

[fsn34313-bib-0210] Siddiqui, S. , Bahmid, N. A. , Mahmud, C. M. , Boukid, F. , Lamri, M. , & Gagaoua, M. (2022). Consumer acceptability of plant‐, seaweed‐, and insect‐based foods as alternatives to meat: A critical compilation of a decade of research. Critical Reviews in Food Science and Nutrition, 63(23), 6630–6651. 35144515 10.1080/10408398.2022.2036096

[fsn34313-bib-0211] Siddiqui, S. A. , Alvi, T. , Sameen, A. , Khan, S. , Blinov, A. V. , Nagdalian, A. A. , Mehdizadeh, M. , Adli, D. N. , & Onwezen, M. (2022). Consumer acceptance of alternative proteins: A systematic review of current alternative protein sources and interventions adapted to increase their acceptability. Sustainability, 14(22), 15370.

[fsn34313-bib-0212] Siddiqui, S. A. , Schulte, H. , Pleissner, D. , Schönfelder, S. , Kvangarsnes, K. , Dauksas, E. , Rustad, T. , Cropotova, J. , Heinz, V. , & Smetana, S. (2023). Transformation of seafood side‐streams and residuals into valuable products. Food, 12(2), 422. 10.3390/foods12020422 PMC9857928 36673514

[fsn34313-bib-0213] Siddiqui, S. A. , Zannou, O. , Karim, I. , Awad, N. M. , Gołaszewski, J. , Heinz, V. , & Smetana, S. (2022). Avoiding food neophobia and increasing consumer acceptance of new food trends—A decade of research. Sustainability, 14(16), 10391.

[fsn34313-bib-0214] Singh, G. , & Patidar, S. (2018). Microalgae harvesting techniques: A review. Journal of Environmental Management, 217, 499–508. 29631239 10.1016/j.jenvman.2018.04.010

[fsn34313-bib-0215] Singh, H. , Singh, J. , Singh, S. K. , Singh, N. , Paul, S. , Sohal, H. S. , Gupta, U. , & Jain, S. K. (2019). Vitamin E TPGS based palatable, oxidatively and physically stable emulsion of microalgae DHA oil for infants, children and food fortification. Journal of Dispersion Science and Technology, 41, 1–16.

[fsn34313-bib-0216] Singh, J. , & Dhar, D. W. (2019). Overview of carbon capture technology: Microalgal biorefinery concept and state‐of‐the‐art. Frontiers in Marine Science, 6, 29.

[fsn34313-bib-1021] Smith, M. , Love, D. C. , Rochman, C. M. , & Neff, R. A. (2018). Microplastics in seafood and the implications for human health. Current Environment Health Reports, 5(3), 375–386. 10.1007/s40572-018-0206-z PMC6132564 30116998

[fsn34313-bib-0217] Solimeno, A. , Acíen, F. G. , & García, J. (2017). Mechanistic model for design, analysis, operation and control of microalgae cultures: Calibration and application to tubular photobioreactors. Algal Research, 21, 236–246.

[fsn34313-bib-0218] Sousa, I. , Gouveia, L. , Batista, A. P. , Raymundo, A. , & Bandarra, N. M. (2008). Microalgae in novel food products. Food Chemistry Research Developments, 1, 75–112.

[fsn34313-bib-0219] Southey, F. (2021). Sophie's BIoNutrients: Protein made from microalgae fed with food waste comes to Europe. Food Navigator.

[fsn34313-bib-0220] Spolaore, P. , Joannis‐Cassan, C. , Duran, E. , & Isambert, A. (2006). Commercial applications of microalgae. Journal of Bioscience and Bioengineering, 101(2), 87–96. 16569602 10.1263/jbb.101.87

[fsn34313-bib-0221] Springmann, M. , Clark, M. , Mason‐D'Croz, D. , Wiebe, K. , Bodirsky, B. L. , Lassaletta, L. , de Vries, W. , Vermeulen, S. J. , Herrero, M. , Carlson, K. M. , Jonell, M. , Troell, M. , DeClerck, F. , Gordon, L. J. , Zurayk, R. , Scarborough, P. , Rayner, M. , Loken, B. , Fanzo, J. , … Willett, W. (2018). Options for keeping the food system within environmental limits. Nature, 562(7728), 519–525. 30305731 10.1038/s41586-018-0594-0

[fsn34313-bib-0222] Subhash, G. V. , Rajvanshi, M. , Kumar, B. N. , Govindachary, S. , Prasad, V. , & Dasgupta, S. (2017). Carbon streaming in microalgae: Extraction and analysis methods for high value compounds. Bioresource Technology, 244, 1304–1316. 28803061 10.1016/j.biortech.2017.07.024

[fsn34313-bib-0223] Suh, S.‐S. , Kim, S.‐M. , Kim, J. E. , Hong, J.‐M. , Lee, S. G. , Youn, U. J. , Han, S. J. , Kim, I. C. , & Kim, S. (2017). Anticancer activities of ethanol extract from the Antarctic freshwater microalga, *Botryidiopsidaceae* sp. BMC Complementary and Alternative Medicine, 17(1), 1–9. 29191192 10.1186/s12906-017-1991-x PMC5709829

[fsn34313-bib-0224] Sui, Y. , Mazzucchi, L. , Acharya, P. , Xu, Y. , Morgan, G. , & Harvey, P. J. (2021). A comparison of β‐carotene, phytoene and amino acids production in *Dunaliella salina* DF 15 (CCAP 19/41) and *Dunaliella salina* CCAP 19/30 using different light wavelengths. Food, 10(11), 2824. 10.3390/foods10112824 PMC8617983 34829102

[fsn34313-bib-0225] Suparmaniam, U. , Lam, M. K. , Uemura, Y. , Lim, J. W. , Lee, K. T. , & Shuit, S. H. (2019). Insights into the microalgae cultivation technology and harvesting process for biofuel production: A review. Renewable and Sustainable Energy Reviews, 115, 109361.

[fsn34313-bib-0226] Swarnalakshmi, K. , Prasanna, R. , Kumar, A. , Pattnaik, S. , Chakravarty, K. , Shivay, Y. S. , Singh, R. , & Saxena, A. K. (2013). Evaluating the influence of novel cyanobacterial biofilmed biofertilizers on soil fertility and plant nutrition in wheat. European Journal of Soil Biology, 55, 107–116.

[fsn34313-bib-0227] Tahergorabi, R. , & Jaczynski, J. (2012). Physicochemical changes in surimi with salt substitute. Food Chemistry, 132(3), 1281–1286. 29243612 10.1016/j.foodchem.2011.11.104

[fsn34313-bib-0228] Takefuji, Y. (2021). Sustainable protein alternatives. Trends in Food Science & Technology, 107, 429–431.

[fsn34313-bib-0229] Tao, L. (2015). Oxidation of polyunsaturated fatty acids and its impact on food quality and human health. Advances in Food Technology and Nutritional Sciences, 1, 135–142.

[fsn34313-bib-0230] Tarento, T. D. , McClure, D. D. , Vasiljevski, E. , Schindeler, A. , Dehghani, F. , & Kavanagh, J. M. (2018). Microalgae as a source of vitamin K1. Algal Research, 36, 77–87.

[fsn34313-bib-0231] Tavernari, F. D. C. , Roza, L. , Surek, D. , Sordi, C. , Silva, M. , Albino, L. , Migliorini, M. J. , Paiano, D. , & Boiago, M. (2018). Apparent metabolisable energy and amino acid digestibility of microalgae *Spirulina platensis* as an ingredient in broiler chicken diets. British Poultry Science, 59(5), 562–567. 10.1080/00071668.2018.1496401 29969915

[fsn34313-bib-0232] Tejano, L. A. , Peralta, J. P. , Yap, E. E. S. , & Chang, Y. W. (2019). Bioactivities of enzymatic protein hydrolysates derived from *Chlorella sorokiniana* . Food Science & Nutrition, 7(7), 2381–2390. 31367367 10.1002/fsn3.1097 PMC6657813

[fsn34313-bib-0233] Thangaraj, R. , Mahendran, S. , Nizhanthini, C. , Dhanasekaran, D. , & Thajuddin, N. (2023). Small/large‐scale production, cost benefit analysis, and Marketing of Spirulina Single Cell Protein. In Food microbiology based entrepreneurship: Making money from microbes (pp. 115–132). Springer.

[fsn34313-bib-0234] Tibbetts, S. M. , & Patelakis, S. J. (2022). Apparent digestibility coefficients (ADCs) of intact‐cell marine microalgae meal (*Pavlova* sp. 459) for juvenile Atlantic salmon (*Salmo salar* L.). Aquaculture, 546, 737236.

[fsn34313-bib-1022] Tiwari, K. B. , & Troy, D. J. (2015). Chapter 1 ‐ Seaweed sustainability – food and nonfood applications. In B. K. Tiwari & D. J. Troy (Eds.), Seaweed sustainability (pp. 1–6). Academic Press. 10.1016/B978-0-12-418697-2.00001-5

[fsn34313-bib-0235] Torres‐Arreola, W. , Ocaño‐Higuera, V. M. , Ezquerra‐Brauer, J. M. , López‐Corona, B. E. , Rodríguez‐Felix, F. , Castro‐Longoria, R. , & Ramírez‐Guerra, H. E. (2018). Effect of cooking on physicochemical and structural properties of jumbo squid (*Dosidicus gigas*) muscle. Journal of Food Processing and Preservation, 42(2), e13528.

[fsn34313-bib-0236] Torres‐Tiji, Y. , Fields, F. J. , & Mayfield, S. P. (2020). Microalgae as a future food source. Biotechnology Advances, 41, 107536. 32194145 10.1016/j.biotechadv.2020.107536

[fsn34313-bib-0237] Undeland, I. (2016). Oxidative stability of seafood. In Oxidative stability and shelf life of foods containing oils and fats (pp. 391–460). Elsevier.

[fsn34313-bib-1023] Ursu, A. V. , Marcati, A. , Sayd, T. , Sante‐Lhoutellier, V. , Djelveh, G. , & Michaud, P. (2014). Extraction, fractionation and functional properties of proteins from the microalgae *Chlorella vulgaris* . Bioresource Technology, 157, 134–139. 10.1016/j.biortech.2014.01.071 24534795

[fsn34313-bib-0238] Vandamme, D. , Foubert, I. , & Muylaert, K. (2013). Flocculation as a low‐cost method for harvesting microalgae for bulk biomass production. Trends in Biotechnology, 31(4), 233–239. 23336995 10.1016/j.tibtech.2012.12.005

[fsn34313-bib-0239] Varlet, V. , & Fernandez, X. (2010). Sulfur‐containing volatile compounds in seafood: Occurrence, odorant properties and mechanisms of formation. Food Science and Technology International, 16(6), 463–503. 21339165 10.1177/1082013210379688

[fsn34313-bib-0240] Varlet, V. , Knockaert, C. , Prost, C. , & Serot, T. (2006). Comparison of odor‐active volatile compounds of fresh and smoked salmon. Journal of Agricultural and Food Chemistry, 54(9), 3391–3401. 16637700 10.1021/jf053001p

[fsn34313-bib-0241] Vasistha, S. , Khanra, A. , Clifford, M. , & Rai, M. P. (2021). Current advances in microalgae harvesting and lipid extraction processes for improved biodiesel production: A review. Renewable and Sustainable Energy Reviews, 137, 110498.

[fsn34313-bib-0242] Velasquez, M. T. , Ramezani, A. , Manal, A. , & Raj, D. S. (2016). Trimethylamine N‐oxide: The good, the bad and the unknown. Toxins, 8(11), 326. 27834801 10.3390/toxins8110326 PMC5127123

[fsn34313-bib-0243] Verma, R. , & Srivastava, A. (2018). Carbon dioxide sequestration and its enhanced utilization by photoautotroph microalgae. Environmental Development, 27, 95–106.

[fsn34313-bib-0244] Vidal, N. P. , Manzanos, M. J. , Goicoechea, E. , & Guillén, M. D. (2016). Farmed and wild sea bass (*Dicentrarchus labrax*) volatile metabolites: A comparative study by SPME‐GC/MS. Journal of the Science of Food and Agriculture, 96(4), 1181–1193. 25851130 10.1002/jsfa.7201

[fsn34313-bib-0245] Vieira, M. V. , Pastrana, L. M. , & Fuciños, P. (2020). Microalgae encapsulation systems for food, pharmaceutical and cosmetics applications. Marine Drugs, 18(12), 644. 33333921 10.3390/md18120644 PMC7765346

[fsn34313-bib-0246] Wan, C. , Alam, M. A. , Zhao, X.‐Q. , Zhang, X.‐Y. , Guo, S.‐L. , Ho, S.‐H. , Chang, J.‐S. , & Bai, F.‐W. (2015). Current progress and future prospect of microalgal biomass harvest using various flocculation technologies. Bioresource Technology, 184, 251–257. 25499148 10.1016/j.biortech.2014.11.081

[fsn34313-bib-0247] Wang, H. , Zhang, J. , Zhu, Y. , Wang, X. , & Shi, W. (2018). Volatile components present in different parts of grass carp. Journal of Food Biochemistry, 42(6), e12668.

[fsn34313-bib-0248] Wang, Q. , Oshita, K. , & Takaoka, M. (2021). Effective lipid extraction from undewatered microalgae liquid using subcritical dimethyl ether. Biotechnology for Biofuels, 14(1), 1–13. 33422122 10.1186/s13068-020-01871-0 PMC7797121

[fsn34313-bib-0249] Wang, W. , Zhou, X. , & Liu, Y. (2020). Characterization and evaluation of umami taste: A review. TrAC Trends in Analytical Chemistry, 127, 115876.

[fsn34313-bib-0250] Wang, X. , Luo, K. , Liu, S. , Adhikari, B. , & Chen, J. (2019). Improvement of gelation properties of soy protein isolate emulsion induced by calcium cooperated with magnesium. Journal of Food Engineering, 244, 32–39.

[fsn34313-bib-0251] Wang, Y. , Tibbetts, S. M. , Berrue, F. , McGinn, P. J. , MacQuarrie, S. P. , Puttaswamy, A. , Patelakis, S. , Schmidt, D. , Melanson, R. , & MacKenzie, S. E. (2020). A rat study to evaluate the protein quality of three green microalgal species and the impact of mechanical cell wall disruption. Food, 9(11), 1531. 10.3390/foods9111531 PMC7694116 33114413

[fsn34313-bib-0252] Wang, Y. , Tibbetts, S. M. , & McGinn, P. J. (2021). Microalgae as sources of high‐quality protein for human food and protein supplements. Food, 10(12), 3002. 10.3390/foods10123002 PMC8700990 34945551

[fsn34313-bib-0253] Wang, Y. , Wang, X. , Lin, C. , Yu, M. , Chen, S. , Guo, J. , Rao, P. , Miao, S. , & Liu, S. (2022). Heat‐induced structural changes in fish muscle collagen related to texture development in fish balls: Using eel ball as a study model. Food Science & Nutrition, 10(2), 329–341. 35154671 10.1002/fsn3.2462 PMC8825713

[fsn34313-bib-0254] Wang, Z. , Wen, X. , Xu, Y. , Ding, Y. , Geng, Y. , & Li, Y. (2018). Maximizing CO_2_ biofixation and lipid productivity of oleaginous microalga Graesiella sp. WBG1 via CO_2_‐regulated pH in indoor and outdoor open reactors. Science of the Total Environment, 619, 827–833. 29734628 10.1016/j.scitotenv.2017.10.127

[fsn34313-bib-1024] Watts, J. E. M. , Schreier, H. J. , Lanska, L. , & Hale, M. S. (2017). The rising tide of antimicrobial resistance in aquaculture: Sources, sinks and solutions. Marine Drugs, 15, 158. 10.3390/md15060158 28587172 PMC5484108

[fsn34313-bib-0255] Wei, M. , Parrish, C. C. , Guerra, N. I. , Armenta, R. E. , & Colombo, S. M. (2021). Extracted microbial oil from a novel *Schizochytrium* sp.(T18) as a sustainable high DHA source for Atlantic salmon feed: Impacts on growth and tissue lipids. Aquaculture, 534, 736249.

[fsn34313-bib-0256] Wells, M. L. , Potin, P. , Craigie, J. S. , Raven, J. A. , Merchant, S. S. , Helliwell, K. E. , Smith, A. G. , Camire, M. E. , & Brawley, S. H. (2017). Algae as nutritional and functional food sources: Revisiting our understanding. Journal of Applied Phycology, 29, 949–982. 28458464 10.1007/s10811-016-0974-5 PMC5387034

[fsn34313-bib-0257] Wild, F. (2016). Manufacture of meat analogues through high moisture extrusion .

[fsn34313-bib-0258] Williams, J. , & Ringsdorf, A. (2020). Human odour thresholds are tuned to atmospheric chemical lifetimes. Philosophical Transactions of the Royal Society B, 375(1800), 20190274. 10.1098/rstb.2019.0274 PMC7209931 32306881

[fsn34313-bib-0259] Williams, P. J. , & Laurens, L. M. (2010). Microalgae as biodiesel & biomass feedstocks: Review & analysis of the biochemistry, energetics & economics. Energy & Environmental Science, 3(5), 554–590.

[fsn34313-bib-1025] Wilson, S. K. , Depczynski, M. , Fisher, R. , Holmes, T. H. , O'Leary, R. A. , & Tinkler, P. (2011). Correction: Habitat associations of juvenile fish at Ningaloo reef, Western Australia: The importance of coral and algae. PLoS One, 6(1). 10.1371/annotation/53a56437-a810-4373-baee-16685ec20b2f PMC2998428 21151875

[fsn34313-bib-0260] Win, T. T. , Barone, G. D. , Secundo, F. , & Fu, P. (2018). Algal biofertilizers and plant growth stimulants for sustainable agriculture. Industrial Biotechnology, 14(4), 203–211.

[fsn34313-bib-0261] Wu, D. , Sun, D.‐W. , & He, Y. (2014). Novel non‐invasive distribution measurement of texture profile analysis (TPA) in salmon fillet by using visible and near infrared hyperspectral imaging. Food Chemistry, 145, 417–426. 24128497 10.1016/j.foodchem.2013.08.063

[fsn34313-bib-1027] Wu, H. , Guo, J. , Yao, Y. , & Xu, S. (2022). Polystyrene nano plastics induced cardiomyocyte apoptosis and myocardial inflammation in carp by promoting ROS production. Fish and Shellfish Immunology, 125, 1–8. 10.1016/j.fsi.2022.04.048 35504440

[fsn34313-bib-0262] Wu, N. , Gu, S. , Tao, N. , Wang, X. , & Ji, S. (2014). Characterization of important odorants in steamed male Chinese mitten crab (*Eriocheir sinensis*) using gas chromatography‐mass spectrometry‐olfactometry. Journal of Food Science, 79(7), C1250–C1259. 24962135 10.1111/1750-3841.12511

[fsn34313-bib-0263] Wu, Q. , Liu, L. , Miron, A. , Klímová, B. , Wan, D. , & Kuča, K. (2016). The antioxidant, immunomodulatory, and anti‐inflammatory activities of spirulina: An overview. Archives of Toxicology, 90, 1817–1840. 27259333 10.1007/s00204-016-1744-5

[fsn34313-bib-0264] Xiao, H. , Li, N. , Yan, L. , & Xue, Y. (2021). The hydration characteristics, structural properties and volatile profile of squid (*Symplectoteuthis oualaniensis*) mantle muscle: Impacts of steaming, boiling, and sous vide cooking. Food, 10(7), 1646. 10.3390/foods10071646 PMC8305883 34359516

[fsn34313-bib-0265] Xiao, R. , Yang, X. , Li, M. , Li, X. , Wei, Y. , Cao, M. , Ragauskas, A. , Thies, M. , Ding, J. , & Zheng, Y. (2018). Investigation of composition, structure and bioactivity of extracellular polymeric substances from original and stress‐induced strains of *Thraustochytrium striatum* . Carbohydrate Polymers, 195, 515–524. 29805006 10.1016/j.carbpol.2018.04.126

[fsn34313-bib-0266] Xu, Y. , & Harvey, P. J. (2019). Carotenoid production by *Dunaliella salina* under red light. Antioxidants, 8(5), 123. 31067695 10.3390/antiox8050123 PMC6562933

[fsn34313-bib-0267] Yaakob, Z. , Ali, E. , Zainal, A. , Mohamad, M. , & Takriff, M. S. (2014). An overview: Biomolecules from microalgae for animal feed and aquaculture. Journal of Biological Research‐Thessaloniki, 21, 1–10. 10.1186/2241-5793-21-6 PMC4376511 25984489

[fsn34313-bib-0268] Yarkent, Ç. , Gürlek, C. , & Oncel, S. S. (2020). Potential of microalgal compounds in trending natural cosmetics: A review. Sustainable Chemistry and Pharmacy, 17, 100304.

[fsn34313-bib-0269] Yarnold, J. , Karan, H. , Oey, M. , & Hankamer, B. (2019). Microalgal aquafeeds as part of a circular bioeconomy. Trends in Plant Science, 24(10), 959–970. 31285128 10.1016/j.tplants.2019.06.005

[fsn34313-bib-0270] Yimdee, T. , & Wang, X.‐C. (2016). Comparison of odor and taste of commercial brand fish sauces from east and south east Asian countries. International Journal of Food Properties, 19(4), 873–896.

[fsn34313-bib-0271] Yin, T. , Yao, R. , Ullah, I. , Xiong, S. , Huang, Q. , You, J. , Hu, Y. , & Shi, L. (2019). Effects of nanosized okara dietary fiber on gelation properties of silver carp surimi. LWT, 111, 111–116.

[fsn34313-bib-0272] Yu, X. , Zhang, L. , Miao, X. , Li, Y. , & Liu, Y. (2017). The structure features of umami hexapeptides for the T1R1/T1R3 receptor. Food Chemistry, 221, 599–605. 27979247 10.1016/j.foodchem.2016.11.133

[fsn34313-bib-0273] Yuliarti, O. , Kovis, T. J. K. , & Yi, N. J. (2021). Structuring the meat analogue by using plant‐based derived composites. Journal of Food Engineering, 288, 110138.

[fsn34313-bib-0274] Zhang, J. , Liu, L. , & Chen, F. (2019). Production and characterization of exopolysaccharides from *Chlorella zofingiensis* and *Chlorella vulgaris* with anti‐colorectal cancer activity. International Journal of Biological Macromolecules, 134, 976–983. 31121230 10.1016/j.ijbiomac.2019.05.117

[fsn34313-bib-0275] Zhang, M.‐X. , Wang, X.‐C. , Liu, Y. , Xu, X.‐L. , & Zhou, G.‐H. (2012). Isolation and identification of flavour peptides from puffer fish (*Takifugu obscurus*) muscle using an electronic tongue and MALDI‐TOF/TOF MS/MS. Food Chemistry, 135(3), 1463–1470. 22953881 10.1016/j.foodchem.2012.06.026

[fsn34313-bib-0276] Zhang, N. , Ayed, C. , Wang, W. , & Liu, Y. (2019). Sensory‐guided analysis of key taste‐active compounds in pufferfish (*Takifugu obscurus*). Journal of Agricultural and Food Chemistry, 67(50), 13809–13816. 30604615 10.1021/acs.jafc.8b06047

[fsn34313-bib-1028] Zhang, R. , Li, L. , Tong, D. , & Hu, C. (2016). Microwave‐enhanced pyrolysis of natural algae from water blooms. Bioresource Technology, 212, 311–317. 10.1016/j.biortech.2016.04.053 27128164

[fsn34313-bib-0277] Zhang, T. , Dou, W. , Zhang, X. , Zhao, Y. , Zhang, Y. , Jiang, L. , & Sui, X. (2021). The development history and recent updates on soy protein‐based meat alternatives. Trends in Food Science & Technology, 109, 702–710.

[fsn34313-bib-1029] Zhao, L. , Khang, H. M. , & Du, J. (2024). Incorporation of microalgae (*Nannochloropsis oceanica*) into plant‐based fishcake analogue: Physical property characterisation and in vitro digestion analysis. Food Hydrocolloids, 146, 109212. 10.1016/j.foodhyd.2023.109212

[fsn34313-bib-0278] Zhou, X. , Chen, T. , Lin, H. , Chen, H. , Liu, J. , Lyu, F. , & Ding, Y. (2019). Physicochemical properties and microstructure of surimi treated with egg white modified by tea polyphenols. Food Hydrocolloids, 90, 82–89.

[fsn34313-bib-0279] Zhou, X. , Chong, Y. , Ding, Y. , Gu, S. , & Liu, L. (2016). Determination of the effects of different washing processes on aroma characteristics in silver carp mince by MMSE–GC–MS, e‐nose and sensory evaluation. Food Chemistry, 207, 205–213. 27080898 10.1016/j.foodchem.2016.03.026

[fsn34313-bib-0280] Zhou, X. , Jiang, S. , Zhao, D. , Zhang, J. , Gu, S. , Pan, Z. , & Ding, Y. (2017). Changes in physicochemical properties and protein structure of surimi enhanced with camellia tea oil. LWT, 84, 562–571.

